# Global, regional, and national trends in tobacco-induced cardiovascular disease burden for 1990–2021 with projections to 2045: A comprehensive analysis based on the Global Burden of Disease Study 2021

**DOI:** 10.18332/tid/204008

**Published:** 2025-05-15

**Authors:** Xiaoqiang Zhu, Lei Chen, Xinyue Yang, Yanyan Du, Yangyu Zhao, Tenglong Hu, Na Sun, Qiang Sun, Wenyan Liang, Xiqing Wei, Zhiqiang Zhang

**Affiliations:** 1 Department of Cardiology, The Second People's Hospital of Hefei, Hefei Hospital Affiliated to Anhui Medical University, Hefei, China; 2 Department of Cardiology, Fujian Medical University Union Hospital, Fuzhou, China; 3 Graduate School of Tianjin Medical University, Tianjin Medical University, Tianjin, China; 4 Department of Cardiology, Affiliated Hospital of Jining Medical University, Jining Medical University, Jining, China

**Keywords:** tobacco-attributable cardiovascular disease, Global Disease Burden Study, sociodemographic disparities, health projections

## Abstract

**INTRODUCTION:**

Tobacco use is a major risk factor for cardiovascular disease (CVD), and its global disease burden trends require further clarification. This study aims to analyze trends in global CVD burden attributable to tobacco in 1990–2021 and project mortality rates and disease burden through 2045.

**METHODS:**

Using Global Burden of Disease Study (GBD) 2021 data, we analyzed temporal trends using age-period-cohort models, evaluated change points with Joinpoint regression, and conducted forecasting using Bayesian age-period-cohort (BAPC) models.

**RESULTS:**

In 2021, tobacco-attributable CVD deaths reached 2.147 million globally (71.3% increase from 1990), although age-standardized mortality rates decreased to 25.36 per 100000 (83.3% reduction). Mortality rates were lowest in high SDI regions (326.71 per 100000) and highest in low-middle sociodemographic index (SDI) regions (788.05 per 100000). The proportion of deaths among those aged ≥80 years increased from 19.2% to 26.2%. Global mortality rates decreased by 1.8% annually, with a greater decline in females (-2.6%) than males (-1.6%). Projections suggest that by 2045, global CVD deaths may reach approximately 3.267 million (52.1% potential increase), although age-standardized mortality rates are expected to decrease to around 38.6 per 100000 (15.9% estimated reduction). Disability-adjusted life years (DALYs) are projected to potentially increase to 75.755 million (39.9% estimated increase), while age-standardized DALY rates could decline to approximately 1008.02 per 100000.

**CONCLUSIONS:**

Between 1990 and 2021, global tobacco-attributable CVD mortality rates showed a declining trend, with notable regional, sex, and age disparities. Projections indicate that while age-standardized rates will continue to decrease, absolute numbers of deaths and disease burden will increase. The findings emphasize the need to strengthen tobacco control and CVD prevention in low-middle SDI regions.

## INTRODUCTION

Cardiovascular disease (CVD) is a major cause of illness and death worldwide, posing significant challenges to public health. The most common fatal CVD conditions are ischemic heart disease (IHD), which occurs when blood flow to the heart is reduced, and ischemic stroke (IS), caused by restricted blood flow to the brain^1^. According to GBD data, CVD causes over 19.41 million deaths each year, representing 29% of global mortality. Tobacco use is one of the leading modifiable risk factors contributing to CVD^2^. Notably, nearly 80% of CVD cases occur in low- and middle-income countries, where aging populations and urbanization accelerate disease progression^3^. As the majority of the global population lives in low- and middle-income countries, urgent interventions are needed to curb the progression of cardiovascular disease CVD.

Epidemiological studies show that active tobacco use significantly increases the risk of adverse cardiovascular outcomes. Additionally, exposure to secondhand smoke raises the relative risk of coronary artery disease by 27% in non-smokers^4^. These findings highlight the essential role of smoking cessation initiatives in preventing cardiovascular disease and guiding intervention strategies. Tobacco use is strongly linked to atherogenesis, hypertension, and impaired vascular endothelial function through well-established mechanisms^5^. Toxicological studies have shown that compounds from cigarettes cause endothelial dysfunction by increasing oxidative stress and activating pro-inflammatory pathways^6^. While substantial evidence establishes the causal link between tobacco exposure and CVD, comprehensive epidemiological analyses of tobacco-attributable CVD morbidity and mortality, stratified by demographic and geographical factors, are still limited in the literature.

In recent years, the Global Burden of Disease (GBD) study has emerged as a vital resource for assessing the burden of CVD through comprehensive data and analytical methods^7,8^. However, most existing studies primarily examine short-term trends and lack systematic analyses of long-term patterns^9^. This study aims to systematically analyze the temporal trends in tobacco-attributable CVD burden from 1990 to 2021, with projections extending to 2045. These findings can provide context for assessing the potential impact of tobacco control measures and help develop more targeted prevention strategies, although it should be recognized that these projections are based on extrapolations of current trends. Using comprehensive GBD 2021 data, the research quantifies tobacco’s impact on CVD and offers region-specific policy recommendations. These results are particularly significant for resource-limited nations, aiding in the efficient allocation of healthcare resources and the improvement of population health outcomes.

## METHODS

### Data sources

The GBD Study 2021 offers a comprehensive evaluation of health loss across 371 diseases in 204 countries and territories from 1990 to 2021. This extensive research effort included a worldwide network of >10000 collaborators from >150 countries, who contributed their expertise in data collection, analysis, and review to generate GBD metrics. The study generated key health metrics, including incidence, prevalence, cause-specific mortality, years of life lost, years lived with disability, and DALYs. Rates are presented per 100000 person-years to enable comparisons across populations.

The GBD study employed a comparative risk assessment method grounded in causal frameworks and a hierarchical classification of risk factors^7^. The framework classifies risk factors into four levels, identifying tobacco as a level 2 risk factor. For this study, tobacco exposure includes both active smoking and secondhand smoke exposure, as defined by the GBD 2021 framework. Active smoking was assessed using metrics of current daily tobacco smoking, while secondhand smoke exposure was evaluated based on average daily exposure to air containing tobacco smoke from others’ tobacco use. CVD, categorized as a level 2 cause, is further divided into ischemic heart disease (IHD, level 3) and ischemic stroke (IS, level 4), with CVD defined in this context as comprising only these two conditions. This expanded definition includes the following specific conditions with their corresponding ICD-10 codes: ischemic heart disease (I20-I25), and ischemic stroke (I63, I63.0–I63.9, I69.3). This study analyzes global trends in tobacco-attributable CVD mortality from 1990 to 2021, utilizing death counts, crude mortality rates (per 100000), age-standardized mortality rates (ASMRs), relative percentage changes, and 95% confidence intervals. Mortality rates are stratified by sex, age, and geographical region, with populations grouped into five age cohorts: 25–49, 50–59, 60–69, 70–79, and ≥80 years. The sociodemographic index (SDI), ranging from 0 to 1, assesses levels of socioeconomic development based on per capita income, education level, and fertility rates among women aged <25 years^10^.

### Statistical analysis

We used the estimated annual percent change (EAPC) to assess temporal trends in smoking-attributable cardiovascular disease mortality in 1990–2021. An age-period-cohort (APC) analysis framework was applied to evaluate the influence of biological, sociological, and technological factors on disease burden. The APC analysis utilized global data of 1992–2021, with age stratified into 5-year groups (from 25–29 to 90–94 years) and periods spanning from 1992–1996 to 2017–2021.

Joinpoint regression was employed to analyze changes in age-standardized cardiovascular disease mortality rates and to calculate average annual percent change (AAPC). The optimal fitting model was determined through Monte Carlo permutation tests, with significance level set at 0.05.

Spearman rank correlation and linear regression analyses were conducted to assess the relationship between SDI and EAPC. Decomposition analysis was applied to identify factors driving changes in death counts and DALYs, examining contributions from population aging, population growth, and epidemiological shifts.

A Bayesian age-period-cohort (BAPC) model was used to project future disease burden, based on a generalized linear model framework and utilizing integrated nested Laplace approximation (INLA) to improve computational efficiency and prediction accuracy.

All estimates in this study are reported with 95% uncertainty intervals (95% UI). Uncertainty intervals quantify the precision of estimates, indicating that there is a 95% probability that the true value lies within this range. These intervals reflect uncertainties arising from data source variability, sample sizes, and model assumptions. Narrower uncertainty intervals indicate higher precision in the estimates, while wider intervals suggest greater uncertainty.

In this study, for estimates directly extracted from the GBD study (such as incidence rates, mortality rates, etc.), we report 95% uncertainty intervals (95% UI), UI for each metric, calculated from the 25th and 95th values of 1000 posterior distribution samples; for results obtained through our own statistical modeling (such as EAPC, Joinpoint regression analyses, etc.), we report 95% confidence intervals (95% CI).

All statistical analyses were performed using R software (version 4.3.1) and trend analysis utilized Joinpoint software version 5.1.0.0. More detailed statistical methods and formulas are provided in the supplementary materials.

## RESULTS

### Global trends in tobacco-attributable CVD burden

In 2021, global tobacco-attributable CVD deaths reached approximately 2147356 (95% UI: 1766455–2569653), marking a substantial 71.3% increase (95% UI: 45. 6–101.8) over the past 32 years. Despite this, the global ASMR in 2021 was 25.36 per 100000 population (95% UI: 20.68–30.43), reflecting a significant 83.3% decrease (95% UI: -93.8 – -70.6) compared to 1990. Tobacco-attributable deaths included 1693084 cases of IHD (95% UI: 1412419–2002974) and 454272 cases of IS (95% UI: 354036–566680), with ASMRs of 19.94 (95% UI: 16.47–23.64) and 5.42 (95% UI: 4.20–6.79) per 100000 population, respectively. The death count and ASMR for IHD were 3.72 times higher than those for IS (Table 1).

**Table 1:** The global trends in the CVD burden attributable to tobacco by sex, CVD subtypes, SDI and WHO Regions from 1990 to 2021

	*Deaths*		*All-age mortality*		*Age-standardized mortality*		
	*Number* *2021* *n (95% UI)*	*Change in number* *1990–2021* *% (95% UI)*	*Rate in 2021* *(per 100000)* *% (95% UI)*	*Percent change* *1990–2021* *% (95% UI)*	*Rate in 2021* *(per 100000)* *% (95% UI)*	*Percent change* *1990–2021* *% (95% UI)*	*Net drift of* *mortality per year* *% (95% CI)*
**Global**	2147356.5 (1766454.6–2569653.4)	71.25 (45.57–101.84)	27.21 (22.38–32.56)	-16.66 (-34.02–4.01)	25.36 (20.68–30.43)	-83.25 (-93.77 – -70.64)	-1.78 (-1.84 – -1.72)
**Sex**							
Female	473079.19 (358829.72–604203.05)	18.36 (-6.33–43.9)	12.03 (9.13–15.37)	-52.95 (-69.57 – -35.75)	10.17 (7.72–12.99)	-107.66 (-117.59 – -97.08)	-2.55 (-2.61– -2.49)
Male	1674277.31 (1396813.73–1992412.1)	92.97 (59.48–135.35)	42.29 (35.28–50.32)	-1.27 (-23.99–27.47)	43.57 (35.89–52.09)	-76.21 (-89.61 – -59.09)	-1.58 (-1.66 – -1.5)
**CVD subtype**							
Ischemic heart disease	1693084.45 (1412418.56–2002973.61)	34.44 (24.94–45.04)	21.45 (17.9–25.38)	-9.13 (-15.55 – -1.97)	19.94 (16.47–23.64)	-40.43 (-44.26 – -35.97)	-1.73 (-1.8 – -1.67)
Ischemic stroke	454272.06 (354036.04–566679.78)	36.81 (20.62–56.8)	5.76 (4.49–7.18)	-7.53 (-18.47–5.98)	5.42 (4.2–6.79)	-42.82 (-49.51 – -34.68)	-1.97 (-2.06 – -1.89)
**SDI**							
High SDI	6009605.93 (5003556.44–7102340.43)	-84.04 (-90.95 – -76.72)	549.3 (457.34–649.18)	-106.78 (-112.33 – -100.89)	326.71 (274.56–381.97)	-133.61 (-137.68 – -129.29)	-3.93 (-3.99 – -3.87)
High-middle SDI	15073168.91 (12530426.82–17907053.11)	35.66 (8.83–66.77)	1155.89 (960.9–1373.21)	-7.8 (-29.68–17.57)	777.27 (647.48–920.94)	-80.17 (-93.73 – -64.96)	-1.85 (-1.92 – -1.78)
Middle SDI	18504615.71 (15198378.96–1903052.84)	164.01 (113.4–224.75)	755.74 (620.71–894.53)	56.13 (20.52–98.87)	682.13 (557.64–811.98)	-57.27 (-76.82 – -34.22)	-0.96 (-1.02 – -0.89)
Low-middle SDI	12064944.76 (9929155.92–4193207.77)	166.41 (124.13–212.37)	628.02 (516.84–738.8)	21.51 (-4.05–49.3)	788.05 (648.38–928.79)	-43.07 (-61.1 – -23.66)	-0.56 (-0.65 – -0.47)
Low SDI	2443410.71 (1953234.53–2966664.18)	150.89 (105.35–198.36)	218.67 (174.8–265.5)	-42.58 (-63.01 – -21.28)	435.66 (348.37–532.23)	-46.11 (-65.82 – -25.43)	-0.79 (-0.93 – -0.64)
**WHO Regions**							
African	1669933.3 (1333841.32–2016880)	145.63 (108.47–188.26)	144.52 (115.43–174.55)	-47.65 (-64.03 – -28.86)	301.63 (240.02–367.6)	-52.58 (-68.27 – -35.16)	-1.2 (-1.32 – -1.09)
Eastern Mediterranean	5910594.03 (4782738.64–7030468.98)	180.28 (125.93–241.93)	784.91 (635.13–933.63)	-10.26 (-37.38–20.5)	1150.57 (927.94–1380.92)	-53.84 (-73.94 – -30.45)	-1.01 (-1.07 – -0.96)
European	9482945.96 (7991218.64–11051025.55)	-73.98 (-81.56 – -66.78)	1015.92 (856.11–1183.91)	-84.13 (-91.1 – -77.5)	634.25 (538–735.25)	-113.14 (-118.52 – -107.74)	-3.07 (-3.16 – -2.98)
Americas	4592659.82 (3823143.76–5422118.02)	-47.53 (-55.92 – -38.63)	447.25 (372.31–528.02)	-93.66 (-99.52 – -87.46)	352.55 (294.32–416.09)	-129.48 (-133.23 – -125.43)	-3.75 (-3.8 – -3.7)
South-East Asia	13695450.73 (11247123.04–6271032.62)	180.83 (129.19–239.6)	663.66 (545.02–788.47)	40.5 (7.89–77.61)	717.34 (586.88–855.75)	-48.91 (-68.86 – -26.25)	-0.74 (-0.87 – -0.6)
Western Pacific	18632263.75 (14783070.68–23036139.52)	181.27 (107.73–273.78)	967.77 (767.84–1196.51)	105.27 (46.39–179.34)	670.84 (533.02–826.91)	-42.96 (-72.96 – -6.36)	-0.39 (-0.49 – -0.3)

The all-age mortality is equivalent to the crude mortality rate. CVD: cardiovascular disease. SDI: sociodemographic index.

From 1990 to 2021, global DALYs attributable to tobacco increased by 61.2% (95% UI: 38.0–88.1), totaling approximately 54145687 (95% UI: 45297067–63318095). In contrast, the global ASDR decreased by 79.5% (95% UI: -89.9 – -67.4). Tobacco-related IHD contributed 43226979 DALYs (95% UI: 36540967–49920797) with an ASDR of 499.59 per 100000 population (95% UI: 421.73–577.91), while IS contributed 10918708 DALYs (95% UI: 8756101–13397298) with an ASDR of 126.7 per 100000 population (95% UI: 101.47–155.29) (Table 2).

**Table 2:** The global trends in the CVD DALYs attributable to tobacco, sex, CVD subtypes, SDI and WHO Regions from 1990 to 2021

	*DALYs*		*All-age DALYs*		*Age-standardized DALYs*		
	*Number* *2021* *n (95% UI)*	*Change in number* *1990–2021* *% (95% UI)*	*Rate in 2021* *(per 100000)* *% (95% UI)*	*Percent change* *1990–2021* *% (95% UI)*	*Rate in 2021* *(per 100000)* *% (95% UI)*	*Percent change* *1990–2021* *% (95% UI)*	*Net drift of* *mortality per year* *% (95% CI)*
**Global**	54145687.1 (45297067.43–63318095.31)	61.24 (37.95–88.09)	686.14 (574.01–802.37)	-23.43 (-39.17 – -5.28)	626.29 (523.19–733.2)	-79.48 (-89.86 – -67.35)	-1.74 (-1.79 – -1.68)
**Sex**							
Female	10321102.08 (8031115.32–12919268.99)	11.39 (-10.5–33.59)	262.49 (204.25–328.57)	-57.65 (-72.39 – -42.69)	225.84 (175.95–282.36)	-103.12 (-112.85 – -92.85)	-2.47 (-2.51 – -2.43)
Male	43824585.02 (37124273.52–51000509.18)	78.69 (47.63–116.34)	1106.85 (937.63–1288.09)	-10.96 (-32.03–14.58)	1068.33 (901.5–1246.55)	-73.21 (-86.55 – -56.87)	-1.54 (-1.62 – -1.46)
**CVD subtype**							
Ischemic heart disease	43226979.48 (36540966.63–9920796.95)	26.67 (18.05–36.22)	547.78 (463.05–632.6)	-14.39 (-20.21 – -7.93)	499.59 (421.73–577.91)	-39.95 (-43.82 – -35.4)	-1.72 (-1.78 – -1.66)
Ischemic stroke	10918707.62 (8756100.8–13397298.36)	34.57 (19.9–51.87)	138.36 (110.96–169.77)	-9.05 (-18.96–2.65)	126.7 (101.47–155.29)	-39.53 (-46.04 – -31.96)	-1.77 (-1.82 – -1.71)
**SDI**							
High SDI	6009605.93 (5003556.44–7102340.43)	-84.04 (-90.95 – -76.72)	549.3 (457.34–649.18)	-106.78 (-112.33 – -100.89)	326.71 (274.56–381.97)	-133.61 (-137.68 – -129.29)	-3.93 (-3.99 – -3.87)
High-middle SDI	15073168.91 (12530426.82–17907053.11)	35.66 (8.83–66.77)	1155.89 (960.9–1373.21)	-7.8 (-29.68–17.57)	777.27 (647.48–920.94)	-80.17 (-93.73 – -64.96)	-1.85 (-1.92 – -1.78)
Middle SDI	18504615.71 (15198378.96–21903052.84)	164.01 (113.4–224.75)	755.74 (620.71–894.53)	56.13 (20.52–98.87)	682.13 (557.64–811.98)	-57.27 (-76.82 – -34.22)	-0.96 (-1.02 – -0.89)
Low-middle SDI	12064944.76 (9929155.92–14193207.77)	166.41 (124.13–212.37)	628.02 (516.84–738.8)	21.51 (-4.05–49.3)	788.05 (648.38–928.79)	-43.07 (-61.1 – -23.66)	-0.56 (-0.65 – -0.47)
Low SDI	2443410.71 (1953234.53–2966664.18)	150.89 (105.35–198.36)	218.67 (174.8–265.5)	-42.58 (-63.01 – -21.28)	435.66 (348.37–532.23)	-46.11 (-65.82 – -25.43)	-0.79 (-0.93 – -0.64)
**WHO Regions**							
African	1669933.3 (1333841.32–2016880)	145.63 (108.47–188.26)	144.52 (115.43–174.55)	-47.65 (-64.03 – -28.86)	301.63 (240.02–367.6)	-52.58 (-68.27 – -35.16)	-1.2 (-1.32 – -1.09)
Eastern Mediterranean	5910594.03 (4782738.64–7030468.98)	180.28 (125.93–241.93)	784.91 (635.13–933.63)	-10.26 (-37.38–20.5)	1150.57 (927.94–1380.92)	-53.84 (-73.94 – -30.45)	-1.01 (-1.07 – -0.96)
European	9482945.96 (7991218.64–11051025.55)	-73.98 (-81.56 – -66.78)	1015.92 (856.11–1183.91)	-84.13 (-91.1 – -77.5)	634.25 (538–735.25)	-113.14 (-118.52 – -107.74)	-3.07 (-3.16 – -2.98)
Americas	4592659.82 (3823143.76–5422118.02)	-47.53 (-55.92 – -38.63)	447.25 (372.31–528.02)	-93.66 (-99.52 – -87.46)	352.55 (294.32–416.09)	-129.48 (-133.23 – -125.43)	-3.75 (-3.8 – -3.7)
South-East Asia	13695450.73 (11247123.04–16271032.62)	180.83 (129.19–239.6)	663.66 (545.02–788.47)	40.5 (7.89–77.61)	717.34 (586.88–855.75)	-48.91 (-68.86 – -26.25)	-0.74 (-0.87 – -0.6)
Western Pacific	18632263.75 (14783070.68–23036139.52)	181.27 (107.73–273.78)	967.77 (767.84–1196.51)	105.27 (46.39–179.34)	670.84 (533.02–826.91)	-42.96 (-72.96 – -6.36)	-0.39 (-0.49 – -0.3)

The all-age mortality is equivalent to the crude mortality rate. CVD: cardiovascular disease. SDI: sociodemographic index. DALYs: disability-adjusted life years.

### Global trends in ASMR and ASDR of CVD attributable to tobacco in relation to SDI

Globally, while deaths from CVD subtypes increased from 1990 to 2021, the ASMR consistently declined (Supplementary file Table 1). By SDI level, the high-SDI group had the lowest ASMR in 2021 at 326.71 per 100000 population (95% UI: 274.56–381.97), a decline of 133.61 (95% UI: -137.68 – -129.29) since 1990. In contrast, the low-middle SDI group recorded the highest ASMR at 788.05 per 100000 population (95% UI: 648.38–928.79), with a smaller decline of 43.07 (95% UI: -61.1– -23.66) over the same period (Table 1).

Among WHO Regions, the Eastern Mediterranean had the highest tobacco-attributable CVD ASMR in 2021 at 1150.57 per 100000 population (95% UI: 927.94–1380.92), while the African had the lowest at 301.63 (95% UI: 240.02–367.6). In all six WHO Regions, male tobacco-attributable CVD ASMR exceeded that of females in 2021, though both sexes saw declines compared to 1990 (Table 2; and Supplementary file Tables 2 and 3).

The analysis found no significant linear correlation between tobacco-related CVD ASMR, ASDR, and SDI. However, mortality rates were lower in high-SDI regions, with ASMR notably declining as SDI increased, particularly in regions with an SDI >0.7 (Supplementary file Figure 19). In regions where SDI was <0.5, the EAPC for tobacco-related CVD ASMR and ASDR remained relatively stable. In contrast, a clear downward trend was observed when SDI >0.5 (Supplementary file Figure 19). Similar patterns between ASMR, ASDR, their corresponding EAPCs, and SDI were observed for both ischemic heart disease (IHD) and ischemic stroke (IS) (Supplementary file Figures 1 and 2).

### Global trends of CVD attributable to tobacco in nations

**Figure 1:**
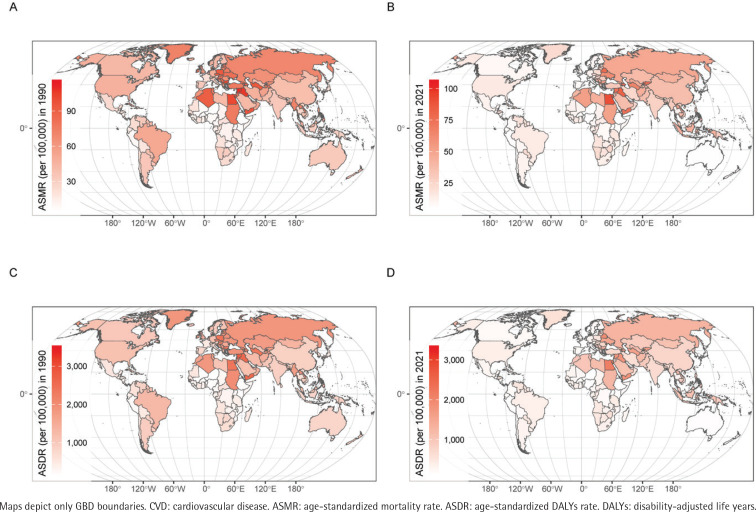
World map of ASMR and ASDR due to CVD attributable to tobacco in 1990 and 2021, and the change of ASMR and ASDR globally from 1990 to 2021: A) ASMR of CVD attributable to tobacco in 1990; B) ASMR of CVD attributable to tobacco in 2021; C) ASDR of CVD attributable to tobacco in 1990; D) ASDR of CVD attributable to tobacco in 2021

The regional distribution of ASMR in 1990 and 2021 showed notable similarities (Figure 1), with higher rates consistently concentrated in developing countries such as Egypt, Iraq, and Kiribati. However, by 2021, several Asian and African countries experienced significant increases in ASMR compared to 1990. In Asia, sharp rises were observed in Kyrgyzstan, Timor-Leste, and Uzbekistan, while in Africa, substantial increases were recorded in Lesotho, Mali, Mozambique, and Zimbabwe.

The geographical distribution of ASDR in 1990 and 2021 closely mirrored the patterns observed for ASMR (Figure 1), with both showing similar national-level dispersion. Compared to 1990, several developing countries experienced marked increases in ASDR by 2021. These increases were particularly pronounced in African countries such as Lesotho, Mali, Mozambique, and Zimbabwe, as well as Asian countries including Kyrgyzstan, Timor-Leste, and Uzbekistan. A more detailed analysis of regional distributions is available in Supplementary file Table S4. Additionally, correlation analyses were performed for ASMR and ASDR in both IHD and IS (Supplementary file Figures 3 and 4).

### Global trends in tobacco-related CVD across age groups

Over the past 32 years, individuals aged ≥80 years accounted for the largest proportion of tobacco-attributable CVD deaths, increasing from 19.2% in 1990 to 26.2% in 2021 (Supplementary file Figure 5). In females, the age group of ≥80 years had significantly higher proportions of tobacco-related CVD mortality compared to other age groups. In males, the age group 60–69 years accounted for the highest proportion of CVD deaths, though this share declined from 28.1% in 1990 to 24.9% in 2021 (Supplementary file Figure 6).

From 1990 to 2021, the age group of 60–69 years consistently contributed the largest share of tobacco-attributable DALYs, ranging from 27% to 29% annually, while the ≥80 years age group had the smallest share (6–11%). However, the proportion of DALYs among individuals aged ≥70 years increased from 26.0% in 1990 to 30.1% in 2021 (Supplementary file Figure 5). The age group of 60–69 years had the highest proportion of DALYs for both females and males (Supplementary file Figure 7).

From 1990 to 2021, mortality rates and DALYs for tobacco-related CVD subtypes declined across age groups <80 years and in both sexes, with particularly notable declines observed in elderly populations. However, individuals aged ≥80 years showed an increasing trend in mortality rates (Supplementary file Figure 5). Among those aged ≥85 years, women experienced more significant declines in mortality rates from tobacco-related CVD subtypes compared to men. Conversely, individuals <60 years, in both men and women, exhibited lower mortality rates but smaller declines over time (Supplementary file Figure 8).

Global DALYs for tobacco-related CVD subtypes during the same period followed similar age-specific and temporal trends as mortality rates (Supplementary file Figure 9).

### Impact of local drifts, age, period, and cohort effects on tobacco-attributable CVD burden

Globally, the net drift in CVD mortality attributable to tobacco was -1.8% per year (95% CI: -1.8 – -1.7). The annual net drift was higher for females (-2.6%; 95% CI: -2.6 – -2.5) than for males (-1.6%; 95% CI: -1.7 – -1.5). Among CVD subtypes, the net drift in IS mortality (-2.0% per year; 95% CI: -2.1 – -1.9) exceeded that of IHD mortality (-1.7% per year; 95% CI: -1.8 – -1.7) (Table 1). Declining mortality trends were observed across sex, age groups, and CVD subtypes (Supplementary file Figure 10). All WHO Regions and SDI areas also demonstrated decreases in cardiovascular mortality attributable to tobacco use. The Americas showed the steepest decline (-3.8% per year; 95% CI: -3.8 – -3.7), while the Western Pacific had the lowest net drift (-0.4% per year; 95% CI: -0.5 – -0.3). High SDI regions experienced the largest annual decrease (-3.9%; 95% CI: -4.0 – -3.9), compared to -1.9% (95% CI: -1.9 – -1.8) in high-middle SDI regions. In contrast, low and low-middle SDI regions exhibited smaller declines in CVD-related mortality (Table 1).

Mortality rates increased with advancing age, but a noticeable slowing of the increase was observed in individuals aged ≥90 years. Among males, mortality rates stabilized at older ages, whereas females showed a continued increase (Supplementary file Figure 10). Tobacco-related CVD DALYs followed similar trends to mortality rates (Supplementary file Figure 11). Regarding period and cohort effects, both CVD-related mortality and DALYs demonstrated consistent declines for males and females, with a more pronounced reduction in females (Supplementary file Figures 10 and 11).

Between 1990 and 2021, the ASDR for CVD among tobacco users showed an overall downward trend, divided into five phases of decline. The sharpest decrease occurred between 2004 and 2007 (-2.9%), while the slowest occurred between 1990 and 1994 (-0.2%). The total AAPC over the 32 years was -1.7% (Supplementary file Figure 12). For tobacco-attributable IHD, the ASDR declined in four phases, with the largest reduction occurring between 1994 and 1998 and the slowest between 1990 and 1994. Similarly, IS ASDR exhibited six phases of decline, with the steepest decrease between 2004 and 2007 (-4.1%) and the smallest between 1990 and 1995 (-0.4%). Overall, the AAPC for IHD (-1.7%) was slightly lower than for IS (-1.8%) (Supplementary file Figure 12). Notably, throughout the 32-year period, the AAPC for CVD-related mortality was consistently lower in males than females (Supplementary file Figure 13).

Between 1990 and 2021, DALYs for CVD subtypes among smokers showed an overall downward trend, characterized by five distinct phases. The steepest decline (-2.8%) occurred during 2004–2007, while the smallest reduction (-0.2%) was observed during 1990–1994. The average annual decrease over the 32-year period was -1.6%.

For specific disease types, IHD DALYs declined in four phases, with the largest decrease during 1994–1998 and the smallest during 1990–1994, resulting in an overall average annual decrease of -1.64%. IS DALYs exhibited six phases of decline, with the steepest decrease (-3.5%) during 2004–2007 and the smallest (-0.18%) during 1990–1995, showing an overall annual decrease of -1.6%, slightly lower than IHD (Supplementary file Figure 14).

Notably, from 1990 to 2021, the AAPC of CVD-related DALYs in males consistently remained lower than in females (Supplementary file Figure 15).

### Estimated trends of CVD attributable to tobacco until 2045

Using the BAPC model, we projected the global CVD burden for 2021–2045. By 2045, global CVD deaths are estimated to reach 3267256 (95% UI: 0–6903136) (Supplementary file Figure 20), with the ASMR projected to decline to 38.62 per 100000 people (95% UI: 0–81.78). Compared to 2021, all-age deaths are expected to increase by 52.1%, while the ASDR will decrease by 15.9%.

By sex, male all-age deaths are projected to rise by 54.6%, accompanied by a 25.0% decline in ASMR, while female deaths are expected to increase by 43.5%, with a 35.6% decline in ASMR. Male deaths are anticipated to exceed female deaths, with male death rates declining more slowly, contributing to the overall reduction in total death rates (Supplementary file Figure 16).

Projections also indicate a continuous increase in tobacco-attributable DALYs. By 2045, DALYs are projected to reach 75755408 (95% UI: 169904–157002131), an increase of 21609847 from 2021, representing a 39.94% growth. The ASDR is expected to decrease to 1008.02 per 100000 people (95% UI: 1.99–2094.89) (Supplementary file Figure 21). Male DALYs are projected to increase by 42.5%, with the ASDR declining by 19.6%. Female DALYs are expected to rise by 28.9%, with the ASDR decreasing by 32.7% (Supplementary file Figure 17). By 2045, the DALYs burden for males is projected to exceed that for females.

## DISCUSSION

This study analyzed long-term trends in CVD mortality and DALYs attributable to tobacco at global, regional, and national levels from 1990 to 2021. The findings reveal that, while the global ASMR of tobacco-induced CVD has declined significantly, the absolute number of deaths remains substantial. This trend is likely driven by the interplay of population growth, aging, and tobacco exposure.

In 2021, global tobacco-attributable CVD deaths reached 2.15 million, representing a 71.25% increase from 1990. Projections estimate this value will rise to 3.27 million by 2045, marking a 52.1% growth. Despite this increase in absolute deaths, the global ASMR in 2021 was 25.36 per 100000 population, an 83.25% decrease from 1990. By 2045, the ASMR is expected to decline further to 38.62 per 100000, representing a 15.9% reduction. Similarly, tobacco-related DALYs totaled 75.76 million in 2021, a 39.94% increase from 1990, and are projected to remain at this level by 2045, though age-standardized DALYs rates will continue to decline.

These findings highlight a critical trend: while relative measures such as ASMR and age-standardized DALYs rates have decreased due to advancements in public health and tobacco control measures, the absolute burden of tobacco-related CVD continues to rise. This is primarily driven by rapid global population growth and aging, which have led to increased absolute numbers of tobacco-related CVD deaths despite declining per capita rates^11^.

In 2021, high-SDI regions had the lowest ASMR for tobacco-related CVD at 326.71 per 100000, with a rapid annual mortality decline and a net drift of -3.93%. In contrast, middle-low SDI regions recorded the highest ASMR at 788.05 per 100000, with slower declines and lower net drift, reflecting the limited effectiveness of tobacco control and CVD prevention measures. Among WHO Regions, the Eastern Mediterranean had the highest ASMR (1150.57 per 100000), while the African had the lowest (301.63 per 100000).

The lower ASMR in high-SDI regions is largely due to effective tobacco control policies, including high tobacco taxes and smoking bans, which have significantly reduced smoking rates and the relative CVD burden^12,13^. In contrast, middle-low SDI regions face economic constraints and weak policy enforcement, resulting in slower reductions in smoking prevalence and higher disease burdens^14^. Furthermore, rapid population growth and an increasing proportion of elderly individuals in middle-low SDI regions have contributed to the rising absolute burden of tobacco-related CVD in these areas11,12.

The decline in mortality rates was slower in males (annual net drift: -1.58%) compared to females (annual net drift: -2.55%). All-age death counts are projected to increase by 54.6% in males, surpassing the 43.5% increase in females. While tobacco control measures have been more effective among females, males continue to bear a higher mortality and DALYs burden.

The proportion of deaths among individuals aged ≥80 years increased from 19.2% in 1990 to 26.2% in 2021. Between 1990 and 2021, this population experienced rising CVD mortality rates and DALYs, whereas those <80 years showed declining trends in both measures. The elderly population, particularly those aged ≥80 years, faces a significantly higher CVD mortality burden due to aging-related vulnerabilities and physiological factors^15,16^. With advances in medical technology and increased life expectancy, the proportion of the elderly population (aged ≥60 years, especially those aged ≥80 years) has risen substantially, placing them at higher risk for CVD^17^. Furthermore, elderly individuals experience more pronounced cumulative effects of tobacco-related cardiovascular injuries, including atherosclerosis and hypertension^5,11,16^.

In 2021, tobacco-attributed ischemic heart disease (IHD) deaths reached 16.93 million, comprising the majority of total CVD mortality, while tobacco-related ischemic stroke (IS) accounted for 4.54 million deaths, with an age-standardized mortality rate (ASMR) of 5.42 per 100000 population. The ASMR for IHD (19.94 per 100000) was 3.72 times higher than that of IS. Mortality rates showed a net annual decline of -1.73% for IHD and -1.97% for IS, with IS experiencing a faster reduction. This trend may be attributed to significant advances in acute stroke treatments, including thrombolysis, anticoagulation, and interventional therapies, while chronic management of IHD – such as coronary stenting and long-term medication – has had slower impacts on mortality reduction^18-21^.

IHD’s higher mortality rate and disease burden are largely due to its greater incidence and stronger susceptibility to population aging^15,17,21,22^. Smoking has a more pronounced impact on IHD, significantly increasing risk through mechanisms such as arterial hardening, inflammatory responses, and coronary artery spasm^23^. Moreover, the reduction in IHD risk following smoking cessation requires substantial time (approximately 5–10 years), contributing to the slower decline in the tobacco-related IHD burden^24,25^. Conversely, while smoking has a relatively weaker direct effect on IS, passive smoking increases stroke risk by approximately 27%^26,27^. IS responds more rapidly to tobacco control measures, particularly in high-income countries where stringent policies have effectively reduced the stroke burden^28,29^.

Compared to existing studies, our findings demonstrate high consistency but also highlight some differences. For example, one study reported a 30% decline in global CVD mortality rates from 2000 to 2019^30^, whereas our analysis indicates an 83.25% reduction between 1990 and 2021. These discrepancies can likely be attributed to variations in study periods, data sources, and methodological approaches.

Reducing tobacco use and regulating harmful substances in tobacco products is essential for lowering CVD mortality rates. Our findings reveal that the highest proportion of smoking-attributed CVD mortality occurs in populations aged ≥80 years, likely due to age-related physiological decline and the cumulative effects of smoking exposure^31^. Notably, this study observed a greater decline in smoking-related CVD mortality among elderly women (aged ≥85 years), which may be attributed to the cardiovascular protective effects of estrogen, lower smoking prevalence among women, or a later onset of smoking, resulting in reduced cumulative health risks compared to men^32^.

Additionally, a non-linear relationship was identified between the SDI and CVD burden, with a substantial decline in mortality rates at SDI values >0.7. However, middle- and low-SDI regions continue to experience a high CVD burden, reflecting disparities in global health resources and underscoring the strong link between socioeconomic development and the capacity to control cardiovascular diseases^33^.

In recent years, many countries have implemented tobacco control policies, such as increasing tobacco taxes, banning indoor smoking, and promoting smoking cessation services, which have likely contributed to the decline in CVD mortality rates. Public health education and awareness campaigns have further enhanced understanding of tobacco-related risks and encouraged smoking cessation behaviors^34^. Additionally, advancements in medical care and technology have played a significant role in reducing CVD mortality. Progress in diagnostic and treatment technologies, such as coronary interventions and heart transplantation, has improved survival rates and quality of life for CVD patients. Demographic shifts and lifestyle changes may also have contributed to this decline. For example, population aging and urbanization in many countries have led to lifestyle modifications, including changes in dietary and exercise habits.

### Strengths and limitations

This study provides an important contribution by presenting updated data on global tobacco-related CVD mortality trends and deaths from 1990 to 2021, utilizing the GBD 2021 framework. This framework ensures consistency with previous GBD methodologies while offering a more detailed analysis of tobacco’s specific impacts on CVD, particularly through trends across different age groups and sex. By employing the age-period-cohort model, we analyzed CVD mortality trends and examined the influence of period and cohort effects on these changes. Our projections through 2045 provide valuable insights for future public health planning. However, our study has several important limitations. As a secondary analysis based on the GBD 2021 database, our research includes the inherent limitations of GBD methodology, including potential uncertainties in data collection, modeling assumptions, and estimation procedures. Our analysis does not differentiate between types of tobacco use or intensity of smoking, which could provide more detailed insights into risk patterns. While we included secondhand smoke exposure, its full impact may be underestimated due to challenges in exposure assessment. The assumptions underlying our regression analyses and EAPC estimations may not fully capture complex temporal patterns. Finally, our analysis is based on ecological and cross-sectional data, which limits our ability to establish direct causal relationships, and the GBD database may not fully capture all relevant socioeconomic, cultural, and healthcare system factors influencing tobacco use patterns and cardiovascular disease outcomes across different populations.

## CONCLUSIONS

Through analysis of global tobacco-attributable cardiovascular disease burden from 1990 to 2021, and projections through 2045, this study found that while mortality rates will continue to decline, absolute death numbers will remain significant. The research revealed marked disparities across regions, age groups, and sexes, with particular challenges in middle- and low-SDI areas. These findings emphasize the necessity of targeted interventions, provide valuable reference for policymakers, support global tobacco control and cardiovascular disease prevention efforts, and help reduce health inequalities while guiding future public health strategies.

## Supplementary Material



## Data Availability

The data supporting this research are available from the Global Health Data Exchange online at https://vizhub.healthdata.org/gbd-results/
