# Supplementary material for: Global, regional, and national trends in tobacco-induced cardiovascular disease burden for 1990–2021 with projections to 2045: A comprehensive analysis based on the Global Burden of Disease Study 2021
Source: Tob Induc Dis. 2025 May 15;23:10.18332/tid/204008. doi: 10.18332/tid/204008 (PMC12080230; doi:10.18332/tid/204008)
Supplement: Supplementary file 1 [file TID-23-63-s1.pdf]

Global, Regional, and National Trends in Tobacco-Induced Cardiovascular Disease Burden from 1990 to 2021: A Comprehensive Analysis Based on the Global Burden of Disease Study 2021 with Projections to 2045

Trends in Tobacco-Related Cardiovascular Disease

Supplemental Material

Content of Supplemental Material

Supplemental methods

Supplemental Tables

TableS1. The global trends in the Subtypes of CVD burden attributable to tobacco by SDI and region from 1990 to 2021.
Table S2. The global trends in the male CVD burden attributable to tobacco by SDI and region from 1990 to 2021.
Table S3. The global trends in the female CVD burden attributable to tobacco by SDI and region from 1990 to 2021.
Table S4. The global trends in burden attributable to tobacco by different countries from 1990 to 2021.

Supplemental Figures

Figure S1. The change of ASMR and ASDR related to ischemic heart disease attributable to tobacco with SDI changes. (A) ASMR and SDI by GBD location from 1990 to 2021, (B) EAPC in ASMR and SDI in 2021 by Super GBD region, (C) ASDR and SDI by GBD location from 1990 to 2021, (D) EAPC in ADMR and SDI in 2021 by Super GBD region.
Figure S2. The change of ASMR related to ischemic stroke attributable to tobacco with SDI changes (A) ASMR and SDI by GBD location from 1990 to 2021, (B) EAPC in ASMR and SDI in 2021 by Super GBD region, (C) ASDR and SDI by GBD location from 1990 to 2021, (D) EAPC in ADMR and SDI in 2021 by Super GBD region.
Figure S3. World map of ASMR and ASDR to CVD attributable to tobacco in 1990 and 2021, and the change of ASMR and ASDR globally from 1990 to 2021. (A) ASMR of CVD attributable to tobacco in 1990. (B) ASMR of CVD attributable to tobacco in 2021. (C) ASDR of CVD attributable to tobacco in 1990. (D) ASDR of CVD attributable to tobacco in 2021.
Figure S4. World map of ASMR and ASDR of ischemic heart disease to tobacco in 1990 and 2021, and the change of ASMR and ASDR globally from 1990 to 2021. (A) ASMR of ischemic heart disease attributable to tobacco in 1990. (B) ASMR of ischemic heart disease attributable to tobacco in 2021. (C) ASDR of ischemic heart disease attributable to tobacco in 1990. (D) ASDR of ischemic heart disease attributable to tobacco in 2021.
Figure S5. World map of ASMR and ASDR of ischemic stroke disease to tobacco in 1990 and 2021,

and the change of ASMR and ASDR globally from 1990 to 2021. (A) ASMR of ischemic stroke attributable to tobacco in 1990. (B) ASMR of ischemic stroke attributable to tobacco in 2021. (C) ASDR of ischemic stroke attributable to tobacco in 1990. (D) ASDR of ischemic stroke attributable to tobacco in 2021.

Figure S6. The temporal change of the mortality rate and DALYs attributed to CVD attributable to tobacco across age groups in global from 1990 to 2021. (A-C) The relative proportion of CVD related to mortality, (D-F) The temporal changes in the mortality rate of CVD, (G-I) The relative proportion of CVD related to DALYs, (J-L), The temporal changes in the DALYs rate of CVD. Abbreviations: CVD, cardiovascular disease. DALYs, disability-adjusted life years

Figure S7. The temporal change in the sex-specific relative proportion of CVD deaths attributable to tobacco across age groups in global from 1990 to 2021.

Figure S8. The temporal change in the sex-specific relative proportion of CVD DALYs attributable to tobacco across age groups in global from 1990 to 2021.

Figure S9. The temporal change in the sex-specific deaths rate for CVD attributable to tobacco across age groups in global from 1990 to 2021.

Figure S10. The temporal change in the sex-specific deaths rate for CVD attributable to tobacco across age groups in global from 1990 to 2021.

Figure S11. The local drifts, age effects, period effects, and cohort effects of CVD-related mortality attributable to tobacco in global from 1990 to 2021. (A) The local drift analysis of CVD, (B) The age effect of CVD, (C) The period effect of CVD, (D) The cohort effect of CVD, (E) The local drift analysis of ischemic heart disease, (F) The age effect of ischemic heart disease, (G) The period effect of ischemic heart disease, (H) The cohort effect of ischemic heart disease, (I) The local drift analysis of ischemic stroke, (J) The age effect of ischemic stroke, (K) The period effect of ischemic stroke, (L) The cohort effect of ischemic stroke.

Figure S12. The local drifts, age effects, period effects, and cohort effects of CVD-related DALYs attributable to tobacco in global from 1990 to 2021. (A) The local drift analysis of CVD, (B) The age effect of CVD, (C) The period effect of CVD, (D) The cohort effect of CVD, (E) The local drift analysis of ischemic heart disease, (F) The age effect of ischemic heart disease, (G) The period effect of ischemic heart disease, (H) The cohort effect of ischemic heart disease, (I) The local drift analysis of ischemic stroke, (J) The age effect of ischemic stroke, (K) The period effect of ischemic stroke, (L) The cohort effect of ischemic stroke.

Figure S13. The joinpoint regression analysis of the age -standardized death rate for CVD attributable to tobacco in global from 1990 to 2021. (A) The joinpoint regression analysis of the age -standardized death rate for CVD, (B) The joinpoint regression analysis of the age -standardized death rate for ischemic heart disease, (C) The joinpoint regression analysis of the age -standardized death rate for ischemic stroke.

Figure S14. The joinpoint regression analysis of the sex-specific age-standardized death rate for CVD attributable to tobacco in global from 1990 to 2021. (A) The joinpoint regression analysis of the male age -standardized death rate for CVD, (B) The joinpoint regression analysis of the female age -standardized death rate for CVD, (C) The joinpoint regression analysis of the male age -standardized death rate for ischemic heart disease, (D) The joinpoint regression analysis of the female age -standardized death rate for ischemic heart disease, (E) The joinpoint regression analysis of the male age -standardized death rate for ischemic stroke (E) The joinpoint regression analysis of the female age -standardized death rate for ischemic stroke.

Figure S15. The joinpoint regression analysis of the age -standardized DALYs rate for CVD attributable to tobacco in global from 1990 to 2021. (A) The joinpoint regression analysis of the age -standardized DALYs rate for CVD, (B) The joinpoint regression analysis of the age -standardized DALYs rate for ischemic heart disease, (C) The joinpoint regression analysis of the age -standardized DALYs rate for ischemic stroke.

Figure S16. The joinpoint regression analysis of the sex-specific age-standardized DALYs rate for CVD attributable to tobacco in global from 1990 to 2021. (A) The joinpoint regression analysis of the male age -standardized DALYs rate for CVD, (B) The joinpoint regression analysis of the female age -standardized DALYs rate for CVD, (C) The joinpoint regression analysis of the male age -standardized DALYs rate for ischemic heart disease, (D) The joinpoint regression analysis of the female age -standardized DALYs rate for ischemic heart disease, (E) The joinpoint regression analysis of the male age -standardized DALYs rate for ischemic stroke (E) The joinpoint regression analysis of the female age -standardized DALYs rate for ischemic stroke.

Figure S17. Temporal trend in the number of death cases and ASMR for CVD from 1990 to 2045 for both males and females. (A) Temporal trend in the number of death cases and ASMR for CVD for males, (B) Temporal trend in the number of death cases and ASMR for CVD for females, (C) Temporal trend in the number of death cases and ASMR for ischemic heart disease for males, (D) Temporal trend in the number of death cases and ASMR for ischemic heart disease for females, (E) Temporal trend in the number of death cases and ASMR for ischemic stroke for males, (F) Temporal trend in the number of death cases and ASMR for ischemic stroke for females.

Solid lines represent observed ASMR, and dashed lines represent ASMR predicted by the BAPC model.

Figure S18. Temporal trend in the number of DALYs cases for CVD from 1990 to 2045 for both males and females. (A) Temporal trend in the number of DALYs and ASDR for CVD for males, (B) Temporal trend in the number of DALYs and ASDR for CVD for females, (C) Temporal trend in the number of DALYs and ASDR for ischemic heart disease for males, (D) Temporal trend in the number of DALYs and ASDR for ischemic heart disease for females, (E) Temporal trend in the number of DALYs and ASDR for ischemic stroke for males, (F) Temporal trend in the number of DALYs and ASDR for ischemic stroke for females.

Figure S19. The change of ASMR and ASDR related to CVD attributable to tobacco with SDI changes. (A) ASMR and SDI by GBD location from 1990 to 2021, (B) EAPC of ASMR and SDI in 2021 by Super GBD region, (C) ASDR and SDI by GBD location from 1990 to 2021, (D) EAPC in ADMR and SDI in 2021 by Super GBD region

Abbreviations: CVD, cardiovascular disease. SDI, socio-demographic index. ASMR, age-standardized mortality rate. ASDR, age-standardized DALYs rate, DALYs, disability-adjusted life years, EAPC, estimated annual percentage change.

Figure S20. Temporal trend in the number of mortality cases and ASMR for CVD from 1990 to 2045. (A) Temporal trend in the number of mortality and ASMR for CVD from 1990 to 2045, (B) Temporal trend in the number of mortality and ASMR for ischemic heart disease from 1990 to 2045, (C) Temporal trend in the number of mortality and ASMR for ischemic stroke from 1990 to 2045.

Solid lines represent observed ASMR, and dashed lines represent ASMR predicted by the BAPC model

Abbreviations: CVD, cardiovascular disease. ASMR, age-standardized mortality rate. BAPC, Bayesian age-period-cohort.

Figure S21. Temporal trend in the number of DALYs and ASDR for CVD from 1990 to 2045. (A) Temporal trend in the number of DALYs and ASDR for CVD from 1990 to 2045, (B) Temporal trend in the number of DALYs and ASDR for ischemic heart disease from 1990 to 2045, (C) Temporal trend in the number of DALYs and ASDR for ischemic stroke from 1990 to 2045.
Solid lines represent observed ASMR, and dashed lines represent ASMR predicted by the BAPC model
Abbreviations: CVD, cardiovascular disease. ASDR, age-standardized DALYs rate, DALYs, disability-adjusted life years, BAPC, Bayesian age-period-cohort.

Supplemental Methods

The estimated annual percent change (EAPC) is a commonly used epidemiological metric that quantifies yearly percentage changes in disease incidence or mortality rates over a specified time period. For ASMR and age-standardized death rates (ASDR), EAPC is calculated using a log-linear regression model, which fits the natural logarithm of rates to time.

The model follows the formula: $In(ASR)=\beta 0+\beta 1\times year+\varepsilon$, where $In(ASR)$ is the natural logarithm of age-standardized rates, $\beta 0$ is the intercept, $\beta 1$ represents the annual rate of change, and ε is the error term. The temporal variable (year) spans from 1990 to 2021.

The analysis employs an age-period-cohort (APC) framework to evaluate the influence of biological, sociological, and technological factors on the cardiovascular disease burden.[1] While the linear dependency among age, period, and cohort parameters (cohort = period - age) makes independent estimation mathematically challenging, the APC method addresses this interdependence. It disentangles these temporal components, allowing simultaneous assessment of their effects on disease mortality.[2]

The APC analysis utilized global population data on tobacco-attributable cardiovascular disease mortality from 1992 to 2021, excluding 1990–1991 to maintain quinquennial intervals. Age was stratified into 5-year groups (25–29 to 90–94 years), with six temporal periods spanning 1992–1996 (median: 1994) to 2017–2021 (median: 2019). Birth cohorts ranged from 1900–1904 (median: 1902) to 1965–1969 (median: 1967).

The APC model generated net and local drift estimates for cardiovascular mortality. Net drift measured the overall annual percent change in logarithmic mortality rates, accounting for period and cohort effects. Local drift represented age-specific annual percent changes in logarithmic mortality rates for each age group.[3]

Joinpoint regression was used to analyze the APC in age-standardized cardiovascular disease (CVD) mortality rates from 1990 to 2021, along with the overall average annual percent change (AAPC).[4] The model employed segmented regression based on the log-linear equation $In(y)=\beta\times x+constant.$ Potential joinpoints were identified using the grid search method (GSM), with optimal points determined by minimizing the mean squared error (MSE). Monte Carlo permutation tests, allowing 0 to 5 joinpoints, identified the best-fitting model.

The final model estimated APC, AAPC, and 95% confidence intervals (CI) for global and SDI-specific regions to evaluate mortality trends and detect turning points. This approach highlights trend changes, aiding in understanding mortality shifts and informing targeted interventions. Statistical significance was assessed using chi-square tests at a 0.05 significance level. Data analysis was conducted in R version 4.3.2, and trend analysis utilized Joinpoint software version 5.1.0.0.The APC calculation formula is: $APC=(e-1) \times 100\%$. ~~$APC = (e - 1) \times 100\%$~~

In the equation $In(y)=\beta * x+constant$, the regression coefficient β represents the instantaneous rate of change. The Average Annual Percent Change (AAPC) is calculated as a weighted mean of segment-specific APCs using the formula: $AAPC = exp(\sum(w_i \times ln(1 + APC_i/100)) - 1) \times 100\%$.

where w_i is the temporal weight of the **i-th** segment, and **APC_i** is the segment-specific annual percent change. This method enhances the precision of temporal trend detection while maintaining model stability. The EAPC can be calculated using the following formula: $EAPC = (exp(\beta) - 1) \times 100$.

The regression slope β quantifies the temporal rate of change in the natural logarithm of ASR, while $\exp(\beta)$ represents the proportional annual change in ASR. The Estimated Annual Percent Change (EAPC) specifically quantifies the temporal trajectory of both age-standardized mortality rates (ASMR) and age-standardized death rates (ASDR), providing standardized metrics for mortality trend analysis. The 95% CI is calculated using: $95\%\ CI\ of\ EAPC = (e\^{}(\beta \pm SE \times 1.96) - 1) \times 100\%$.

Here, SE represents the standard error of β. A positive EAPC with a 95% CI lower bound above 0 indicates an increasing trend in ASR, while a negative EAPC with a 95% CI upper bound below 0 reflects a decreasing trend.

Spearman rank correlation and linear regression analyses were conducted to assess the relationship between SDI and EAPC for ASMR and ASDR. Decomposition analysis using validated algorithms was applied to identify factors driving changes in death counts and DALYs. The analysis examined the contributions of aging, population growth, and epidemiological shifts, assigning values to each factor. Positive values indicate contributing factors, whereas negative values denote inhibitory effects.

The study employed the BAPC model to project disease burden, leveraging its ability to manage the complex, high-dimensional, and sparse datasets typical of large-scale epidemiological

databases such as GBD 2021.[5] The BAPC model is based on a generalized linear model (GLM) framework within a Bayesian context, enabling dynamic integration of age, period, and cohort effects. These effects are assumed to evolve over time and are smoothed using second-order random walks, yielding more accurate posterior probability predictions. A key advantage of the BAPC model is its use of integrated nested Laplace approximation (INLA) to approximate marginal posterior distributions. This approach overcomes challenges commonly associated with Markov Chain Monte Carlo methods, such as mixing and convergence issues, while maintaining computational efficiency. The model's flexibility and robustness in handling time series data make it particularly well-suited for long-term disease burden forecasting.

The BAPC model has been extensively validated and widely applied in epidemiological research, especially for studies involving age-structured populations and complex cohort effects. This approach enables detailed forecasting of future disease burden while accounting for the intricate interplay of age, period, and cohort effects.

Reference

1. Bell A. Age period cohort analysis: a review of what we should and shouldn't do. *Ann Hum Biol*. Mar 2020;47(2):208-217. doi:10.1080/03014460.2019.1707872
2. Su Z, Zou Z, Hay SI, et al. Global, regional, and national time trends in mortality for congenital heart disease, 1990-2019: An age-period-cohort analysis for the Global Burden of Disease 2019 study. *EClinicalMedicine*. Jan 2022;43:101249. doi:10.1016/j.eclinm.2021.101249
3. Rosenberg PS, Check DP, Anderson WF. A web tool for age-period-cohort analysis of cancer incidence and mortality rates. *Cancer Epidemiol Biomarkers Prev*. Nov 2014;23(11):2296-302. doi:10.1158/1055-9965.EPI-14-0300
4. Kim HJ, Fay MP, Feuer EJ, Midthune DN. Permutation tests for joinpoint regression with applications to cancer rates. *Stat Med*. Feb 15 2000;19(3):335-51. doi:10.1002/(sici)1097-0258(20000215)19:3<335::aid-sim336>3.0.co;2-z
5. Knoll M, Furkel J, Debus J, Abdollahi A, Karch A, Stock C. An R package for an integrated evaluation of statistical approaches to cancer incidence projection. *BMC Med Res Methodol*. Oct 15 2020;20(1):257. doi:10.1186/s12874-020-01133-5

TableS1. The global trends in the Subtypes of CVD burden attributable to tobacco by SDI and region from 1990 to 2021

Characteristics	Deaths		All-age mortality		Age-standardized mortality		Net drift of mortality, % per year
	Number in 2021, n	Change of numbers 1990-2021, %	Rate in 2021, per 100 000	Percent change 1990-2021, %	Rate in 2021, per 100 000	Percent change 1990-2021, %	
**Global**							
Ischaemic heart disease	34.44 (24.94 to 45.04)	21.45 (17.9 to 25.38)	-9.13 (-15.55 to -1.97)	19.94 (16.47 to 23.64)	-40.43 (-44.26 to -35.97)	-1.73 (-1.8 to -1.67)	34.44 (24.94 to 45.04)
Ischemic stroke	454272.06 (354036.04 to 566679.78)	36.81 (20.62 to 56.8)	5.76 (4.49 to 7.18)	-7.53 (-18.47 to 5.98)	5.42 (4.2 to 6.79)	-42.82 (-49.51 to -34.68)	-1.97 (-2.06 to -1.89)
**African Region**							
Ischaemic heart disease	44919.46 (36056.25 to 53949.68)	77.86 (59.8 to 96.46)	3.89 (3.12 to 4.67)	-21.6 (-29.56 to -13.4)	9.54 (7.48 to 11.75)	-21.67 (-29.93 to -13.37)	-1.18 (-1.34 to -1.02)
Ischemic stroke	12799.45 (9578.99 to 16580.48)	66.89 (45.77 to 93.14)	1.11 (0.83 to 1.43)	-26.44 (-35.75 to -14.87)	3.26 (2.41 to 4.24)	-27.34 (-36.23 to -17.45)	-1.21 (-1.37 to -1.05)
**Eastern Mediterranean Region**							
Ischaemic heart disease	169990.8 (137865.78 to 202643.99)	90.06 (67.01 to 118.76)	22.57 (18.31 to 26.91)	-5.17 (-16.67 to 9.15)	38.55 (30.69 to 46.31)	-25.11 (-33.55 to -14.26)	-1 (-1.05 to -0.95)
Ischemic stroke	29702.89 (22822.9 to 38136.62)	78.85 (49.57 to 113.85)	3.94 (3.03 to 5.06)	-10.76 (-25.37 to 6.7)	7.97 (6.03 to 10.39)	-28.54 (-39.16 to -15.34)	-1.06 (-1.15 to -0.98)
**European Region**							

Ischaemic heart disease	325336.67 (268095.67 to 383711.21)	-33.18 (-37.41 to -29.59)	34.85 (28.72 to 41.11)	-38.56 (-42.44 to -35.26)	20.01 (16.61 to 23.45)	-56.85 (-59.48 to -54.42)	-3.03 (-3.15 to -2.91)
Ischemic stroke	69370.38 (54643.67 to 86494.7)	-41.62 (-45.2 to -38.3)	7.43 (5.85 to 9.27)	-46.32 (-49.61 to -43.26)	3.97 (3.17 to 4.89)	-64.49 (-66.6 to -62.2)	-3.45 (-3.7 to -3.19)
**Region of the Americas**							
Ischaemic heart disease	159509.59 (130660.14 to 191275.28)	-27.26 (-31.39 to -22.93)	15.53 (12.72 to 18.63)	-49.27 (-52.15 to -46.25)	11.9 (9.78 to 14.24)	-67.4 (-69.16 to -65.49)	-3.82 (-3.88 to -3.75)
Ischemic stroke	22010.74 (16807.76 to 28270.79)	-23.8 (-29.51 to -17.14)	2.14 (1.64 to 2.75)	-46.86 (-50.84 to -42.22)	1.6 (1.22 to 2.05)	-67.02 (-69.37 to -64.29)	-4.33 (-4.49 to -4.16)
**South-East Asia Region**							
Ischaemic heart disease	411943.31 (338245.37 to 490098.68)	109.5 (83.24 to 138.72)	19.96 (16.39 to 23.75)	32.3 (15.72 to 50.76)	23.34 (19.08 to 27.96)	-16.73 (-26.87 to -5.08)	-0.6 (-0.71 to -0.49)
Ischemic stroke	79338.05 (59496.57 to 101667.98)	108.47 (77.51 to 149.62)	3.84 (2.88 to 4.93)	31.65 (12.1 to 57.64)	5.14 (3.82 to 6.61)	-28.27 (-38.18 to -14.9)	-1.24 (-1.33 to -1.16)
**Western Pacific Region**							
Ischaemic heart disease	576226.24 (446368.49 to 716564.97)	143.75 (99.89 to 198.17)	29.93 (23.18 to 37.22)	95.16 (60.05 to 138.74)	21.02 (16.23 to 26.15)	-9.62 (-25.25 to 10.19)	-0.08 (-0.18 to 0.02)
Ischemic stroke	239851.55 (180059.97 to 312459.66)	99.41 (55.26 to 155.4)	12.46 (9.35 to 16.23)	59.66 (24.31 to 104.49)	8.59 (6.46 to 11.2)	-32.54 (-46.89 to -14.76)	-1.23 (-1.42 to -1.04)
**Low SDI**							

Ischaemic heart disease	69614.07 (56413.7 to 83883.85)	85.49 (62.07 to 108.98)	6.23 (5.05 to 7.51)	-16.78 (-27.29 to -6.24)	14.23 (11.38 to 17.37)	-16.25 (-26.83 to -5.55)	-0.65 (-0.77 to -0.52)
Ischemic stroke	13370.73 (9913 to 17766.41)	71.93 (47.45 to 100.36)	1.2 (0.89 to 1.59)	-22.86 (-33.85 to -10.11)	3.33 (2.46 to 4.43)	-26.01 (-36.39 to -14.53)	-1.11 (-1.25 to -0.97)
**Low-middle SDI**							
Ischaemic heart disease							
Ischemic stroke	69301.23 (52141.43 to 88837.42)	85.72 (61.81 to 116.21)	3.61 (2.71 to 4.62)	12.28 (-2.18 to 30.71)	5.61 (4.22 to 7.26)	-26.89 (-35.88 to -15.38)	-1.05 (-1.13 to -0.98)
**Middle SDI**							
Ischaemic heart disease	561343.16 (457365.99 to 666856.58)	107.81 (84.29 to 133.45)	22.93 (18.68 to 27.23)	46.23 (29.68 to 64.27)	22.2 (17.76 to 26.76)	-21.82 (-30.18 to -12.78)	-0.85 (-0.91 to -0.78)
Ischemic stroke	173811.11 (132341.63 to 221678.01)	97 (60.51 to 142.26)	7.1 (5.4 to 9.05)	38.62 (12.94 to 70.47)	7.17 (5.42 to 9.17)	-32.28 (-44.09 to -17.57)	-1.4 (-1.53 to -1.28)
**High-middle SDI**							
Ischaemic heart disease	561343.16 (457365.99 to 666856.58)	107.81 (84.29 to 133.45)	22.93 (18.68 to 27.23)	46.23 (29.68 to 64.27)	22.2 (17.76 to 26.76)	-21.82 (-30.18 to -12.78)	-0.85 (-0.91 to -0.78)
Ischemic stroke	173811.11 (132341.63 to 221678.01)	97 (60.51 to 142.26)	7.1 (5.4 to 9.05)	38.62 (12.94 to 70.47)	7.17 (5.42 to 9.17)	-32.28 (-44.09 to -17.57)	-1.4 (-1.53 to -1.28)
**High SDI**							

Ischaemic heart disease	214236.69 (173516.39 to 257782.3)	-46.48 (-49.8 to -43.35)	19.58 (15.86 to 23.56)	-56.97 (-59.64 to -54.46)	10.4 (8.61 to 12.38)	-71.54 (-72.85 to -70.09)	-4.07 (-4.16 to -3.97)
Ischemic stroke	42650.32 (32222.65 to 54442.32)	-42.97 (-48.19 to -37.95)	3.9 (2.95 to 4.98)	-54.15 (-58.35 to -50.12)	1.82 (1.4 to 2.3)	-72.67 (-74.65 to -70.45)	-4.08 (-4.18 to -3.97)

*The all-age mortality is equivalent to the crude mortality rate.

*The numbers in parentheses in the table represent the 95% confidence interval.

Abbreviation: CVD, cardiovascular disease. SDI, socio-demographic inde

Table S2. The global trends in the male CVD burden attributable to tobacco by SDI and region from 1990 to 2021

Characteristics	Deaths		All-age mortality		Age-standardized mortality		Net drift of mortality, % per year
	Number in 2021, n	Change of numbers 1990-2021, %	Rate in 2021, per 100 000	Percent change 1990-2021, %	Rate in 2021, per 100 000	Percent change 1990-2021, %	
**Global**							
CVD	43824585.02 (37124273.52 to 51000509.18)	78.69 (47.63 to 116.34)	1106.85 (937.63 to 1288.09)	-10.96 (-32.03 to 14.58)	1068.33 (901.5 to 1246.55)	-73.21 (-86.55 to -56.87)	-1.54 (-1.62 to -1.46)
Ischaemic heart disease	35418596.12 (30305596.22 to 40736196.49)	32.98 (22.62 to 45.84)	894.55 (765.41 to 1028.85)	-9.8 (-16.82 to -1.07)	858.16 (731.51 to 989.95)	-37.12 (-41.85 to -31.09)	-1.54 (-1.63 to -1.45)
Ischemic stroke	8405988.9 (6818677.31 to 10264312.69)	45.71 (25.01 to 70.5)	212.31 (172.22 to 259.24)	-1.16 (-15.2 to 15.65)	210.17 (169.99 to 256.61)	-36.08 (-44.7 to -25.78)	-1.51 (-1.58 to -1.44)
**African Region**							
CVD	1356069.56 (1091934.49 to 1639706.17)	157.39 (110.86 to 209.93)	238.3 (191.88 to 288.14)	-41.57 (-62.2 to -18.28)	510.78 (409.61 to 621.6)	-41.52 (-60.9 to -18.72)	-0.98 (-1.1 to -0.87)
Ischaemic heart disease	1095033.96 (891548.16 to 1310177.05)	82.44 (61.16 to 103.71)	192.43 (156.67 to 230.23)	-19.13 (-28.56 to -9.7)	398.96 (323.85 to 477.53)	-19.22 (-27.9 to -10.11)	-0.96 (-1.09 to -0.82)
Ischemic stroke	261035.6 (200386.32 to 329529.12)	74.95 (49.7 to 106.23)	45.87 (35.21 to 57.91)	-22.45 (-33.64 to -8.58)	111.82 (85.76 to 144.07)	-22.3 (-32.99 to -8.61)	-0.94 (-1.04 to -0.84)
**Eastern Mediterranean Region**							

CVD	4859785.7 (3975542.24 to 5771865.41)	197.44 (134.31 to 272.85)	1242.95 (1016.79 to 1476.22)	-2.47 (-33.85 to 35.01)	1791.1 (1449.55 to 2131.8)	-45.45 (-69.47 to -16.33)	-0.81 (-0.87 to -0.75)
Ischaemic heart disease	4243201.41 (3484574.72 to 5004185.4)	101.28 (73.49 to 135.4)	1085.25 (891.22 to 1279.88)	0.04 (-13.77 to 17)	1530.97 (1241.38 to 1806.77)	-22.88 (-33.23 to -9.62)	-0.78 (-0.85 to -0.72)
Ischemic stroke	616584.29 (490967.52 to 767680.01)	96.16 (60.82 to 137.45)	157.7 (125.57 to 196.34)	-2.51 (-20.07 to 18.01)	260.14 (208.18 to 325.03)	-22.57 (-36.24 to -6.71)	-0.75 (-0.82 to -0.68)
**European Region**							
CVD	7712399.73 (6587462.11 to 8936351.34)	-66.81 (-75.96 to -57.99)	1698.35 (1450.62 to 1967.87)	-78.23 (-86.6 to -70.17)	1138.9 (974.38 to 1313.79)	-115.42 (-121.34 to -109.43)	-3.19 (-3.3 to -3.08)
Ischaemic heart disease	6477581.28 (5559045.87 to 7463442.18)	-34.1 (-38.87 to -29.63)	1426.43 (1224.16 to 1643.52)	-39.75 (-44.12 to -35.66)	962.38 (826.58 to 1103.93)	-56.47 (-59.56 to -53.54)	-3.13 (-3.24 to -3.02)
Ischemic stroke	1234818.45 (1028416.24 to 1472909.16)	-32.71 (-37.09 to -28.36)	271.92 (226.47 to 324.35)	-38.48 (-42.48 to -34.51)	176.52 (147.79 to 209.86)	-58.96 (-61.78 to -55.89)	-3.2 (-3.32 to -3.09)
**Region of the Americas**							
CVD	3371413.87 (2827350.03 to 3937226.53)	-44.49 (-53.37 to -34.49)	669.35 (561.33 to 781.69)	-91.24 (-97.45 to -84.24)	555.97 (466.43 to 649.49)	-129.67 (-133.62 to -125.18)	-3.69 (-3.75 to -3.62)
Ischaemic heart disease	3025200.53 (2545879.75 to 3509020.31)	-24.17 (-28.26 to -19.73)	600.61 (505.45 to 696.67)	-46.97 (-49.83 to -43.86)	499.12 (420.26 to 578.97)	-65.02 (-66.8 to -62.94)	-3.71 (-3.78 to -3.64)
Ischemic stroke	346213.34 (281470.27 to 428206.23)	-20.32 (-25.11 to -14.76)	68.74 (55.88 to 85.01)	-44.27 (-47.62 to -40.38)	56.85 (46.17 to 70.51)	-64.65 (-66.82 to -62.24)	-3.71 (-3.77 to -3.66)

**South-East Asia Region**							
CVD	11390133.55 (9387382.68 to 13601750.65)	195.94 (132.6 to 269.57)	1089.01 (897.53 to 1300.46)	53.35 (12.82 to 100.46)	1223.46 (1003.14 to 1465.87)	-34.23 (-59.15 to -3.77)	-0.4 (-0.57 to -0.23)
Ischaemic heart disease	9937431.7 (8288014.98 to 11765517.77)	95.41 (68.95 to 124.81)	950.12 (792.42 to 1124.9)	25.03 (8.11 to 43.85)	1049.62 (871.11 to 1245.11)	-13.28 (-24.67 to -0.03)	-0.27 (-0.47 to -0.06)
Ischemic stroke	1452701.85 (1099367.7 to 1836232.88)	100.53 (63.64 to 144.76)	138.89 (105.11 to 175.56)	28.31 (4.71 to 56.61)	173.84 (132.03 to 220.76)	-20.95 (-34.48 to -3.74)	-0.82 (-0.9 to -0.75)
**Western Pacific Region**							
CVD	14999937.74 (11707550.61 to 18933126.54)	210.87 (113.63 to 332.44)	1532.12 (1195.83 to 1933.87)	130.49 (52.28 to 228.28)	1148.4 (896.66 to 1441.05)	-29.42 (-68.63 to 18.48)	-0.01 (-0.14 to 0.11)
Ischaemic heart disease	10530876.8 (8256010.55 to 13161469.3)	114.62 (66.48 to 172.86)	1075.64 (843.28 to 1344.34)	72.64 (33.91 to 119.48)	811.18 (639.2 to 1006.49)	-4.82 (-25.51 to 20.77)	0.32 (0.18 to 0.46)
Ischemic stroke	4469060.95 (3451540.06 to 5771657.24)	96.24 (47.15 to 159.58)	456.48 (352.55 to 589.53)	57.85 (18.36 to 108.8)	337.22 (257.46 to 434.56)	-24.6 (-43.12 to -2.29)	-0.68 (-0.81 to -0.54)
**Low SDI**							
CVD	1953247.27 (1574580.31 to 2367421.81)	167.26 (108.68 to 230.19)	349.35 (281.62 to 423.43)	-34.25 (-60.69 to -5.85)	699.68 (563.78 to 852.68)	-33.63 (-59.53 to -5.93)	-0.53 (-0.72 to -0.35)
Ischaemic heart disease	1695729.03 (1380348.86 to 2025651.19)	89.85 (60.19 to 118.13)	303.29 (246.88 to 362.3)	-14.32 (-27.7 to -1.55)	592.89 (483.21 to 710.12)	-13.43 (-26.18 to -1)	-0.4 (-0.63 to -0.18)
Ischemic stroke	257518.23 (194231.45 to 341770.62)	77.42 (48.49 to 112.06)	46.06 (34.74 to 61.13)	-19.93 (-32.99 to -4.3)	106.79 (80.57 to 142.57)	-20.2 (-33.34 to -4.93)	-0.85 (-0.93 to -0.76)
**Low-middle SDI**							

CVD	9970398.8 (8328601.24 to 11670439.49)	184.19 (130.22 to 244.8)	1032.42 (862.42 to 1208.46)	35.22 (2.17 to 72.32)	1340.3 (1112.56 to 1579.25)	-25.79 (-49.45 to 1.37)	-0.17 (-0.29 to -0.05)
Ischaemic heart disease	8675891.31 (7323024.22 to 10008534.69)	97.57 (72.63 to 123.11)	898.38 (758.29 to 1036.37)	20.96 (5.69 to 36.59)	1146.01 (961.57 to 1329.97)	-7.92 (-19.42 to 3.88)	-0.04 (-0.17 to 0.1)
Ischemic stroke	1294507.5 (1005577.02 to 1661904.8)	86.63 (57.59 to 121.69)	134.04 (104.13 to 172.09)	14.26 (-3.52 to 35.73)	194.3 (150.99 to 249.28)	-17.87 (-30.03 to -2.51)	-0.61 (-0.68 to -0.53)
**Middle SDI**							
CVD	15189147.08 (12542085.91 to 17948952.98)	188.73 (121.51 to 267.29)	1231.69 (1017.04 to 1455.48)	76.47 (28.66 to 132.34)	1175.38 (962.88 to 1395.47)	-42.74 (-68.64 to -11.82)	-0.58 (-0.68 to -0.49)
Ischaemic heart disease	11882189.55 (9955958.91 to 13872432.81)	90.87 (66.99 to 118.99)	963.53 (807.33 to 1124.91)	35.75 (18.76 to 55.75)	907.73 (754.94 to 1062.05)	-18.62 (-28.6 to -6.38)	-0.49 (-0.59 to -0.39)
Ischemic stroke	3306957.53 (2586127 to 4076520.17)	97.86 (54.53 to 148.3)	268.16 (209.71 to 330.56)	40.72 (9.9 to 76.59)	267.65 (207.94 to 333.43)	-24.12 (-40.04 to -5.44)	-0.79 (-0.88 to -0.69)
**High-middle SDI**							
CVD	12056412.56 (10083712.74 to 14235875.57)	46.6 (12.49 to 85.15)	1847.55 (1545.25 to 2181.54)	0.01 (-27.66 to 31.27)	1355.46 (1128.84 to 1605.68)	-79.16 (-95.41 to -60.47)	-1.82 (-1.92 to -1.72)
Ischaemic heart disease	9260809.99 (7827385.23 to 10791631.28)	16.65 (3.24 to 31.21)	1419.15 (1199.49 to 1653.74)	-5.39 (-16.27 to 6.42)	1040.63 (877.01 to 1214.52)	-40.09 (-46.78 to -32.54)	-1.77 (-1.88 to -1.67)
Ischemic stroke	2795602.56 (2256327.52 to 3444244.29)	29.95 (9.25 to 53.94)	428.4 (345.77 to 527.8)	5.4 (-11.39 to 24.85)	314.83 (251.83 to 391.15)	-39.06 (-48.62 to -27.94)	-1.79 (-1.9 to -1.68)
**High SDI**							

CVD	4616878.5 (3877676.5 to 5415162.63)	-78.01 (-86.6 to -69.15)	846.06 (710.6 to 992.35)	-103.12 (-109.94 to -96.08)	534.32 (451.33 to 622.19)	-135.79 (-140.18 to -130.66)	-4 (-4.07 to -3.93)
Ischaemic heart disease	3871790.09 (3279757.92 to 4505048.73)	-45.78 (-48.77 to -42.87)	709.52 (601.03 to 825.57)	-56.94 (-59.32 to -54.62)	452.8 (385.56 to 522.89)	-69.86 (-71.39 to -68.01)	-4.08 (-4.16 to -4.01)
Ischemic stroke	745088.41 (597918.58 to 910113.91)	-32.24 (-37.83 to -26.28)	136.54 (109.57 to 166.78)	-46.18 (-50.62 to -41.45)	81.53 (65.77 to 99.3)	-65.93 (-68.79 to -62.65)	-3.43 (-3.49 to -3.36)

*The all-age mortality is equivalent to the crude mortality rate.

*The numbers in parentheses in the table represent the 95% confidence interval.

Abbreviation: CVD, cardiovascular disease. SDI, socio-demographic index

Table S3. The global trends in the female CVD burden attributable to tobacco by SDI and region from 1990 to 2021

Characteristics	Deaths		All-age mortality		Age-standardized mortality		Net drift of mortality, % per year
	Number in 2021, n	Change of numbers 1990-2021, %	Rate in 2021, per 100 000	Percent change 1990-2021, %	Rate in 2021, per 100 000	Percent change 1990-2021, %	
**Global**							
CVD	10321102.08 (8031115.32 to 12919268.99)	11.39 (-10.5 to 33.59)	262.49 (204.25 to 328.57)	-57.65 (-72.39 to -42.69)	225.84 (175.95 to 282.36)	-103.12 (-112.85 to -92.85)	-2.47 (-2.51 to -2.43)
Ischaemic heart disease	7808383.36 (6178914.33 to 9648893.87)	4.23 (-6.5 to 14.41)	198.59 (157.15 to 245.4)	-29.81 (-37.04 to -22.95)	171.25 (135.68 to 211.36)	-51.6 (-56.34 to -46.84)	-2.43 (-2.48 to -2.39)
Ischemic stroke	2512718.72 (1852201 to 3270375.12)	7.16 (-4 to 19.18)	63.9 (47.11 to 83.17)	-27.84 (-35.35 to -19.74)	54.59 (40.27 to 70.99)	-51.52 (-56.51 to -46.01)	-2.54 (-2.58 to -2.5)
**African Region**							
CVD	313863.74 (236003.58 to 401015.98)	107.48 (77.16 to 144.03)	53.52 (40.24 to 68.38)	-65.21 (-78.5 to -49.19)	117.2 (87.25 to 151.28)	-69.93 (-83.23 to -54.09)	-1.68 (-1.83 to -1.54)
Ischaemic heart disease	225032.57 (173467.45 to 282689.92)	56.15 (39.08 to 75.15)	38.37 (29.58 to 48.21)	-31.55 (-39.03 to -23.22)	81.15 (61.99 to 102.94)	-34.06 (-41.74 to -26.18)	-1.66 (-1.84 to -1.49)
Ischemic stroke	88831.17 (62536.13 to 118326.06)	51.34 (38.08 to 68.88)	15.15 (10.66 to 20.18)	-33.66 (-39.47 to -25.97)	36.05 (25.26 to 48.35)	-35.87 (-41.49 to -27.91)	-1.7 (-1.8 to -1.6)
**Eastern Mediterranean Region**							

CVD	1050808.33 (769056.01 to 1361526.8)	122.91 (74.54 to 178.47)	290.25 (212.42 to 376.07)	-38.2 (-62.44 to -10.37)	454.2 (331.74 to 591.58)	-74.91 (-92.9 to -54.25)	-1.74 (-1.81 to -1.68)
Ischaemic heart disease	862845.86 (638196.71 to 1106839.26)	56.81 (32.27 to 81.6)	238.33 (176.28 to 305.72)	-21.43 (-33.72 to -9.01)	366.13 (271.04 to 471.74)	-39.05 (-48.19 to -30.18)	-1.76 (-1.82 to -1.69)
Ischemic stroke	187962.47 (130859.3 to 254687.54)	66.1 (42.27 to 96.86)	51.92 (36.15 to 70.35)	-16.77 (-28.72 to -1.36)	88.07 (60.7 to 119.85)	-35.86 (-44.7 to -24.07)	-1.6 (-1.67 to -1.52)
**European Region**							
CVD	1770546.23 (1401251.14 to 2196137.99)	-93.9 (-102.02 to -86.67)	369.39 (292.34 to 458.18)	-101.91 (-109.42 to -95.23)	201.06 (162.32 to 246.53)	-124.45 (-130.55 to -118.56)	-3.26 (-3.36 to -3.15)
Ischaemic heart disease	1347029.02 (1080680.51 to 1653219.85)	-45.26 (-49.71 to -41.28)	281.03 (225.46 to 344.91)	-49.4 (-53.51 to -45.72)	153.84 (125.72 to 186.67)	-61.16 (-64.27 to -58.05)	-3.22 (-3.34 to -3.11)
Ischemic stroke	423517.21 (320570.63 to 542918.14)	-48.63 (-52.31 to -45.39)	88.36 (66.88 to 113.27)	-52.51 (-55.91 to -49.51)	47.22 (36.59 to 59.86)	-63.29 (-66.28 to -60.51)	-3.32 (-3.44 to -3.21)
**Region of the Americas**							
CVD	1221245.96 (986701.37 to 1488132.79)	-54.98 (-65.5 to -43.23)	233.42 (188.59 to 284.44)	-99.14 (-106.45 to -90.96)	172.03 (139.83 to 208.66)	-131.81 (-136.67 to -126.58)	-4.01 (-4.05 to -3.96)
Ischaemic heart disease	1007895.01 (821125.41 to 1213142.95)	-33.25 (-37.46 to -28.47)	192.64 (156.95 to 231.88)	-53.58 (-56.5 to -50.25)	142.76 (116.84 to 171.1)	-68.53 (-70.46 to -66.44)	-4.1 (-4.14 to -4.05)

Ischemic stroke	213350.95 (165575.96 to 274989.83)	-21.73 (-28.04 to -14.76)	40.78 (31.65 to 52.56)	-45.56 (-49.95 to -40.71)	29.27 (23 to 37.55)	-63.27 (-66.2 to -60.14)	-3.67 (-3.73 to -3.61)
**South-East Asia Region**							
CVD	2305317.18 (1742744.8 to 2955860.92)	129.47 (80.26 to 193.63)	226.52 (171.24 to 290.45)	5.24 (-25.42 to 45.21)	242.15 (183.32 to 310.92)	-76.36 (-94.26 to -52.85)	-1.47 (-1.59 to -1.36)
Ischaemic heart disease	1833917.44 (1409912.87 to 2329663.52)	59.79 (33.39 to 93.02)	180.2 (138.54 to 228.92)	-0.46 (-16.91 to 20.24)	189.54 (146.07 to 240.52)	-36.65 (-46.44 to -24.02)	-1.33 (-1.47 to -1.2)
Ischemic stroke	471399.74 (332831.93 to 626197.4)	69.68 (46.87 to 100.62)	46.32 (32.7 to 61.53)	5.7 (-8.51 to 24.97)	52.61 (37.25 to 70.4)	-39.71 (-47.82 to -28.83)	-1.78 (-1.84 to -1.71)
**Western Pacific Region**							
CVD	1119949.9 (786729.24 to 1534203.86)	42.21 (11.71 to 79.45)	118.36 (83.14 to 162.13)	13.32 (-10.98 to 42.99)	74.32 (52.37 to 101.75)	-46.1 (-57.36 to -32)	-2.13 (-2.23 to -2.04)
Ischaemic heart disease	2512376.11 (1827746.65 to 3334414.41)	52.21 (14.48 to 98.91)	265.51 (193.16 to 352.38)	21.28 (-8.77 to 58.5)	169.39 (124.01 to 225.34)	-39.01 (-54.08 to -21.62)	-1.48 (-1.57 to -1.4)
Ischemic stroke	1119949.9 (786729.24 to 1534203.86)	42.21 (11.71 to 79.45)	118.36 (83.14 to 162.13)	13.32 (-10.98 to 42.99)	74.32 (52.37 to 101.75)	-46.1 (-57.36 to -32)	-2.13 (-2.23 to -2.04)
**Low SDI**							
CVD	490163.44 (363579.29 to 649730.72)	103.34 (59.3 to 157.5)	87.8 (65.13 to 116.38)	-64.72 (-84.36 to -40.57)	182.05 (134.82 to 241.32)	-69.33 (-87.19 to -46.27)	-1.42 (-1.56 to -1.29)
Ischaemic heart disease	391165.63 (294302.04 to 511605.21)	49.3 (25.41 to 74.98)	70.07 (52.72 to 91.64)	-33.42 (-44.07 to -21.97)	141.49 (106.51 to 184.07)	-34.51 (-44.41 to -23.64)	-1.31 (-1.48 to -1.14)
Ischemic stroke	98997.81 (69277.25 to 138125.51)	54.04 (33.89 to 82.52)	17.73 (12.41 to 24.74)	-31.31 (-40.29 to -18.6)	40.56 (28.31 to 57.24)	-34.82 (-42.78 to -22.63)	-1.58 (-1.65 to -1.51)

**Low-middle SDI**							
CVD	2094545.95 (1597266.54 to 2641570.45)	107.11 (67.96 to 155.74)	219.24 (167.19 to 276.49)	-16.72 (-40.09 to 12.3)	273 (207.87 to 345.26)	-75.52 (-90.95 to -56.76)	-1.46 (-1.54 to -1.38)
Ischaemic heart disease	1694160.16 (1313051.38 to 2111164.48)	52.16 (32.77 to 75.94)	177.33 (137.44 to 220.98)	-9.2 (-20.76 to 5)	217.22 (168.37 to 271.43)	-36.1 (-44.13 to -26.74)	-1.36 (-1.45 to -1.27)
Ischemic stroke	400385.8 (284215.16 to 530405.96)	54.95 (35.18 to 79.8)	41.91 (29.75 to 55.52)	-7.53 (-19.32 to 7.3)	55.78 (39.5 to 73.83)	-39.42 (-46.82 to -30.02)	-1.71 (-1.77 to -1.66)
**Middle SDI**							
CVD	3315468.63 (2523758.11 to 4278407.6)	86.51 (44.7 to 132.14)	272.8 (207.66 to 352.03)	-0.6 (-29.7 to 31.16)	238.37 (181.13 to 308.13)	-92.26 (-107.73 to -74.83)	-2.08 (-2.13 to -2.03)
Ischaemic heart disease	2457706.91 (1913316.5 to 3105801.17)	43.69 (23.45 to 65.81)	202.22 (157.43 to 255.55)	0.01 (-14.08 to 15.4)	175.98 (136.84 to 222.93)	-44.15 (-51.92 to -35.49)	-1.94 (-2 to -1.89)
Ischemic stroke	857761.72 (610441.61 to 1172606.44)	42.82 (21.25 to 66.32)	70.58 (50.23 to 96.48)	-0.6 (-15.62 to 15.76)	62.39 (44.29 to 85.2)	-48.11 (-55.81 to -39.35)	-2.34 (-2.4 to -2.28)
**High-middle SDI**							
CVD	3016756.36 (2316839.44 to 3829467.98)	5.34 (-23.93 to 38.9)	463.07 (355.63 to 587.82)	-31.6 (-55.6 to -4.07)	280.14 (215.62 to 354.64)	-96.2 (-110.73 to -79.89)	-2.26 (-2.33 to -2.19)
Ischaemic heart disease	2185477.9 (1707252.06 to 2737918.62)	7.37 (-8.23 to 25.98)	335.47 (262.06 to 420.27)	-11.95 (-24.74 to 3.32)	203.58 (159.41 to 254.19)	-45.54 (-53.21 to -36.36)	-2.13 (-2.2 to -2.05)
Ischemic stroke	831278.46 (609587.38 to 1091549.36)	-2.02 (-15.7 to 12.92)	127.6 (93.57 to 167.55)	-19.65 (-30.86 to -7.39)	76.57 (56.21 to 100.44)	-50.66 (-57.52 to -43.52)	-2.53 (-2.61 to -2.45)

**High SDI**							
CVD	1392727.43 (1113984.43 to 1718552.13)	-98.07 (-105.74 to -89.9)	253.98 (203.15 to 313.4)	-117.07 (-123.31 to -110.43)	133.38 (109.33 to 161.98)	-139.07 (-143.48 to -134.3)	-4.17 (-4.24 to -4.11)
Ischaemic heart disease	1071178.18 (866592.45 to 1299923.47)	-54.56 (-57.83 to -51.36)	195.34 (158.03 to 237.06)	-63.03 (-65.69 to -60.43)	103.9 (86.21 to 124)	-72.68 (-74.21 to -70.88)	-4.32 (-4.39 to -4.25)
Ischemic stroke	321549.25 (247391.98 to 418628.67)	-43.52 (-47.91 to -38.54)	58.64 (45.12 to 76.34)	-54.05 (-57.62 to -50)	29.47 (23.12 to 37.98)	-66.39 (-69.27 to -63.42)	-3.6 (-3.66 to -3.53)

*The all-age mortality is equivalent to the crude mortality rate.

*The numbers in parentheses in the table represent the 95% confidence interval.

Abbreviation: CVD, cardiovascular disease. SDI, socio-demographic index

Table S4. The global trends in CVD burden attributable to tobacco by different countries from 1990 to 2021.

	Deaths 1990	ASMR 1990	DALYs 1990	ASDR 1990	Deaths 2021	ASMR 2021	DALYs 2021	ASDR 2021	EAPC ASMR	EAPC ASDR
Afghanistan	3685.9 (2485.6-5143.1)	55.3 (37.7-77.2)	104847.3 (70984-144553.5)	1442.8 (975.5-1987.2)	4584.5 (3082.8-6537.4)	45 (30.1-63.6)	153123.6 (102286.9-218596.3)	1180.6 (802.8-1662.7)	-0.64 (-0.87--0.41)	-0.67 (-0.89--0.44)
Albania	1074.1 (851.7-1292.3)	60 (47.1-73.3)	25072.6 (20296.7-29502.2)	1238.2 (996.4-1473)	2047.8 (1524.6-2596.6)	49.3 (36.6-62.4)	39382.9 (30170.3-49009.4)	939.1 (720.9-1163.9)	-0.27 (-0.5--0.04)	-0.53 (-0.75--0.32)
Algeria	8004.8 (6162-10239.2)	90.9 (68.4-117.2)	211436.3 (165019.7-269607.1)	1828.4 (1420-2322.2)	14227.2 (10176.8-19173.6)	52.5 (37.4-70.5)	342676.1 (250990.8-451861.3)	1002.1 (729.5-1336.4)	-1.84 (-1.98--1.69)	-2.18 (-2.32--2.04)
American Samoa	9.7 (7.7-12.2)	41.2 (32.2-52.5)	339.1 (269.7-421)	1213.6 (961.5-1514)	19.5 (15-25.3)	39.9 (30.4-51.7)	633.7 (488.8-814.8)	1198.2 (915.8-1549.6)	-0.11 (-0.21--0.01)	-0.03 (-0.13-0.08)
Andorra	11.1 (7.6-15.6)	20.4 (13.9-28.9)	293.7 (208.9-407.3)	504.5 (357.3-698.6)	12 (7.8-16.9)	7.4 (4.8-10.5)	284 (190.7-389.1)	186.7 (125.3-255.3)	-3.13 (-3.39--2.86)	-3.1 (-3.32--2.87)
Angola	739.8 (534.8-997.8)	19.5 (14.2-26)	23445.8 (16784.5-31411.6)	522.1 (380.1-698.7)	1961.2 (1356.2-2674.5)	17.1 (12-23.2)	62302 (43492.2-84993.3)	447.6 (312.6-605.7)	-0.48 (-0.72--0.25)	-0.55 (-0.79--0.31)
Antigua and Barbuda	9.8 (7.7-12.5)	18.3 (14.4-23)	232.2 (185.7-286.9)	457.5 (368.5-564.1)	8.9 (6.8-11.6)	8.8 (6.6-11.5)	218.2 (168.4-279.3)	200 (154.3-256.2)	-2.79 (-3.06--2.52)	-3.05 (-3.31--2.8)
Argentina	11089.2 (8996.4-	35.2 (28.3-	302140.5 (252773.2-	936.4 (782.3-	6194.6 (4969.1-	11.2 (9-13.8)	166164.4 (137105-	310.6 (256.7-	-3.36 (-3.46--	-3.32 (-3.41--

	13357.4)	42.9)	357324.5)	1107.4)	7633.4)		200030.8)	372.3)	3.26)	3.24)
Armenia	2151.2 (1747.5-2560.7)	85.5 (67.6-103.3)	55582.7 (47092.8-64722.9)	1989.6 (1662.2-2335.1)	2642.9 (2121.1-3210.9)	61.3 (49-74.4)	59542.5 (48957.5-71075)	1405.7 (1157.8-1677)	-1.67 (-1.83--1.5)	-1.63 (-1.8--1.47)
Australia	6415.7 (5167.7-7845.9)	33 (26.7-40.4)	155427.9 (127778.2-184904)	805.9 (662.6-956.7)	2334.6 (1780.3-2992.2)	5.2 (4.1-6.5)	56069.1 (44992-69402.6)	143.4 (117.1-175.1)	-6.07 (-6.18--5.96)	-5.62 (-5.75--5.48)
Austria	3906.1 (3189.8-4700.7)	33.5 (27.7-40)	91507.5 (77466-107061.8)	840.2 (716.7-978.9)	2291.6 (1786.6-2849.7)	12.1 (9.7-14.7)	50410.6 (41070.2-60559.9)	303.7 (250.4-360.1)	-3.62 (-3.86--3.39)	-3.63 (-3.87--3.39)
Azerbaijan	4124.4 (3318.2-5001.2)	86.5 (68.7-106)	115419.1 (94329.8-136864.4)	2188 (1780.7-2613.5)	6729.9 (5299.5-8257.7)	76.1 (59.6-95.3)	170531.1 (135216.6-207179.6)	1650.1 (1307.8-2004.9)	-0.24 (-0.53-0.06)	-0.99 (-1.29--0.68)
Bahamas	29.6 (23.1-37.9)	19 (14.8-24.2)	891.3 (697.9-1109.5)	524.9 (410.5-660.8)	42.7 (29.9-57.1)	10.5 (7.3-14.1)	1244.3 (881.3-1642.1)	283 (200-373.2)	-1.91 (-2.07--1.75)	-1.95 (-2.12--1.77)
Bahrain	141 (110.9-174.7)	94.1 (71.9-119.8)	4353.3 (3448.1-5303.8)	2116.9 (1665.5-2646.7)	236.8 (182.6-305.6)	33.8 (24.8-44.1)	7603.5 (5871.8-9752.8)	710 (544.7-912.9)	-3.99 (-4.34--3.63)	-4.29 (-4.6--3.97)
Bangladesh	19049.5 (14693.6-24448)	41.6 (31.8-53.6)	558957.3 (439608.4-713381.2)	1090.9 (852.2-1394.5)	42813.7 (30260.4-57624.4)	33.4 (23.6-44.8)	1128790.8 (804875.1-1508274.6)	796.6 (566.5-1062.1)	-0.6 (-0.75--0.46)	-0.8 (-0.92--0.68)
Barbados	43.7 (33.1-55.6)	14.8 (11.3-18.7)	949.2 (744.6-1187.1)	347.4 (275.6-430.3)	31.6 (21.3-43.7)	6.1 (4.1-8.4)	685.8 (475.3-942.9)	138.4 (96.2-189.4)	-3.45 (-3.77--3.13)	-3.48 (-3.77--3.2)
Belarus	9579.9	76.1	245471.7	1935.8	12392.3	77.1	297605.5	1918.9	-0.35 (-	-0.57 (-

	(7777.4-11662.3)	(61.7-92.8)	(204464.5-290011.8)	(1611.9-2294)	(9578.6-15435.9)	(59.8-95.5)	(233050.1-366772.5)	(1517.9-2357.3)	0.73-0.03)	1.02--0.11)
Belgium	6174.5 (4987.4-7398.3)	40.2 (32.8-48)	136741.5 (114761.2-159729.4)	939.6 (795-1093.5)	1978.2 (1537.7-2489.2)	8.1 (6.5-9.9)	42782.9 (34726-51662.4)	204.9 (169.4-243.8)	-5.19 (-5.34--5.04)	-4.95 (-5.08--4.82)
Belize	17.2 (13.8-21)	18.6 (14.9-22.8)	453.6 (372.6-544.9)	478.8 (392.6-575.6)	27 (20.6-34.6)	9.3 (7-11.9)	760 (596.4-962)	234.8 (182.2-298.7)	-2.69 (-3.01--2.37)	-2.71 (-3--2.43)
Benin	191.9 (141.4-256.5)	10.1 (7.4-13.5)	5224.6 (3990-6896.4)	254.2 (191.3-336.2)	293.1 (200.1-407.4)	6 (4.1-8.4)	8575.6 (5922.9-11717.6)	152.3 (105.4-209.7)	-1.87 (-2.02--1.72)	-1.86 (-2.02--1.7)
Bermuda	21.9 (17-27.4)	36.1 (27.8-45.3)	559.2 (442.8-683.7)	880.4 (693.9-1080.7)	15.1 (11.1-20.5)	10.9 (8-14.6)	334.8 (252.5-445.7)	267.7 (203-352.7)	-3.77 (-4.14--3.41)	-3.73 (-4.13--3.34)
Bhutan	42.7 (25.9-64.4)	18.7 (11.3-28.4)	1352.9 (815.9-2007.7)	484 (298.7-723.5)	95.2 (62.5-143.1)	16.4 (10.8-24.8)	2417.9 (1554.1-3607.6)	382.1 (248.7-568.4)	-0.41 (-0.47--0.35)	-0.8 (-0.86--0.75)
Bolivia (Plurinational State of)	559.9 (386.9-758.6)	18.4 (12.8-25)	15857.4 (10978-21494.6)	462.8 (322.7-627.3)	747.1 (475.3-1090.5)	8.7 (5.6-12.6)	20182.8 (13032.1-29627.4)	212.1 (137.7-309.3)	-2.16 (-2.53--1.79)	-2.3 (-2.69--1.92)
Bosnia and Herzegovina	2452.6 (1970.7-2962.1)	65.1 (51-80.1)	66517.1 (54658.5-78825.5)	1562.8 (1272.7-1867.5)	2744.3 (1990.1-3576.5)	43.6 (31.6-56.8)	58680.7 (44057.7-75111.3)	975.1 (733.1-1244.4)	-1.56 (-1.75--1.37)	-1.77 (-1.94--1.6)
Botswana	151.6 (105.7-203.3)	30.9 (21.5-41.3)	4319 (3025.2-5797)	744.2 (521.2-994.1)	240.4 (171.4-329.1)	18 (12.9-24.5)	7041.3 (4972.1-9679.5)	442 (315.9-601.8)	-1.99 (-2.25--1.72)	-2.01 (-2.32--1.7)

Brazil	43055.1 (35966.7-50208.8)	51.3 (42.1-61.4)	1206273.8 (1032834.2-1374290)	1269.4 (1073.5-1467.7)	36394.3 (29290.7-44837.8)	14.5 (11.6-17.9)	986898.7 (816297.2-1204403.3)	384.3 (317.2-469.1)	-4.15 (-4.33--3.98)	-4.01 (-4.16--3.85)
Brunei Darussalam	50 (39.9-61.3)	49.4 (38.5-61.2)	1564.4 (1255.6-1886.8)	1257.3 (1003.5-1543.5)	59.2 (46.5-77.2)	16.8 (12.8-21.8)	2022.1 (1598.4-2604.4)	466 (367.2-604.8)	-3.2 (-3.52--2.89)	-3.11 (-3.45--2.76)
Bulgaria	10598.6 (8661-12509.9)	101.1 (81.7-121.4)	276840.3 (232607-322008)	2391.4 (2009.8-2776)	6528.9 (4946-8257.3)	49.6 (38-62.4)	163669.8 (128478.5-203890.4)	1360.3 (1084.1-1684.6)	-2.84 (-3.11--2.57)	-2.43 (-2.7--2.17)
Burkina Faso	322 (223.9-445.6)	8.1 (5.7-11.3)	9433.5 (6576.3-12952.2)	207.2 (145.5-285)	637 (435.6-901.8)	7.3 (5-10.4)	18725.6 (12732.8-26553.7)	184.6 (126.9-259.3)	-0.22 (-0.31--0.13)	-0.31 (-0.42--0.2)
Burundi	347 (235.6-498.5)	15.2 (10.4-21.9)	10660.8 (7319.6-15115.6)	419.4 (289.3-598.6)	341.9 (234.3-477.3)	6.7 (4.6-9.5)	11538.3 (7997.6-16079.8)	191 (132-265.4)	-3 (-3.49--2.51)	-2.9 (-3.38--2.42)
Cabo Verde	20.4 (14.9-28)	9.1 (6.7-12.4)	544.6 (403.8-728.8)	255.8 (189.4-340.8)	40.4 (29.4-53.9)	9 (6.6-12.1)	1116 (814.1-1488)	232.9 (169.9-308.4)	-0.66 (-1.08--0.23)	-0.78 (-1.08--0.48)
Cambodia	1617.3 (1275.6-2067.6)	40.5 (31.5-52.1)	46750 (37232.6-59371.8)	982.4 (776.8-1246.2)	4094 (2899.4-5328.4)	39.1 (27.5-51.1)	108815 (78247.9-142857.9)	866.9 (618.7-1128)	-0.34 (-0.47--0.21)	-0.66 (-0.8--0.52)
Cameroon	390 (262.9-547)	9.4 (6.5-13.3)	11853.7 (8100.8-16460.2)	242.9 (166.5-337.5)	1064.2 (664-1576.2)	8.7 (5.5-12.7)	33753.9 (21040.4-49855.4)	229 (145.1-336.3)	-0.22 (-0.85-0.41)	-0.19 (-0.86-0.49)
Canada	13940.5 (11462.6-	43.3 (35.7-	322541.6 (271778.2-	1011.5 (852.1-	6766.5 (5277.6-	9.2 (7.3-	153735 (125159.2-	236.6 (194.3-	-5.26 (-5.46--	-4.87 (-5.06--

	16662)	51.7)	374848.6)	1173.7)	8394.4)	11.3)	185180.4)	285.5)	5.07)	4.69)
Central African Republic	233.4 (155.4-339.4)	20.5 (13.6-29.6)	7413.9 (4899.3-10770)	559 (374.7-808.9)	331.9 (212.7-506.6)	14.3 (9.2-21.2)	11241.3 (7146.9-17290.3)	398.9 (258.6-600.5)	-1.35 (-1.51--1.19)	-1.3 (-1.45--1.15)
Chad	302.3 (204.7-426.4)	11.4 (7.7-16)	8089.9 (5582.1-11373.7)	279.3 (193-392.1)	603.8 (398.2-891.7)	11.4 (7.4-16.8)	17752.7 (11733-25933.6)	280 (186.7-409.1)	-0.3 (-0.57--0.03)	-0.3 (-0.59--0.01)
Chile	2150.7 (1725.2-2673.4)	22.7 (18-28.7)	55845.9 (46092.1-66994)	545.7 (445.8-657)	1779.1 (1411-2192.8)	7 (5.6-8.6)	50278.9 (41691.3-59418.2)	204.7 (170.7-241.5)	-3.62 (-3.69--3.54)	-3.02 (-3.09--2.94)
China	269566.8 (217710-331411.1)	39.6 (31.3-49.2)	7391611.5 (6030654.2-8950883)	885.4 (719.1-1077.7)	708851.1 (534848.5-910239.2)	37.3 (27.9-48)	15794471.3 (12178000.8-19969843.5)	776.3 (601.1-978.4)	0.14 (-0.09-0.38)	-0.13 (-0.31-0.04)
Colombia	3912.4 (3238.8-4641)	22.7 (18.6-27.2)	112721.5 (95109.6-132524.9)	587.3 (491-695)	4127.1 (3083.1-5443.4)	7.4 (5.5-9.7)	102690.8 (78604.8-133084.9)	185.2 (141.5-239.6)	-4.03 (-4.25--3.82)	-4.11 (-4.31--3.9)
Comoros	27.2 (17.8-39.5)	15.7 (10.3-22.5)	792 (517.2-1138.6)	385.1 (254.5-546.9)	47.5 (31-67.5)	10.8 (7-15.6)	1320.8 (872.9-1862)	257.9 (170.6-362.6)	-1.64 (-1.84--1.45)	-1.76 (-1.99--1.52)
Congo	223.8 (154.7-302.8)	21.7 (15.3-29.4)	6722.7 (4577.7-9213.5)	575.2 (396.7-779.4)	468 (313.9-655.7)	18.2 (12.5-25.5)	14518.7 (9813-20681.2)	462.6 (312.6-645)	-0.81 (-1.04--0.57)	-0.98 (-1.24--0.72)
Cook Islands	4.6 (3.6-5.8)	37.2 (28.5-47)	145.2 (111.6-183.5)	1056.4 (816.4-1337.5)	5.4 (4-7.3)	21.7 (16.1-29.4)	153.1 (114.6-207)	639.9 (479.9-869.9)	-1.74 (-1.88--1.61)	-1.54 (-1.69--1.39)
Costa Rica	396 (319.9-	23.4	9873.1 (8135.9-	548	478.1 (365.6-	8.6	12526.7 (9744.1-	226.5	-3.36 (-	-3.05 (-

	488.7)	(18.8-29)	11843.9)	(447.2-661.1)	607)	(6.6-10.9)	15707.2)	(176-283.9)	3.51--3.21)	3.22--2.89)
Coted'Ivoire	717.8 (494.1-963.9)	19.9 (13.9-27.3)	22753.2 (15716.7-30594.4)	497.3 (348.3-665.3)	1607.3 (1087.8-2315.7)	15.3 (10.6-21.7)	50478.2 (33845.7-72460.5)	384.8 (264.9-549.1)	-1.59 (-2.13--1.05)	-1.57 (-2.1--1.04)
Croatia	5327.1 (4256.9-6556.6)	98.9 (78.3-123)	115609.9 (95287.9-136513.8)	1975.9 (1623.4-2373.8)	3162.7 (2385.3-4112.1)	33.4 (25.5-43.1)	57909.8 (45291-72922.9)	679.4 (537.5-845.9)	-3.26 (-3.39--3.14)	-3.25 (-3.36--3.14)
Cuba	5523.7 (4396.3-6625.4)	56 (44.3-67.9)	132404.6 (109380.8-154801.2)	1307 (1080.8-1529.4)	4553.7 (3447-5863.4)	22.8 (17.5-29.3)	106275.2 (83694.8-132784.3)	559.3 (442.1-694.4)	-3.27 (-3.5--3.04)	-3.08 (-3.3--2.85)
Cyprus	367.5 (295.7-446.1)	58.3 (45.5-72.8)	8608.1 (7169.8-10192.6)	1176.8 (968.3-1408.7)	358.9 (281.2-457)	18.7 (14.5-23.8)	8263.1 (6656.9-10201)	424.8 (341.9-525.4)	-3.92 (-4.05--3.79)	-3.47 (-3.6--3.33)
Czechia	12719.2 (10321.8-15508.3)	93.3 (75.5-113.7)	305118.7 (254812.6-358187.8)	2262.3 (1902-2654.1)	5529.4 (4175.6-7003.3)	25.2 (19.3-31.7)	116540.9 (91553.1-143782.2)	582.6 (460.4-714.7)	-4.21 (-4.28--4.15)	-4.35 (-4.45--4.26)
Democratic People's Republic of Korea	4988.7 (3518.7-6785.9)	34.1 (23.9-46.4)	150378 (105742.4-202132.9)	872.1 (620-1177.2)	10843.8 (7845.6-14507.2)	33.9 (24.5-45.9)	311731.9 (226750.9-417620.5)	925.3 (673.8-1233.8)	-0.19 (-0.37--0.01)	0.01 (-0.17-0.19)
Democratic Republic of the Congo	1789 (1189.8-2569.1)	12.1 (8.2-17.2)	55257.6 (36796.5-78221.9)	316.7 (212.8-450)	3019.1 (2048.4-4386.9)	8.2 (5.5-12)	97823 (66401-140371.2)	222.2 (152-320)	-1.41 (-1.66--1.16)	-1.28 (-1.52--1.03)
Denmark	6488.5 (5343.7-7686.6)	77.6 (64.3-91.3)	128367.2 (109268-148581.4)	1648.9 (1415.9-1891)	1316.6 (1009.6-1646.9)	10.5 (8.2-12.9)	26237.3 (21485.4-31729.9)	236.3 (195-282)	-6.92 (-7.11--6.73)	-6.61 (-6.79--6.43)

Djibouti	21.9 (13.7-33.5)	18.6 (12-28.1)	714.5 (451.1-1074.9)	461.8 (300.6-686.6)	115.1 (73.3-165.8)	20.5 (13.1-29.7)	3641.8 (2314.9-5237.3)	498.7 (327.7-708.1)	0.28 (0.2-0.36)	0.21 (0.11-0.31)
Dominica	10 (7.8-12.7)	17.3 (13.4-21.8)	231.5 (185.5-283.8)	403.1 (324.4-492.3)	8.2 (6.1-11.1)	10 (7.4-13.5)	206.3 (156-277.1)	243.3 (184.1-327.2)	-1.9 (-2.28--1.51)	-1.7 (-2.09--1.31)
Dominican Republic	1188 (921.6-1504.6)	36.4 (27.8-46.8)	30910 (24275.7-38839.8)	808.9 (631.1-1022.2)	2900.7 (2056-3954.1)	29.4 (20.7-40.1)	72147.6 (51805.9-96331.8)	706.1 (509.5-945.9)	-0.16 (-0.45-0.14)	0.02 (-0.19-0.22)
Ecuador	831.3 (675.2-1004.9)	16.6 (13.3-20.2)	22476.4 (18595.7-26577.1)	399.8 (329.5-474.6)	1157.4 (833.7-1512)	7.3 (5.2-9.5)	30888.3 (22518.5-40002.7)	185.4 (135.1-240.1)	-2.76 (-3.26--2.26)	-2.59 (-3.06--2.11)
Egypt	23748.5 (18811.7-29791.7)	100.9 (78.1-129.1)	711154 (573030.5-888136.6)	2382.5 (1893.5-2982)	50088.1 (37848.7-64347.9)	90.6 (67.6-117.1)	1489287.8 (1136323.5-1895854.5)	2135.1 (1620.3-2731.9)	0 (-0.22-0.22)	-0.05 (-0.26-0.16)
El Salvador	324.4 (255.4-405.2)	10.8 (8.4-13.5)	9686.2 (7668.1-11851.4)	303 (240.2-372.8)	581.6 (412.5-781.4)	9.3 (6.6-12.5)	15973.2 (11655.5-21327.4)	262.5 (191.9-350.2)	-0.48 (-0.71--0.25)	-0.42 (-0.69--0.15)
Equatorial Guinea	40.4 (26.9-58.3)	21.5 (14.4-30.9)	1231.5 (816.5-1756.6)	573.7 (383.9-818.9)	71.3 (43.2-104.6)	14.8 (9-21.2)	2222.7 (1338-3290.9)	369.7 (224.5-536.5)	-1.51 (-1.85--1.16)	-1.77 (-2.14--1.39)
Eritrea	111.5 (70.3-173.3)	8.7 (5.4-13.5)	4181.4 (2665.5-6438.1)	265.4 (169.8-408)	225.4 (139.4-338)	7.3 (4.6-10.9)	8358.2 (5197.5-12537.3)	218.1 (137.3-323.7)	-0.75 (-0.85--0.65)	-0.86 (-0.97--0.75)
Estonia	1572.6 (1266.2-	78.4 (62.9-	39738.6 (33139.8-	1982.3 (1657.5-	438.4 (333.9-546.4)	16.1 (12.5-	9810.1 (7832.5-11898)	412.8 (334.6-	-5.72 (-6.29--	-5.74 (-6.26--

	1944.8)	97.3)	47206.4)	2357.7)		19.7)		498.4)	5.15)	5.21)
Eswatini	38.4 (26.9-51.7)	15.7 (10.9-21.5)	1073.2 (768.2-1444.1)	364.5 (259-490.9)	64.4 (38.4-96.5)	12.7 (7.7-18.9)	1960.6 (1174.3-2976.4)	315.8 (191.3-468.3)	-0.35 (-0.84-0.15)	-0.13 (-0.69-0.45)
Ethiopia	1274.4 (898.7-1846.1)	6 (4.2-8.6)	42309.5 (30300.6-60240.9)	178.4 (127.4-253.7)	1345.2 (954.4-1812.3)	3.1 (2.2-4.2)	42717.7 (30866.7-57876.6)	84.5 (60.8-114)	-2.2 (-2.44--1.97)	-2.51 (-2.76--2.26)
Fiji	281.3 (225.3-346)	70.1 (55.4-86.6)	9893 (7964.4-12101.3)	2126 (1703.3-2605)	399.4 (295.3-539.7)	49.8 (36.9-66.8)	13030.4 (9576.6-17647.7)	1470.8 (1085.6-1988.9)	-1.28 (-1.43--1.14)	-1.32 (-1.45--1.18)
Finland	2733.3 (2228.2-3318.8)	39.3 (32.3-47.3)	71437.9 (59832.3-84758.2)	1065.9 (897.8-1256.5)	1241.9 (957.4-1561.8)	9.8 (7.8-12)	26915.8 (21828.7-32935.3)	251.7 (208.5-302.7)	-4.3 (-4.42--4.18)	-4.45 (-4.55--4.34)
France	17268.8 (13746-20992.4)	20.6 (16.6-24.7)	380646.7 (316292.8-446712.6)	489.2 (412.2-569.8)	7858.2 (6040.4-9941.5)	5.4 (4.2-6.5)	178846.6 (144768.9-216316.2)	151.4 (125.7-178.7)	-4.43 (-4.53--4.33)	-3.79 (-3.86--3.72)
Gabon	70.6 (50-96.2)	12.9 (9.1-17.5)	2020.6 (1417.9-2719.2)	343.2 (240.3-462.3)	124.1 (80.4-172.4)	12.2 (8-16.6)	3794.7 (2465.7-5298.2)	319.9 (210.8-443.5)	-0.28 (-0.39--0.17)	-0.3 (-0.41--0.19)
Gambia	76.1 (51.6-104.8)	23.6 (15.9-32.6)	2244.4 (1537-3064.2)	591.7 (405.5-806.4)	166.3 (113.4-230.1)	18.1 (12.2-25.3)	4805.2 (3298.1-6559.8)	448.5 (307.9-609)	-1.12 (-1.33--0.92)	-1.18 (-1.4--0.96)
Georgia	4943.2 (3890.8-6010.2)	82.3 (64.1-100.7)	129115.3 (105829.9-153268.5)	2080.6 (1699.3-2474)	2849.1 (2262.3-3491.8)	47.3 (38-57.6)	65934.8 (54632-78936.6)	1188.5 (994.6-1418.4)	-2.16 (-2.48--1.84)	-2.43 (-2.82--2.04)
Germany	61311.1	47.9	1370027.9	1136.4	22530.9	11.2	481996.7	284.9	-4.82 (-	-4.56 (-

	(49838.4-73683.5)	(39.5-57.4)	(1150127.2-1609035.7)	(965.9-1322.7)	(17291.9-27932.3)	(8.9-13.7)	(388449.6-587371)	(234-342)	5.02--4.62)	4.73--4.38)
Ghana	767.2 (563.1-1017)	13.8 (10-18.2)	23200.6 (17140.8-31072)	340.2 (251.5-450.3)	1694.8 (1204.3-2311.8)	11.5 (7.9-15.4)	50308.7 (36275.2-68513.2)	275 (197.2-372.7)	-0.42 (-0.58--0.25)	-0.5 (-0.67--0.33)
Greece	8394.8 (6830.7-10084.1)	57.4 (46.6-69)	182905.2 (155258.1-211872.8)	1258.4 (1075.9-1452.8)	5164.9 (4007.8-6519.6)	21 (17.1-25.5)	107477.1 (88583.5-128426.3)	545.7 (462.9-637.3)	-3.49 (-3.69--3.3)	-2.85 (-3--2.7)
Greenland	23.2 (18.7-28)	72.1 (56.9-90.3)	729.2 (595.4-878.3)	1860.4 (1490-2245.9)	15.2 (11.7-19.4)	22.3 (17.1-28.8)	443.7 (348.6-559.2)	591.3 (464.1-741.6)	-3.89 (-4.04--3.75)	-3.8 (-3.92--3.68)
Grenada	16.2 (12.5-20.7)	23.1 (18.1-29.3)	409.2 (326-508.1)	635.6 (508-785.3)	11.9 (9.2-15.2)	10.4 (8-13.5)	330.8 (260-412.2)	273.3 (214-342.6)	-3 (-3.23--2.78)	-2.99 (-3.21--2.77)
Guam	25.9 (21-31.9)	33.5 (26.5-42.8)	863.2 (694.1-1056.5)	931.8 (751.8-1145.7)	58.7 (45.7-74.3)	28.5 (22.3-35.9)	1911.8 (1518.1-2376.5)	966 (773.2-1208.3)	0.03 (-0.19-0.24)	0.58 (0.43-0.74)
Guatemala	488.7 (397.2-599.9)	16.1 (12.8-20)	14250.4 (11742.8-17247.4)	377.9 (307.2-463.1)	865.1 (648.6-1123.1)	8.5 (6.3-11)	22273.2 (16977.2-28121.2)	195 (149.1-248.6)	-2.28 (-2.63--1.92)	-2.38 (-2.78--1.97)
Guinea	389.1 (269.5-530)	12.7 (8.7-17.3)	10319.7 (7379-13965.1)	306.2 (219.1-413.2)	723.2 (473.7-1006.8)	13.7 (9-19)	20268.7 (13347.9-28115.3)	336.8 (221.8-466)	0.38 (0.23-0.54)	0.47 (0.29-0.64)
Guinea-Bissau	45.7 (31-64.6)	11.8 (8.1-16.6)	1413.8 (959.3-1984.8)	320.4 (219.1-448.7)	98.1 (65-137.6)	13.6 (9.1-19.2)	3256.4 (2143.6-4617.3)	365.6 (245.1-509.3)	0.84 (0.65-1.03)	0.81 (0.63-0.99)

Guyana	153.6 (121.8-190.2)	40.2 (31.6-50.2)	4653.8 (3731.8-5767.1)	1099.4 (881.2-1360.7)	125.6 (89.5-172.3)	19.5 (13.9-26.7)	3732 (2659.8-5151.1)	532.1 (381.2-730.4)	-1.96 (-2.14--1.79)	-1.96 (-2.13--1.8)
Haiti	845.3 (631.3-1085.8)	26.7 (19.7-34.6)	26441 (19897.3-33565.6)	728.6 (544.8-932.8)	1085.5 (719.8-1529.2)	15.5 (10.2-21.7)	33657.8 (22490.7-47415.8)	401.9 (267.8-564.3)	-1.65 (-1.88--1.41)	-1.81 (-2.06--1.56)
Honduras	405.1 (308.3-530.6)	21.1 (15.8-28.1)	11369.3 (8759.2-14643)	520.1 (400.3-674.3)	1485.7 (1065.8-1985.6)	25.8 (18.4-34.7)	37527 (27180.8-50668.7)	580.2 (419.9-780.2)	0.92 (0.73-1.12)	0.57 (0.4-0.73)
Hungary	11522.3 (9637.6-13521.3)	81.8 (68.6-96.1)	309959.5 (266799.9-356144.9)	2218.6 (1912.2-2543)	5312.3 (4139.8-6674.6)	27.8 (21.9-34.7)	126024.7 (100927-154440.3)	729.2 (591.1-884)	-3.74 (-3.88--3.6)	-3.92 (-4.05--3.79)
Iceland	136.8 (109.8-166.7)	47 (38.2-56.7)	2996.9 (2495.8-3516.1)	1082.5 (906.6-1260.3)	61.8 (47-79.9)	10.2 (7.9-13)	1329.4 (1075.2-1649.5)	246.9 (201-303.4)	-5.12 (-5.22--5.02)	-5.01 (-5.17--4.86)
India	151559.3 (121910.8-186377.7)	33.9 (26.6-41.8)	4759032.6 (3922722.6-5800890.7)	901.7 (727.5-1106.4)	308010.9 (244588.1-382554.3)	27.1 (21.4-34)	8559182.5 (6835409-10430991.7)	682 (546.7-835.3)	-0.69 (-0.81--0.58)	-0.91 (-0.99--0.83)
Indonesia	28283 (22148.6-35122.7)	30.9 (23.7-39)	897390.7 (706903.4-1097879.4)	809.7 (638.6-997.5)	93039.2 (68738.4-121043.6)	43.8 (32-56.5)	2740490.6 (2055919.3-3558983.8)	1060.3 (792.5-1370.1)	1.22 (1.05-1.39)	1 (0.87-1.14)
Iran (Islamic Republic of)	11837.8 (9624.5-14263.3)	47.2 (37.6-57.4)	361537.5 (296424.8-431021.9)	1230.7 (999.4-1478.1)	18617.1 (15022.6-22582.5)	24.5 (19.3-30.2)	526755.9 (438904.3-623891.5)	616 (507.5-738.8)	-2.17 (-2.3--2.05)	-2.27 (-2.38--2.16)
Iraq	8145.9 (6221.8-	106.8 (81.7-	213132.4 (167651.1-	2592.6 (2037.5-	16748 (11940-	82.1 (59-	442538.7 (314994-	1789.2 (1285.4-	-1.42 (-1.6--	-1.7 (-1.85--

	10249.5)	134.9)	265186.9)	3233.1)	22060.6)	107.7)	585894.9)	2358)	1.23)	1.55)
Ireland	3154.2 (2582.8-3758.5)	78.4 (64.1-93.5)	67544.5 (56398.2-79021.8)	1690.5 (1420.2-1975.3)	766.9 (577.1-963.2)	9.4 (7.2-11.8)	16227.9 (12976.5-19830.7)	214 (172.7-259.6)	-7.07 (-7.31--6.83)	-6.92 (-7.14--6.69)
Israel	1879.7 (1538.5-2297.3)	40.2 (32.6-49.1)	44325.1 (37211-52395.3)	945.9 (795.8-1113.9)	694.6 (522.5-888.3)	5.5 (4.2-6.9)	16333.8 (13121.5-20181.9)	140.7 (114.9-173.2)	-6.81 (-6.99--6.63)	-6.48 (-6.68--6.27)
Italy	28264.9 (22991.1-33789.9)	32.3 (26.4-38.6)	630991.5 (531774.2-733332.5)	746.6 (633.7-863.3)	12123.4 (9323.5-15368.6)	7.6 (6.1-9.4)	237374 (191119.1-289120.3)	184.1 (152.2-220.4)	-4.88 (-5--4.77)	-4.69 (-4.8--4.58)
Jamaica	291.9 (225.8-367.9)	16.2 (12.6-20.3)	6591.6 (5269.6-8043.8)	378.4 (305.2-460.6)	304.8 (210.1-424.4)	9.5 (6.6-13.2)	7147.5 (5111.2-9748.5)	230 (164-313.9)	-1.64 (-2.12--1.17)	-1.59 (-2.06--1.12)
Japan	36197.4 (29525.3-43759.5)	22.6 (18.3-27.5)	824032.4 (698222.1-960803.1)	494.4 (416.7-578.3)	21890.3 (16672-27576.1)	5.9 (4.7-7.1)	461543.2 (370238.4-560133.2)	168 (139.9-198.3)	-4.48 (-4.58--4.38)	-3.59 (-3.65--3.52)
Jordan	976.2 (774.2-1217.6)	80.2 (61.9-101.5)	29115.9 (23377.1-35869)	1943.6 (1552.6-2416.8)	2137.3 (1607.4-2790)	31.6 (23.3-41.6)	66007.9 (50456.9-84256.6)	773.9 (585.3-995.5)	-3.56 (-3.84--3.28)	-3.52 (-3.78--3.26)
Kazakhstan	8036.3 (6464.5-9944.3)	64.9 (51.3-81.6)	236916 (197434.7-284285.4)	1761.1 (1450.7-2129.6)	6327.8 (4839.6-7884)	38.5 (28.8-48.4)	170760 (134138.1-210297.8)	920 (715.1-1133.8)	-2.7 (-3.38--2)	-3.22 (-3.99--2.44)
Kenya	494.7 (339.5-684.3)	6.9 (4.7-9.7)	14532.7 (10316.2-19525.5)	170 (120.1-231.1)	1317.1 (931.1-1820.3)	6.3 (4.3-8.6)	41211.6 (29559.1-56157.3)	160.5 (115.3-218.9)	-0.56 (-0.81--0.31)	-0.4 (-0.68--0.11)
Kiribati	26.4 (20.8-	69.1	900 (706-	2068.6	59.2 (44.2-	78	2047 (1523.3-	2305.5	0.32	0.29

	33.2)	(54.4-87.5)	1133.2)	(1633.5-2600.3)	76.1)	(58.7-100.7)	2634.9)	(1724-2942)	(0.16-0.48)	(0.15-0.43)
Kuwait	360.7 (294.7-426.6)	56.9 (45-68.7)	12299.1 (10231.4-14412.8)	1472.7 (1203.1-1752.4)	995.4 (745.4-1263)	31.3 (22.7-40.6)	33444.6 (25601.7-42334.4)	795.8 (602.2-1015.4)	-1.84 (-2.31--1.36)	-1.88 (-2.33--1.42)
Kyrgyzstan	1859.9 (1460.2-2300.9)	66.5 (51.8-82.9)	49731.4 (40185.6-59610.3)	1644.5 (1321.1-1983.6)	3198.3 (2528.6-4024.1)	75.4 (59.2-96.3)	82516.5 (65906.8-102475.1)	1661.9 (1322.8-2077.5)	0.97 (0.58-1.37)	0.25 (-0.15-0.65)
Lao People's Democratic Republic	1409.1 (1047.4-1831.8)	73 (54.3-95.5)	42093.8 (30882.1-54663.5)	1878.5 (1391.1-2440)	2118 (1532.5-2782.7)	51.2 (37-67.1)	60460.7 (44119-79527.4)	1207.2 (880.9-1575)	-1.21 (-1.3--1.13)	-1.49 (-1.57--1.42)
Latvia	2936.8 (2390.8-3607.5)	83.9 (68-102.9)	74747.8 (63065-89162.5)	2149.9 (1817.9-2561.1)	1273.2 (976.5-1602)	32.5 (25.5-40.3)	29302.4 (23262.6-35898.9)	850.4 (686.1-1028.4)	-3.53 (-3.9--3.17)	-3.65 (-4.07--3.24)
Lebanon	1611.9 (1213.6-2092.4)	78.2 (57.9-101.4)	45067.3 (34510.2-58017.2)	1967.3 (1497.9-2540.3)	1953.5 (1481.2-2520.3)	30.8 (23.6-39.7)	43707.6 (34062.2-54615.5)	722.9 (565.4-903)	-3.13 (-3.37--2.88)	-3.29 (-3.59--3)
Lesotho	101.6 (68.6-143.3)	13.6 (9.1-19.4)	2483.5 (1724.9-3426.3)	299.2 (206.4-415.5)	260.5 (158.3-432.3)	27.4 (16.6-44)	7278.4 (4364.4-12355.3)	654.4 (396.7-1085.7)	3.28 (2.81-3.75)	3.46 (2.97-3.95)
Liberia	112.6 (82.5-149.3)	10.4 (7.6-13.8)	3234.5 (2364.4-4259.9)	270.5 (198.7-355.5)	184 (123.8-264)	8.6 (5.8-12.1)	6029.6 (4051.4-8664.6)	230.1 (154.9-326.3)	-0.87 (-1.05--0.69)	-0.74 (-0.91--0.57)
Libya	775.4 (568.8-1004.4)	41.6 (30.3-54.2)	22595.2 (16970.6-28843.1)	1067.9 (799.4-1367.9)	2352.2 (1630.9-3213.8)	44.6 (30.6-61.3)	72861.2 (51750.4-98485.8)	1152.9 (811.2-1564.9)	0.76 (0.53-0.99)	0.69 (0.48-0.89)

Lithuania	3185 (2554-3909.5)	71.9 (57.9-88.3)	78992.4 (65879.9-92995.1)	1796.5 (1505.1-2116.7)	1872.6 (1464.2-2371.4)	32.1 (25.6-39.8)	40989 (33451.5-50492.2)	807.6 (668.4-980.3)	-2.72 (-3.02--2.41)	-2.65 (-3.01--2.29)
Luxembourg	219.7 (171-275.6)	40.8 (31.8-51.3)	5097.8 (4077-6200.9)	967.7 (774-1174.9)	93.1 (68.8-120.8)	8.4 (6.3-10.8)	2079.9 (1599.7-2628.2)	202.5 (157.6-254.4)	-5.25 (-5.39--5.1)	-5.33 (-5.51--5.16)
Madagascar	825.6 (629.5-1073.5)	17.9 (13.3-23.8)	25318.7 (19583.4-32290.9)	459.7 (353.8-591.4)	1115.1 (743.4-1584.7)	10 (6.6-14.3)	39308.9 (26647.9-56186.6)	272.2 (183.5-383.1)	-2.28 (-2.51--2.05)	-2.08 (-2.29--1.88)
Malawi	478.9 (357.3-625.4)	14.4 (10.6-18.6)	13778.5 (10625.9-17826)	346.1 (262.4-447.9)	1204.6 (847.5-1642.7)	17.2 (12.2-23.4)	36446.8 (25606.4-49377.6)	437.3 (309.4-592)	0.31 (0-0.61)	0.45 (0.11-0.8)
Malaysia	4300.9 (3490.5-5247)	48 (38.3-59.1)	120493.5 (99669.1-144274.8)	1209.3 (991.9-1456.1)	8626.2 (7048.4-10661.4)	31.6 (25.2-39.6)	248023.2 (204531.2-301756.4)	826.4 (676-1014.7)	-1.43 (-1.6--1.25)	-1.2 (-1.32--1.08)
Maldives	50.4 (39.4-62)	66.4 (50.8-83.3)	1548.5 (1227.5-1905.5)	1605.4 (1261.1-1970.2)	74.2 (54.1-96.1)	24 (17.6-31.6)	2033 (1510.6-2607.7)	510.7 (378.2-656.7)	-3.75 (-3.91--3.59)	-4.23 (-4.45--4.02)
Mali	242.3 (168-346.4)	7.3 (5-10.5)	6908.4 (4836.3-9748.3)	170.8 (120-239)	735.6 (495.2-1038.1)	10 (6.8-14.1)	20504.7 (14023.7-28714.9)	226.3 (155-314.8)	1.37 (1.25-1.49)	1.24 (1.12-1.37)
Malta	206.8 (169.4-248.1)	49.3 (40.3-59.3)	5050.8 (4232.1-5863.2)	1182.7 (990.9-1377.2)	100.6 (76.3-127.9)	10.9 (8.5-13.7)	2316.3 (1853.4-2876)	291.6 (238-355.8)	-4.93 (-5.07--4.78)	-4.56 (-4.68--4.44)
Marshall Islands	8.9 (6.7-12.1)	52.7 (38.6-	305.6 (233.3-408.7)	1554.6 (1177.5-	20.6 (14.6-28.1)	53.7 (37.6-	749.7 (530.2-1013.5)	1628.5 (1149.8-	0.12 (0.03-	0.18 (0.08-

		72.5)		2107.7)		74.5)		2222)	0.22)	0.29)
Mauritania	182.1 (126.5-261.3)	19.5 (13.5-28.1)	5234.1 (3701-7496.9)	498.7 (352.4-709.1)	229.9 (144.8-339.3)	11.5 (7.2-16.9)	6496.7 (4134.9-9467.7)	281.3 (179-407.4)	-2.11 (-2.32--1.89)	-2.22 (-2.42--2.01)
Mauritius	467.4 (388.9-552.5)	66.3 (54.4-79.7)	13678.8 (11490.9-15968.7)	1729 (1441.4-2027.9)	434.7 (350.8-529.9)	24.8 (19.9-30.3)	11621.6 (9638.7-13953.4)	654.4 (542.2-784.2)	-3.74 (-4.12--3.36)	-3.59 (-3.98--3.21)
Mexico	8916.3 (7331-10536.5)	24.2 (19.3-29.1)	230261.9 (195750.4-264989.3)	528.3 (441.9-615.8)	13834.2 (10897.6-17182.1)	11.3 (8.8-14.1)	366027.2 (294554.5-448171)	279.6 (223.8-343.7)	-2.82 (-3.04--2.59)	-2.45 (-2.7--2.19)
Micronesia (Federated States of)	36.5 (27.2-47.5)	73.2 (55.1-94.9)	1224.1 (897.6-1599.5)	2231.7 (1647.7-2914.1)	56 (41-75.4)	69.7 (50.6-92.9)	1931.4 (1417.3-2605.8)	2145.5 (1584.7-2878.7)	-0.12 (-0.15--0.1)	-0.07 (-0.1--0.04)
Monaco	22 (15.3-29.6)	30.7 (22-40.2)	452.9 (330.5-583.5)	737.4 (544.1-940)	10.9 (7.7-15.2)	10.9 (7.7-15)	225 (162.4-309)	275.3 (199.1-378)	-3.49 (-3.62--3.37)	-3.31 (-3.41--3.2)
Mongolia	538.7 (422.3-674.1)	54.2 (41.9-68.2)	14858.3 (11678.4-18416.2)	1364.6 (1072-1691.1)	941.5 (709.8-1207)	44 (32.4-57.8)	28382.9 (21995.4-35343.9)	1091.6 (831.6-1388.3)	-0.88 (-1.13--0.62)	-0.9 (-1.2--0.6)
Montenegro	316.8 (249.1-380.2)	51.9 (40.7-62.7)	8499.1 (6967.3-10044.4)	1331.4 (1087.4-1580.9)	487.4 (357.3-619.7)	53.4 (38.7-68.3)	11086.3 (8590.1-13798.6)	1170.7 (902-1460.1)	0.13 (-0.07-0.33)	-0.42 (-0.7--0.14)
Morocco	8713 (6608.3-11204.6)	63 (47.1-82.1)	249338.1 (191325.9-316010.2)	1630.3 (1245.8-2069.2)	12907.6 (8626.1-17410.2)	39.3 (26.2-53.3)	348434.4 (239276.8-467057.8)	964.6 (657.9-1300.2)	-1.7 (-1.86--1.55)	-1.89 (-2.04--1.75)
Mozambique	425 (310.8-	8.3 (6.1-	12230.7	200.5	1052.6	10.1	32954.4	263.6	1.18	1.49

	559.9)	11.2)	(9123.6-15827.4)	(149.3-260.2)	(688.8-1441.3)	(6.8-13.9)	(21677.2-45177.7)	(175.6-359.6)	(0.99-1.38)	(1.26-1.72)
Myanmar	14808.9 (10912.8-19321.1)	72.1 (53.9-93.6)	415001.5 (304260.4-545545.5)	1729.5 (1281.2-2263.9)	12867.9 (9420.3-16705.1)	29.4 (21.4-38.5)	332856.6 (245238.9-433233.5)	671.1 (497.5-867.6)	-3.12 (-3.21--3.03)	-3.28 (-3.37--3.19)
Namibia	158.3 (118.5-204.5)	31.1 (23-41.1)	4147.8 (3105.7-5283.2)	668.7 (500.6-860.1)	249.3 (175.5-331.5)	21.9 (15.4-29.7)	6444.1 (4526.3-8656.6)	471.2 (335.5-620.6)	-1.49 (-1.82--1.15)	-1.51 (-1.86--1.16)
Nauru	5.9 (4.4-7.5)	116.6 (87.7-148.7)	211.8 (155.8-271.5)	3537 (2633.5-4509.5)	6.9 (5.1-9.2)	107 (78.3-139)	252.2 (183-340.9)	3356.9 (2463.8-4432.8)	-0.4 (-0.72--0.07)	-0.28 (-0.62-0.06)
Nepal	4072.1 (3015.8-5327.2)	48.1 (35.3-63.8)	118342.6 (87301.9-154376.2)	1175.1 (874.7-1527.9)	7141.4 (5295.9-9372.6)	34.1 (25.1-45.1)	182118.9 (135016.8-238782.6)	772.1 (575.2-1013.8)	-1.09 (-1.21--0.97)	-1.36 (-1.47--1.25)
Netherlands	8340.8 (6870.3-10019.1)	41.9 (34.7-50.1)	192495.1 (163378-223540.6)	1004.7 (858.2-1162.7)	2896.1 (2231.5-3670.4)	7.8 (6.1-9.7)	58313.2 (46903.7-71986.6)	176.2 (143.8-215.3)	-5.73 (-5.92--5.54)	-5.9 (-6.08--5.72)
New Zealand	1617.6 (1331.4-1931.4)	41.4 (34.2-49.5)	39062.8 (32796.1-45706.5)	1020.8 (860-1191.9)	816.1 (637.3-1017.3)	9.5 (7.5-11.7)	17683.4 (14519.6-21178.8)	226.6 (186.8-270.5)	-4.94 (-5.16--4.73)	-5.1 (-5.37--4.83)
Nicaragua	212.4 (166.8-267.6)	14.7 (11.4-18.9)	5848.6 (4687.2-7156.7)	352.5 (279.8-438.1)	491.3 (360.2-654.9)	10.6 (7.7-14.3)	13110.7 (9859-17116)	254 (190-332.7)	-0.68 (-0.87--0.48)	-0.75 (-0.94--0.56)
Niger	119.1 (74.5-180.4)	5.2 (3.3-7.9)	3607.2 (2295-5412.9)	123.5 (79.9-181.9)	308.2 (186.3-465.8)	4.8 (2.9-7.2)	8824.2 (5410.7-13110.7)	105.3 (66-154.8)	-0.36 (-0.46--0.27)	-0.57 (-0.68--0.46)

Nigeria	3058.5 (2090.1-4335)	7.7 (5.3-11)	83997.1 (58207.4-119351.7)	183.4 (127.7-259.8)	4870.2 (3271.1-6936.2)	5.7 (3.9-7.9)	146341.4 (98233.2-205610.7)	141.4 (95.4-199.5)	-1.03 (-1.23--0.82)	-0.9 (-1.14--0.65)
Niue	1 (0.8-1.4)	48.1 (35.1-65.6)	29.2 (21.2-39.3)	1411.9 (1018-1895.9)	1 (0.7-1.3)	45.2 (32.9-62.3)	27.7 (20-38.1)	1292.2 (929.1-1787.7)	-0.42 (-0.52--0.32)	-0.53 (-0.64--0.42)
North Macedonia	1691.1 (1326.2-2084)	97.3 (75.8-121.6)	42017.9 (33855.4-50448.7)	2225.5 (1776.3-2693.6)	1913.3 (1422.4-2521.4)	72.6 (52.7-96.4)	42253.2 (31836.1-54531)	1385 (1048-1793.2)	-1.33 (-1.8--0.86)	-1.9 (-2.25--1.54)
Northern Mariana Islands	6.9 (5-8.9)	33.1 (24.7-42.2)	265.9 (193.8-347.8)	959.5 (720.2-1225.7)	17 (13.8-20.8)	31.6 (25.1-39.4)	555.1 (444.2-674.6)	915.7 (735.2-1117.5)	0.02 (-0.14-0.18)	0.06 (-0.11-0.24)
Norway	3217.2 (2577.9-3853.3)	47 (38.5-55.5)	70436 (59135.5-82039.4)	1139.2 (971.6-1307.7)	604.2 (458.5-778.4)	5.8 (4.5-7.3)	13528.1 (10834-16539.1)	148.3 (120.6-179)	-7.16 (-7.39--6.93)	-6.88 (-7.04--6.72)
Oman	357 (254.5-491.9)	52.8 (37.4-74)	10978.9 (7901.5-15143.1)	1359.8 (965.1-1889.5)	457.4 (330.1-619.5)	24.2 (17.2-33.2)	15009.7 (10929.9-20375.8)	568.1 (408.9-768)	-2.18 (-2.31--2.05)	-2.54 (-2.71--2.38)
Pakistan	21606.4 (15306.1-28071.8)	40.4 (28.3-52.9)	609546 (439734.7-779325.2)	1024.9 (737-1316.6)	42503.4 (31321.8-55882.6)	35.9 (26.4-47.4)	1306174.1 (956494.2-1723822.5)	927.1 (688.9-1218.3)	-0.56 (-0.81--0.31)	-0.52 (-0.78--0.25)
Palau	4.9 (3.6-6.4)	49.3 (36.3-65.2)	158.3 (115.7-207.3)	1428.5 (1054.5-1872)	9.5 (6.9-12.7)	41.8 (30.5-56.7)	314.6 (228.4-415.2)	1247.3 (907.2-1658.3)	-0.5 (-0.56--0.44)	-0.42 (-0.49--0.35)
Palestine	698.9 (507.2-909.4)	90 (65.2-	17393.8 (12996.3-	1989 (1473.2-	1115.3 (841.1-	50.6 (37.7-	30803.7 (23675.4-	1127.2 (854.4-	-2.13 (-2.39--	-2.08 (-2.3--

		117.7)	22591.4)	2581.7)	1416.9)	65.6)	38617.5)	1427.9)	1.87)	1.86)
Panama	251.2 (196-316)	17.8 (13.8-22.6)	5921.6 (4814.5-7239.4)	392.8 (316.3-482.5)	300.2 (212.3-415.2)	6.7 (4.7-9.2)	7119.2 (5169.2-9699.4)	160.4 (116.5-218.2)	-3.32 (-3.57--3.06)	-3.03 (-3.29--2.76)
Papua New Guinea	566.5 (384.2-807.1)	29.2 (19.8-41.8)	19976 (13405.2-28611.1)	877.7 (595.7-1241.6)	1643.1 (1126.7-2295.5)	29.2 (19.8-41.3)	58868.1 (40444.9-82391.9)	867.3 (596.3-1205.3)	-0.05 (-0.15-0.05)	-0.08 (-0.18-0.02)
Paraguay	815.1 (642.1-1027.2)	39.4 (30.7-49.9)	18975.4 (15196.5-23717.1)	847.3 (679.8-1058.5)	1242.4 (847.4-1646)	22.3 (15.2-29.7)	29788.7 (21069.3-39768.2)	503.5 (353.4-671.8)	-1.68 (-1.92--1.45)	-1.63 (-1.81--1.45)
Peru	1020.1 (769.1-1268)	8.8 (6.6-11.1)	27252.1 (21124.1-33565.1)	214.1 (165.4-263.9)	1624.8 (1132.2-2285.8)	4.8 (3.3-6.8)	43006.5 (30681.7-59143.7)	123.7 (87.9-170)	-2.62 (-3.17--2.06)	-2.35 (-2.88--1.82)
Philippines	14479.4 (11949.7-17296)	56.4 (45.2-68.9)	450441.5 (377969.2-531865.8)	1353.8 (1127-1605.9)	29478.3 (23119.8-36084.1)	36.5 (28.6-44.8)	913242.9 (715190-1126255.6)	990.2 (778.2-1208.8)	-1.35 (-1.44--1.26)	-1.05 (-1.13--0.97)
Poland	39479.7 (33380.3-46206.2)	93.1 (78.2-109.3)	1042534.8 (903098-1184362.4)	2415.7 (2090.2-2746)	15462.9 (12291.7-19088.2)	21.3 (17.1-26.2)	358879 (294761.4-435236.4)	535.6 (442.7-647.5)	-5.1 (-5.26--4.93)	-5.12 (-5.32--4.92)
Portugal	4228.2 (3373.5-5237.7)	31.9 (25.2-39.7)	98711.2 (81837.7-117369.4)	741.8 (615.2-880.3)	1447.5 (1109.3-1831.6)	6.2 (4.9-7.6)	34769.1 (28299.1-41878.8)	179 (149.2-212.1)	-5.84 (-6.19--5.49)	-5.1 (-5.47--4.72)
Puerto Rico	790.3 (591.6-1017.9)	22.5 (16.8-29.2)	19417.7 (15145.7-24452.6)	544.3 (424.6-685.9)	531.6 (385.2-725.5)	7.7 (5.7-10.3)	12526.8 (9293-16471.4)	215.3 (160.9-282.6)	-3.92 (-4.21--3.64)	-3.47 (-3.78--3.16)
Qatar	70.2 (52.8-	71.6	2448.1 (1859.4-	1615.4	184.9 (126-	20.9	7275.4 (5071.5-	466.6	-4.64 (-	-4.53 (-

	91.1)	(51.7-93.9)	3150.8)	(1204.4-2082.7)	257.5)	(14-29)	10001.3)	(319-649.5)	5.32--3.96)	5.09--3.96)
Republic of Korea	7787.1 (6166.6-9619.4)	34.2 (26.3-43.5)	213508 (172131.8-256999.3)	732.2 (587.7-894.9)	6910.8 (5120.1-9038.8)	7.6 (5.6-9.9)	154746.8 (121303.4-194157.5)	172.6 (135.8-216.4)	-5.59 (-5.87--5.31)	-5.33 (-5.57--5.09)
Republic of Moldova	2481.5 (1955.4-3052.5)	62.1 (47.9-77.7)	64748.1 (52510.5-77692.9)	1471.9 (1180.3-1780.4)	2851.2 (2288-3485.7)	47.8 (38.5-58.3)	72554.8 (59390.4-87098.3)	1252.4 (1026.8-1497.4)	-1.26 (-1.62--0.9)	-0.82 (-1.21--0.42)
Romania	16007.3 (12796.5-19336)	63.8 (50.5-78.3)	414423.5 (343986.8-484767)	1528.4 (1260.1-1798.2)	10592.6 (8172.8-13571.6)	29 (22.8-36.6)	252813.4 (200839.6-312391.9)	765.9 (618.6-938.8)	-3.35 (-3.66--3.04)	-3.11 (-3.44--2.78)
Russian Federation	119819.4 (101517.5-140094.7)	69.6 (58.4-81.8)	3326829.3 (2890421.8-3778973.8)	1862.3 (1612.3-2119.8)	113759.9 (93965-136199)	48 (39.8-57.3)	2968057.5 (2478730.2-3481662.5)	1300.3 (1088.9-1518.1)	-1.8 (-2.6--0.99)	-1.87 (-2.68--1.06)
Rwanda	610.9 (423.9-881)	25.4 (17.3-36.1)	17225.8 (12054.6-24856.4)	598.1 (419.1-853.1)	821.1 (532.2-1198.8)	16.2 (10.2-23.7)	21819.2 (14391.5-31708.3)	350 (231.7-504.8)	-2.33 (-2.68--1.97)	-2.71 (-3.09--2.33)
Saint Kitts and Nevis	9.8 (7.3-12.6)	27.1 (20.4-34.8)	225.3 (175.4-282.8)	651.4 (509.9-814)	6 (4.4-8.2)	9.5 (7-12.9)	159.2 (116.9-217.2)	220.4 (162.7-297.6)	-3.63 (-3.85--3.42)	-3.93 (-4.21--3.65)
Saint Lucia	18.5 (14.5-23.4)	23.1 (17.8-29.6)	465.5 (376.1-569.3)	544.4 (438.2-667.8)	15.4 (11.1-20.3)	6.5 (4.7-8.6)	385.8 (289.2-499.3)	159.3 (119.6-206.1)	-4.68 (-5.02--4.35)	-4.43 (-4.69--4.16)
Saint Vincent and the Grenadines	13.3 (10.5-16.4)	19.5 (15.3-24.2)	347.5 (281.5-419)	495.5 (400-595.1)	15.3 (11.6-19.5)	11.2 (8.5-14.4)	387.7 (301.5-486)	273.8 (212.1-344.1)	-2.04 (-2.22--1.85)	-2.16 (-2.32--2)

Samoa	46.7 (35.7-60)	56.2 (42.4-72.4)	1416.7 (1078.9-1811.9)	1541.2 (1182.1-1972.6)	85.7 (63.9-110)	59.9 (44.5-77.2)	2621.7 (1971.2-3397.7)	1667.8 (1244.6-2149)	0.15 (0.05-0.26)	0.22 (0.13-0.31)
San Marino	7 (5.1-9.3)	19.5 (14.4-25.8)	149.7 (113.2-192.8)	443.9 (340.4-568.7)	3.9 (2.4-5.9)	4.7 (2.8-7.2)	82.7 (53.3-121.8)	121.6 (77.2-178.9)	-3.99 (-4.32--3.66)	-3.74 (-4.01--3.48)
Sao Tome and Principe	3.4 (2.5-4.6)	5.4 (4-7.2)	100.8 (74.9-133.3)	150.7 (112.2-199.8)	7.2 (5.1-10.1)	6.4 (4.6-8.6)	235.4 (164.9-330.6)	178.5 (126-246.4)	0.4 (0.07-0.73)	0.35 (0-0.7)
Saudi Arabia	2543.8 (1774.5-3505.4)	42.2 (29.7-57.6)	82013.2 (57425-113136.4)	1127.9 (787.6-1560.5)	9272.4 (6622-12341.9)	37.2 (26.1-49.1)	352798.4 (251508.1-473688.3)	1073.4 (769.4-1418.7)	-0.32 (-0.54--0.1)	0.09 (-0.17-0.35)
Senegal	654.1 (468.3-896.3)	21.4 (15.3-29.7)	18966.5 (13665-25603.5)	550.7 (396.7-745.6)	1009.6 (671.1-1456.7)	14 (9.2-20.5)	28651.9 (19503.5-40093.1)	344.3 (232.7-487.7)	-1.73 (-1.87--1.6)	-1.85 (-1.99--1.72)
Serbia	8157.4 (6417.7-10025.7)	85.6 (65.6-107)	202642.5 (165460.7-244988.2)	1869.7 (1502.7-2269.1)	7180.3 (5439.6-9451.5)	43 (32.7-56.2)	152130.9 (119198.1-193808.1)	970 (766.6-1224.3)	-2.78 (-3.2--2.37)	-2.64 (-3.05--2.23)
Seychelles	24.3 (19.1-29.8)	43.4 (34-53.3)	637.7 (512.8-763.6)	1140.3 (919.5-1363.4)	25.4 (19.8-32.3)	22.8 (17.5-29.5)	710.2 (564.8-890.8)	581.8 (461.6-729.8)	-2.1 (-2.27--1.92)	-2.25 (-2.42--2.07)
Sierra Leone	403.5 (286.5-534)	20.7 (14.7-27.2)	11299.5 (8082.6-14785.8)	530.8 (378.5-691.3)	544.9 (375.1-765.9)	14.9 (10.2-21)	16624.1 (11255.7-23446.2)	389.6 (268.9-545.2)	-0.92 (-1.15--0.69)	-0.81 (-1.04--0.58)
Singapore	658.4 (533-792.9)	29.7 (23.6-	19705.3 (15904-	801.4 (646.9-	507.3 (411.5-626.6)	5.8 (4.7-	14781.7 (12109.8-	168.5 (138.3-	-5.28 (-5.41--	-5 (-5.12--

		36.2)	23479.2)	958.3)		7.2)	17974.8)	205.3)	5.15)	4.88)
Slovakia	4673 (3720.1-5751.7)	79.9 (63.6-98.2)	119085.3 (98158.8-141129.6)	2026.7 (1668.4-2400.3)	3039.3 (2303.8-3985.4)	32 (24.3-42)	69269.6 (53982.5-88239.1)	753.4 (589.3-959.7)	-2.85 (-2.95--2.75)	-3.11 (-3.21--3.01)
Slovenia	860.5 (689.7-1040.8)	35.2 (28.1-42.5)	21878.4 (18178.4-25887.6)	894.8 (744-1056)	434.8 (324-569.4)	9.3 (7.1-11.9)	9715 (7666-12114.7)	239.2 (190.4-297.7)	-4.41 (-4.53--4.29)	-4.39 (-4.5--4.27)
Solomon Islands	104.8 (69.1-142.1)	76.3 (52.9-101.4)	3426.6 (2180.1-4683.3)	2131.8 (1414.8-2879.5)	258.6 (189.7-344.5)	71.5 (52.5-94.6)	8723.5 (6422.4-11655.5)	2045.5 (1508.8-2715.8)	-0.06 (-0.3-0.19)	0.02 (-0.22-0.27)
Somalia	242.2 (143.1-389.7)	10.6 (6.5-17.1)	8331.4 (5101.7-13224.5)	284.5 (173.5-450.7)	495.6 (282-785.4)	8.2 (4.7-12.9)	17345.5 (10008.8-27175.3)	223.4 (132.4-348.9)	-0.84 (-0.88--0.8)	-0.82 (-0.87--0.78)
South Africa	4762.6 (3756.2-5807.7)	23.7 (18.2-29.4)	148081 (121038.3-176053.9)	646.9 (523.1-775.4)	5747.8 (4587-7009.1)	12.9 (10.2-15.9)	172782.6 (141057.7-208594)	344.4 (278.2-416.2)	-2.14 (-2.45--1.82)	-2.19 (-2.46--1.91)
South Sudan	295 (192.6-443.7)	12.1 (7.9-18.2)	8475.2 (5618.8-12643.7)	315.1 (209-468.6)	385.8 (245.5-576.1)	10.4 (6.7-15.4)	12210.1 (7797.8-17983.8)	271 (176.7-399.3)	-0.67 (-0.83--0.51)	-0.68 (-0.87--0.49)
Spain	17699.9 (14082.7-21342.4)	33.3 (26.6-40.1)	392186.7 (327604.2-457275.7)	762.1 (640.7-884.1)	7209.7 (5574.7-9176.9)	7.2 (5.7-8.8)	167573.9 (136617.2-204219.9)	197.6 (163-236.9)	-5.17 (-5.31--5.03)	-4.56 (-4.68--4.44)
Sri Lanka	3551.9 (2863.5-4384.4)	38.3 (29.7-48.2)	96555.4 (80069.8-116768.4)	864.6 (705.1-1065.2)	3908.3 (2431.5-5735.4)	15.7 (9.7-23)	99300.7 (61469-143696.3)	376.3 (234.5-543.2)	-2.63 (-2.76--2.5)	-2.63 (-2.77--2.49)
Sudan	6807.7	76.1	202481.7	1993.8	9523.1	50.7	283254.2	1249.2	-1.53 (-	-1.72 (-

	(4914.7-9170.7)	(54.3-102.7)	(146774.1-270620.7)	(1440.6-2672.4)	(6541.6-13519.5)	(35.3-70.8)	(186172.4-404536.6)	(860.4-1766.3)	1.61--1.44)	1.81--1.62)
Suriname	107.2 (86.5-133)	42.1 (33.6-52.7)	3079.3 (2533.9-3750.3)	1125.8 (923.2-1376.6)	117.4 (82.9-161.4)	18.3 (12.9-25.3)	3418.3 (2450.3-4618.5)	513.7 (368.5-696.1)	-2.89 (-3.14--2.63)	-2.77 (-3.03--2.51)
Sweden	6627.7 (5202.7-8114.1)	41.9 (33.5-50.9)	133377.1 (107744.2-159516.8)	929.8 (765.8-1102.9)	2309.7 (1674.8-3032)	9.6 (7.3-12.3)	44217.2 (33836.6-56043)	214.9 (168.6-270)	-4.64 (-4.75--4.52)	-4.57 (-4.63--4.51)
Switzerland	3977.5 (3131.4-4856.8)	37.2 (29.8-44.9)	82501.6 (68108.1-97584.8)	831 (694.6-972.9)	1506.6 (1126.9-1961.3)	7.2 (5.6-9.2)	27655.2 (22180.7-34824.8)	157 (128.7-194)	-5.34 (-5.43--5.25)	-5.44 (-5.55--5.33)
Syrian Arab Republic	5546.1 (4112.9-7032.6)	109.4 (80.7-138.9)	164077 (123763.5-208828.4)	2793.6 (2088.7-3551.1)	8826.8 (6071.5-11863.8)	74.6 (51.5-100.6)	247040.2 (169608-334246.9)	1758.7 (1216.9-2361.5)	-1.67 (-1.83--1.51)	-1.92 (-2.11--1.73)
Taiwan (Province of China)	3545.3 (2906.9-4203.2)	26.2 (20.9-31.8)	94567.1 (79512.7-110650)	599.4 (496.3-707.6)	3694.7 (2893.1-4645.8)	8.7 (6.8-10.9)	94974.3 (76601.5-115800.6)	237.5 (192.2-288.2)	-3.47 (-3.7--3.24)	-2.86 (-3.08--2.65)
Tajikistan	2036.6 (1585.3-2513.4)	78.4 (60.7-97.8)	53221.4 (42408-64514.3)	1882.6 (1488.4-2290.8)	1766.8 (1271.6-2430.6)	35.6 (25.5-48.8)	49366.8 (35459.8-66523.8)	799.9 (579.1-1094.9)	-2.72 (-3.05--2.38)	-3.03 (-3.33--2.74)
Thailand	8200.6 (6346.7-10184.8)	25.8 (19.4-32.5)	234304.7 (184175.5-287548.5)	621.3 (484-763.1)	12185.1 (8681.4-16731.2)	11.4 (8.2-15.6)	328512.1 (241783.8-440238.7)	318.1 (235.9-424.3)	-3.29 (-3.5--3.07)	-2.79 (-3.01--2.57)
Timor-Leste	82.5 (57.9-110)	33.8 (24-45.4)	2598.2 (1814.4-3402.8)	801.7 (568.2-1061)	301.7 (200.8-420.9)	40 (26.9-55.2)	8015.9 (5374.8-11200.7)	933.9 (630.7-1292.8)	0.81 (0.62-1.01)	0.76 (0.52-1)

Togo	263.9 (190.2-351.7)	23.5 (16.9-31.9)	7752.8 (5617.1-10247.3)	582.5 (423.8-774.8)	646.1 (425.8-914.3)	17.5 (11.6-24.8)	20175.7 (13282.3-28554.9)	455.9 (302.7-640.6)	-1.22 (-1.4--1.03)	-1.03 (-1.24--0.83)
Tokelau	0.6 (0.5-0.8)	48 (34.5-64.7)	17.5 (12.5-23.7)	1329.3 (942.5-1816.2)	0.6 (0.4-0.8)	39.7 (28.6-54.5)	16.4 (11.9-22.3)	1142.1 (828.4-1545.2)	-0.74 (-0.82--0.66)	-0.61 (-0.68--0.53)
Tonga	23.5 (18-30)	44.4 (33.6-57)	681.1 (521.9-854.9)	1166.6 (893.4-1471.5)	31 (23-42.7)	38.9 (28.9-53.4)	853.6 (635.6-1158.4)	1030.1 (769.1-1400.2)	-0.4 (-0.53--0.27)	-0.35 (-0.46--0.25)
Trinidad and Tobago	360.2 (290.6-435.3)	44.9 (35.7-54.8)	10004 (8246.4-11870.4)	1162.6 (951.5-1384)	335.4 (227.7-456.2)	17.6 (11.9-23.8)	9211.3 (6430.4-12367.7)	485 (339.4-652.9)	-3.69 (-4--3.38)	-3.49 (-3.81--3.17)
Tunisia	3265.6 (2480.9-4108.2)	76.4 (57.7-97.7)	82176.3 (63654.6-101534.5)	1648.1 (1267.9-2060.6)	5837.5 (3996-8231.7)	47.3 (32.2-66.3)	137168.8 (94576.7-190579.4)	1029 (717-1430.3)	-1.93 (-2.09--1.77)	-1.89 (-2.03--1.75)
Turkey	25124.4 (19895.5-30229.2)	76.4 (60-92.9)	709692.5 (573552.2-842172.9)	1905.2 (1523.6-2277.8)	27509.4 (19992.8-37237.8)	31 (22.3-42.1)	678888.3 (505338.4-895247.7)	716.7 (529.1-945.7)	-3.23 (-3.51--2.94)	-3.57 (-3.82--3.32)
Turkmenistan	1692.7 (1357-2010.7)	94.9 (75.6-113.9)	47834.1 (39182.7-55864.6)	2374.3 (1939.6-2791.2)	2451 (1763.1-3205.4)	65.3 (47-86.1)	68304.2 (49516-89763.2)	1595.4 (1161-2088.5)	-2.07 (-2.49--1.64)	-2.16 (-2.63--1.69)
Tuvalu	4.5 (3.4-5.7)	66.2 (50.6-85.9)	143.6 (110.8-184.1)	1933.6 (1487.5-2470.2)	6.5 (5-8.4)	63.2 (47.9-82.5)	207.7 (158.9-266.5)	1875.8 (1434.6-2417.9)	-0.1 (-0.15--0.05)	-0.03 (-0.08-0.02)
Uganda	502.1 (346.8-717.2)	8.7 (6-12.5)	14612.2 (10154.8-	217.1 (152.1-	942.2 (654.8-1292.5)	6.8 (4.7-	29411.3 (20754.2-	175.1 (122.7-	-1.85 (-2.37--	-1.75 (-2.28--

			20764.5)	307.8)		9.3)	40201.9)	237.8)	1.33)	1.23)
Ukraine	55329.5 (44138.3-67894.4)	81.6 (64.6-100.8)	1300834.6 (1067603.8-1546438.6)	1863.5 (1528.8-2224)	47553.9 (33924-64233.2)	61.5 (43.8-83)	1115354.6 (781488.8-1522748.7)	1518.4 (1061.4-2070.3)	-1.56 (-2.23--0.88)	-1.34 (-2.01--0.67)
United Arab Emirates	263.8 (190.1-359.1)	59.2 (41.7-79.4)	9441.3 (6760.7-12754.1)	1461.7 (1056.2-1964.5)	822.6 (578.4-1104.6)	30.5 (20.6-41.8)	32277.3 (23221.2-42922.1)	616.6 (433.5-824.6)	-1 (-1.44--0.55)	-2.06 (-2.44--1.67)
United Kingdom	57016.3 (47379.1-67415.6)	62.8 (52.7-73.5)	1251219.7 (1068677.1-1447932.8)	1477.6 (1274.4-1696.3)	12913.7 (10240-15982)	9.8 (7.9-11.9)	282889.3 (233361.4-341668.7)	244.5 (205.2-292.2)	-6.46 (-6.71--6.21)	-6.2 (-6.45--5.94)
United Republic of Tanzania	1531.5 (1113-2017)	15.4 (11.1-20.5)	45034.3 (33386.4-58447)	391.3 (288.3-508.7)	3956.3 (2599-5668.7)	16.1 (10.4-23)	119637.4 (80199.8-168594.5)	415.3 (276.6-585.4)	-0.25 (-0.4--0.09)	-0.19 (-0.34--0.03)
United States of America	146592.7 (121868.9-173567.7)	46.3 (38.7-54.4)	3491639 (2992607.5-4029417.5)	1162.1 (1000.7-1332.1)	87575.4 (69744.4-106104.6)	15.2 (12.3-18.3)	2127382.4 (1755224.6-2522056.8)	406.3 (338.3-478.2)	-3.97 (-4.18--3.77)	-3.72 (-3.91--3.53)
United States Virgin Islands	21.5 (15-30)	26.7 (18.2-37.6)	621 (444.3-864.4)	671 (471.9-933.3)	21.5 (14.3-30.2)	12.5 (8.4-17.6)	501.8 (347.2-710)	315.6 (218.6-443.2)	-2.4 (-2.54--2.25)	-2.35 (-2.5--2.2)
Uruguay	1402.1 (1118.3-1705.9)	36.6 (29.3-44.4)	35569.4 (29695.6-42098.1)	954.6 (802.6-1125.8)	731.3 (575.5-897.9)	13.4 (10.8-16.3)	17923.9 (14718.1-21389.3)	368 (304.7-436.7)	-3.52 (-3.62--3.41)	-3.35 (-3.47--3.24)
Uzbekistan	4327.2 (3285-5550.8)	38.4 (28.9-49.8)	123204.6 (96742.2-152834)	1021.2 (794.8-1277.6)	11158.6 (8387.9-14461.4)	46.4 (34.4-60.4)	316943 (243871.9-403051.9)	1119.2 (847.9-1435)	0.64 (0.04-1.24)	0.25 (-0.32-0.82)
Vanuatu	40 (29.9-52.4)	59.5	1379.2 (1019.6-	1766	90 (68.3-	47.3	3160.2 (2373.9-	1448.3	-0.99 (-	-0.88 (-

		(44.4-77)	1834.1)	(1325.1-2311.3)	114.1)	(36.2-59.8)	4049.2)	(1101.6-1842.5)	1.1--0.88)	0.99--0.78)
Venezuela (Bolivarian Republic of)	3171.3 (2585.5-3748.8)	33.6 (27-40.2)	89869.6 (74229.6-105189.6)	852.6 (703-1000.4)	5554.2 (3885-7493.8)	18.6 (13-25.2)	149194.2 (104712.7-198863.2)	481.5 (338.4-641.7)	-2.41 (-2.67--2.15)	-2.39 (-2.65--2.14)
Viet Nam	10888.8 (7823.9-15050.1)	29.7 (21.2-41.5)	262727.8 (191654.6-357358.2)	659.2 (482.3-896.1)	26777.8 (19348.1-34819.9)	29.7 (21.5-38.5)	679080.4 (495923.2-885131.1)	673.4 (492.9-873.4)	0.1 (-0.02-0.22)	0.29 (0.16-0.42)
Yemen	4629.2 (3303.7-6304.4)	96.7 (69.1-131)	142845.8 (101780.1-194207.5)	2565.6 (1832-3489.9)	9919.3 (6827.4-13585)	74.6 (51.3-102.7)	294039.6 (203277.2-404187.8)	1828.5 (1260.6-2491.5)	-1.08 (-1.17--0.98)	-1.36 (-1.46--1.26)
Zambia	301.3 (217-414.8)	12.5 (8.6-17.4)	8522.6 (6238.6-11440.4)	295.1 (216-401.6)	823.4 (550.4-1137.7)	13.3 (8.8-18.5)	24773.2 (16511.8-34314.2)	321.8 (218-440.9)	-0.05 (-0.19-0.09)	-0.01 (-0.17-0.15)
Zimbabwe	630.8 (467.4-833.6)	19.2 (13.8-26.4)	16436.1 (12570.9-21363.4)	416.9 (314-549)	1451.3 (1018-1996.2)	24.2 (16.9-33)	42883.5 (29959.7-58995.4)	579.8 (411-796.9)	1.16 (0.59-1.74)	1.38 (0.79-1.97)

*The all-age mortality is equivalent to the crude mortality rate.

*The numbers in parentheses in the table represent the 95% confidence interval.

Abbreviation: CVD, cardiovascular disease. EAPC, estimated annual percentage change. ASMR, age-standardized mortality rate, ASDR, age-standardized death Rate.

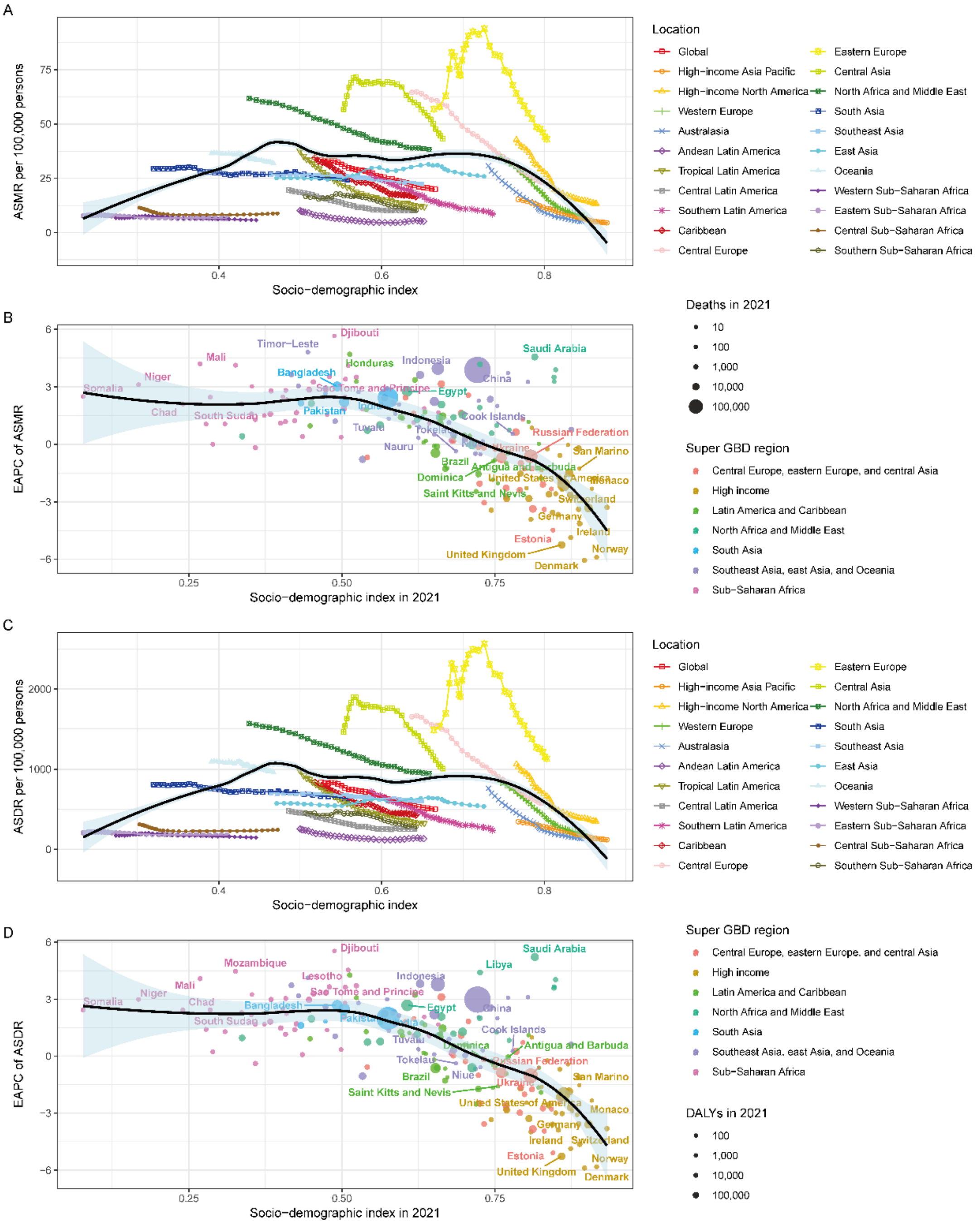

Figure S1. The change of ASMR and ASDR related to ischemic heart disease attributable to tobacco with SDI changes (A) ASMR and SDI by GBD location from 1990 to 2021, (B) EAPC in ASMR and SDI in 2021 by Super GBD region, (C) ASDR and SDI by GBD location from 1990 to 2021, (D) EAPC in ADMR and SDI in 2021 by Super GBD region.

Abbreviation: SDI, socio-demographic index. EAPC, estimated annual percentage change. ASMR, age-standardized mortality rate, ASDR = age-standardized DALYs rate, DALYs disability-adjusted life years.

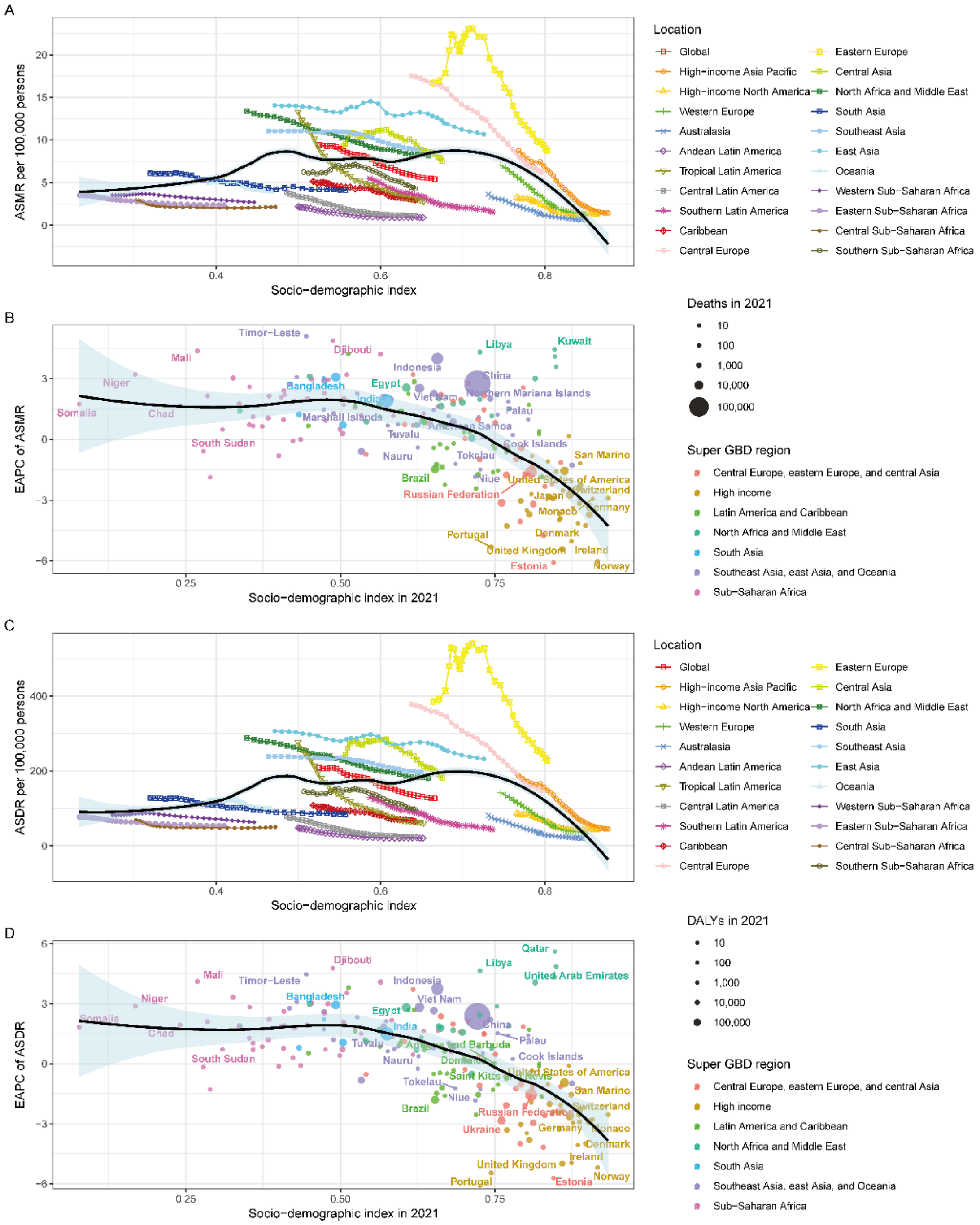

Figure S2. The change of ASMR related to ischemic stroke attributable to tobacco with SDI changes (A) ASMR and SDI by GBD location from 1990 to 2021, (B) EAPC in ASMR and SDI in 2021 by Super GBD region, (C) ASDR and SDI by GBD location from 1990 to 2021, (D) EAPC in ADMR and SDI in 2021 by Super GBD region.

Abbreviation: SDI, socio-demographic index. EAPC, estimated annual percentage change. ASMR, age-standardized mortality rate, ASDR = age-standardized DALYs rate, ,DALYs disability-adjusted life years.

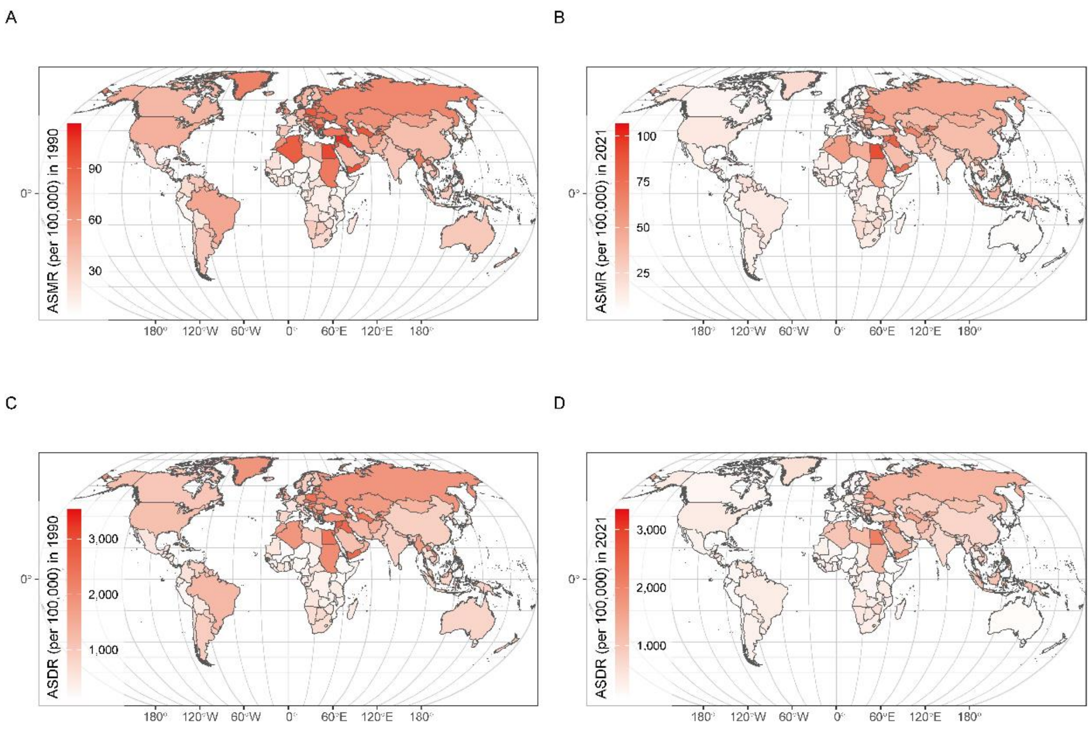

Figure S3. World map of ASMR and ASDR to CVD attributable to tobacco in 1990 and 2021, and the change of ASMR and ASDR globally from 1990 to 2021. (A) ASMR of CVD attributable to tobacco in 1990. (B) ASMR of CVD attributable to tobacco in 2021. (C) ASDR of CVD attributable to tobacco in 1990. (D) ASDR of CVD attributable to tobacco in 2021.

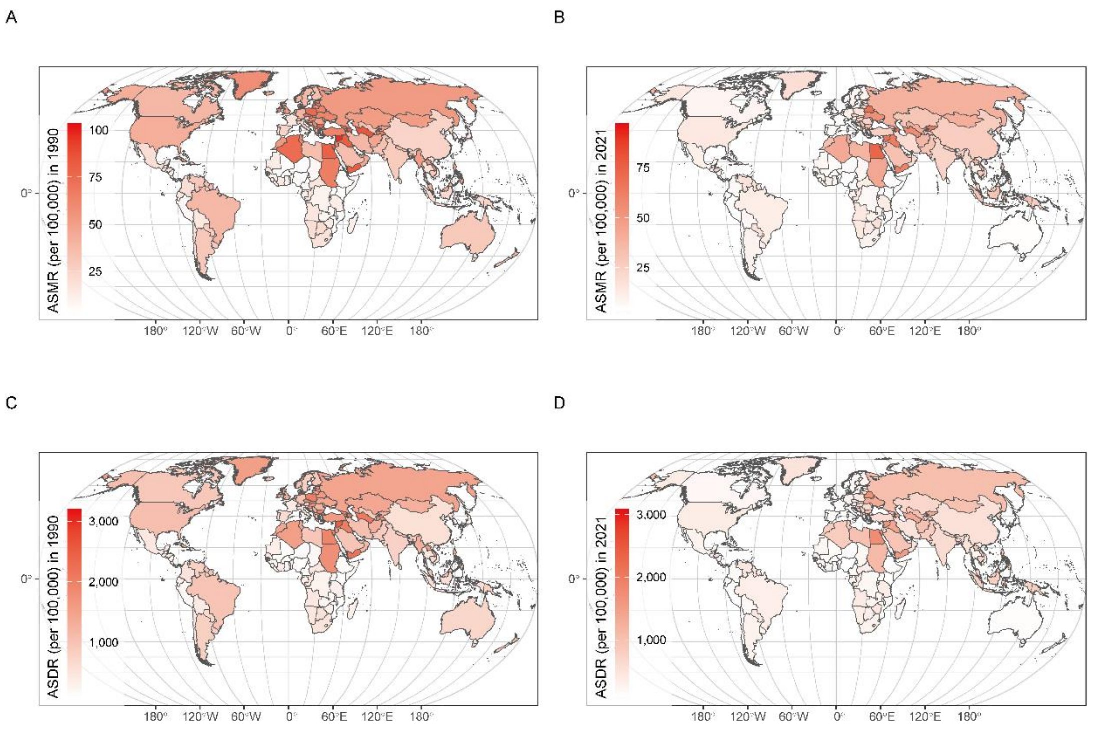

Figure S4. World map of ASMR and ASDR of ischemic heart disease to tobacco in 1990 and 2021, and the change of ASMR and ASDR globally from 1990 to 2021. (A) ASMR of ischemic heart disease attributable to tobacco in 1990. (B) ASMR of ischemic heart disease attributable to tobacco in 2021. (C) ASDR of ischemic heart disease attributable to tobacco in 1990. (D) ASDR of ischemic heart disease attributable to tobacco in 2021.

Abbreviation: ASMR, age-standardized mortality rate. ASDR, age-standardized DALYs rate, DALYs disability-adjusted life years.

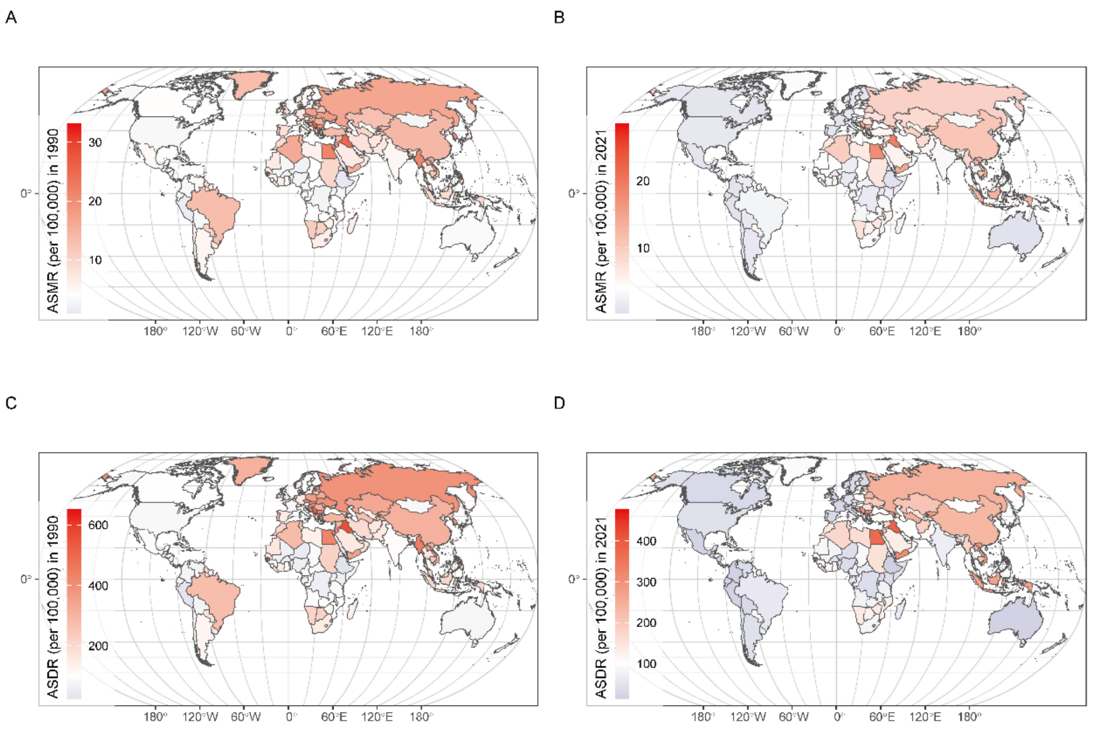

Figure S5. World map of ASMR and ASDR of ischemic stroke disease to tobacco in 1990 and 2021, and the change of ASMR and ASDR globally from 1990 to 2021. (A) ASMR of ischemic stroke attributable to tobacco in 1990. (B) ASMR of ischemic stroke attributable to tobacco in 2021. (C) ASDR of ischemic stroke attributable to tobacco in 1990. (D) ASDR of ischemic stroke attributable to tobacco in 2021.

Abbreviation: ASMR, age-standardized mortality rate. ASDR, age-standardized DALYs rate, DALYs disability-adjusted life years.

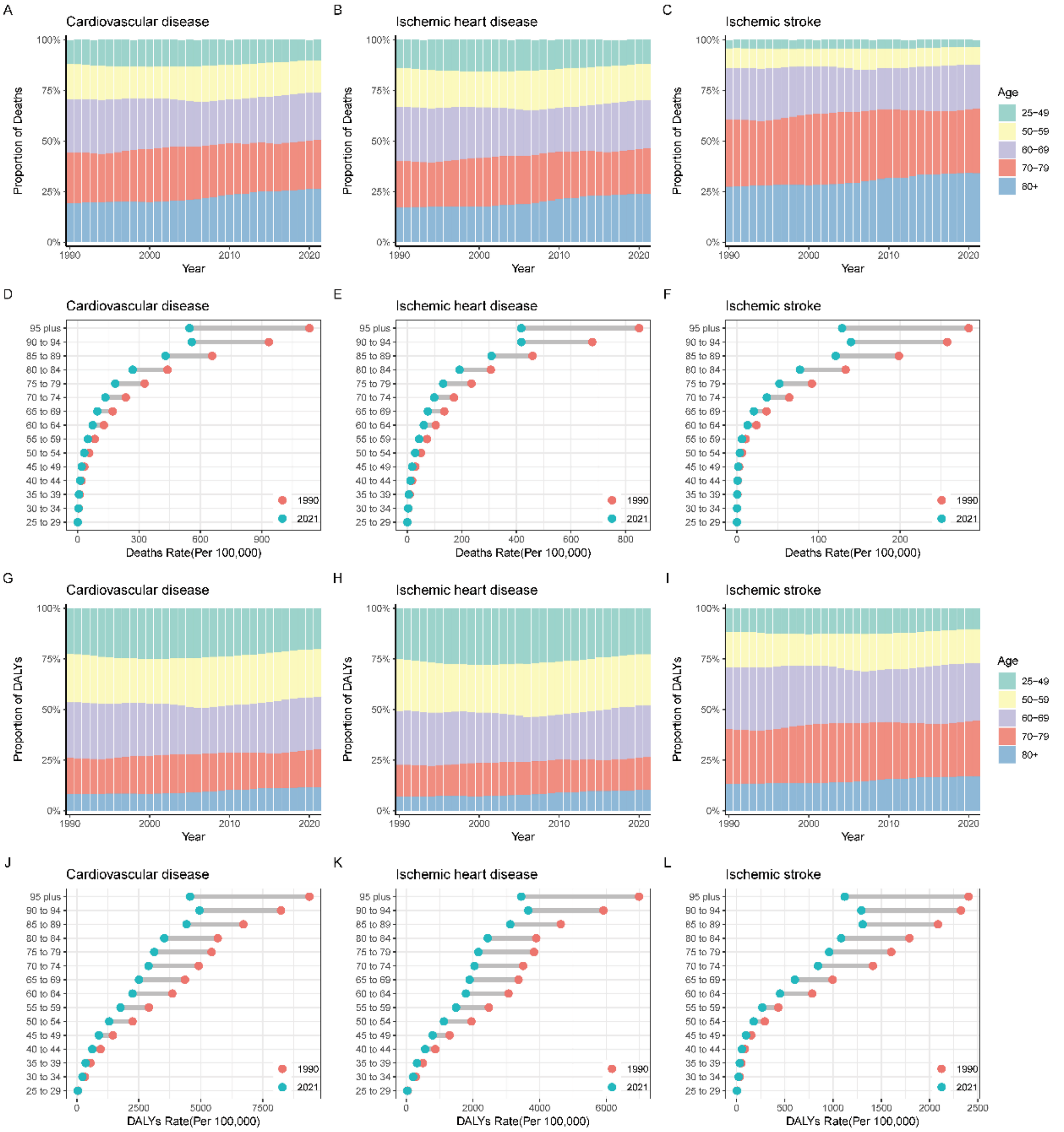

Figure S6. The temporal change of the mortality rate and DALYs attributed to CVD attributable to tobacco across age groups in global from 1990 to 2021. (A-C) The relative proportion of CVD related to mortality, (D-F) The temporal changes in the mortality rate of CVD, (G-I) The relative proportion of CVD related to DALYs, (J-L), The temporal changes in the DALYs rate of CVD. Abbreviations: CVD, cardiovascular disease. DALYs, disability-adjusted life years

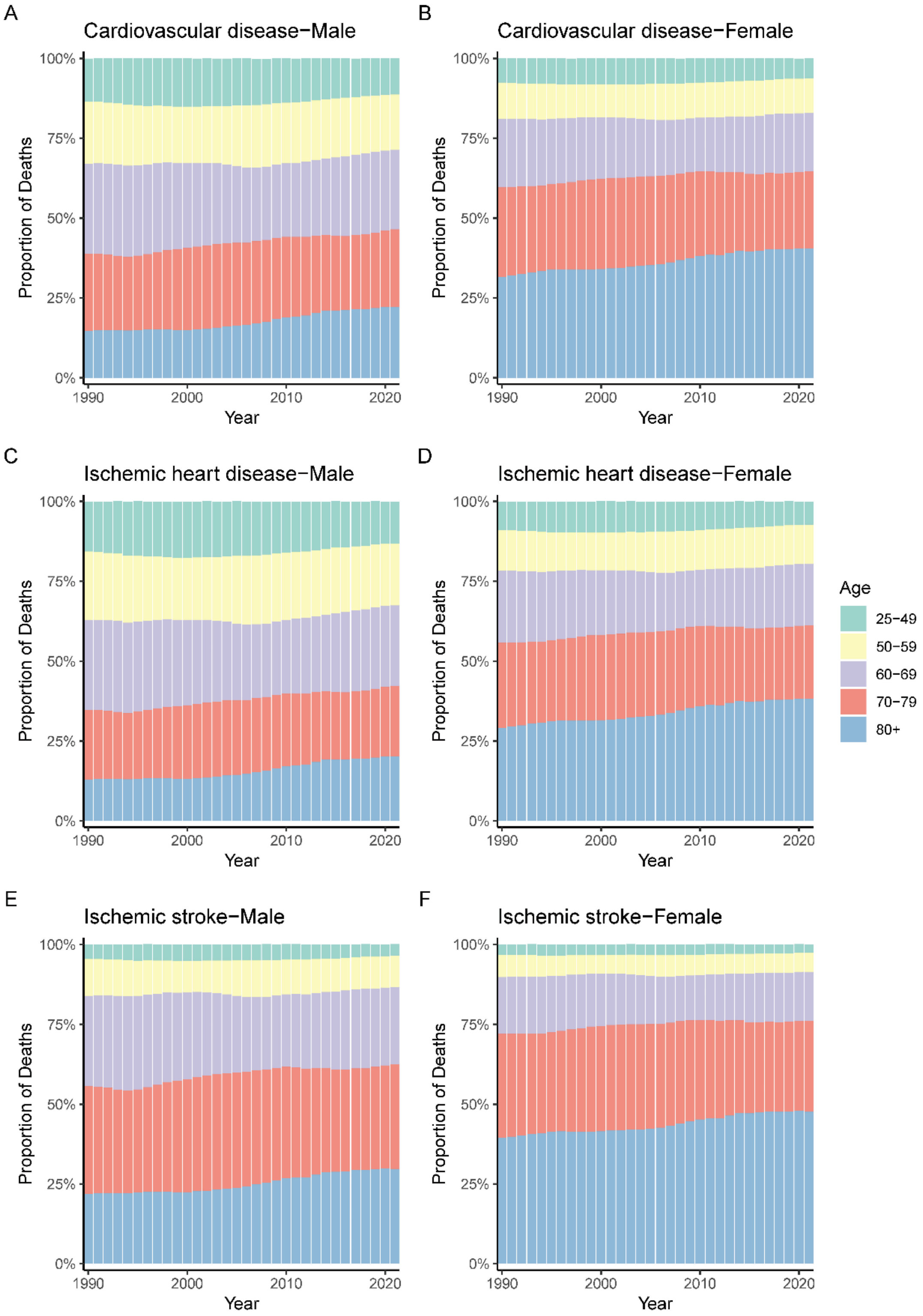

Figure S7. The temporal change in the sex-specific relative proportion of CVD deaths attributable to tobacco across age groups in global from 1990 to 2021.

Abbreviation: CVD, cardiovascular disease.

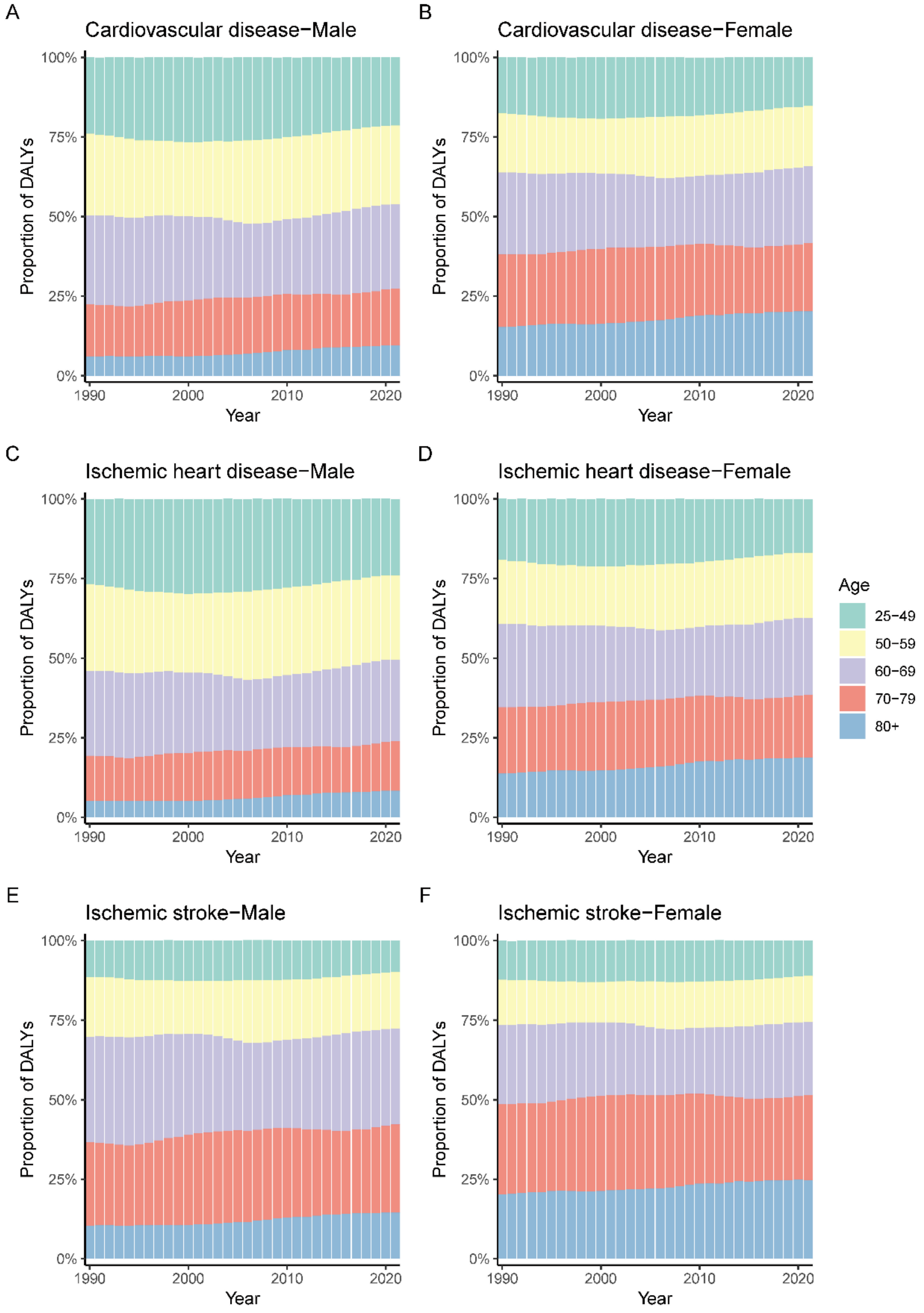

Figure S8. The temporal change in the sex-specific relative proportion of CVD DALYs attributable to tobacco across age groups in global from 1990 to 2021.

Abbreviation: CVD, cardiovascular disease, DALYs disability-adjusted life years.

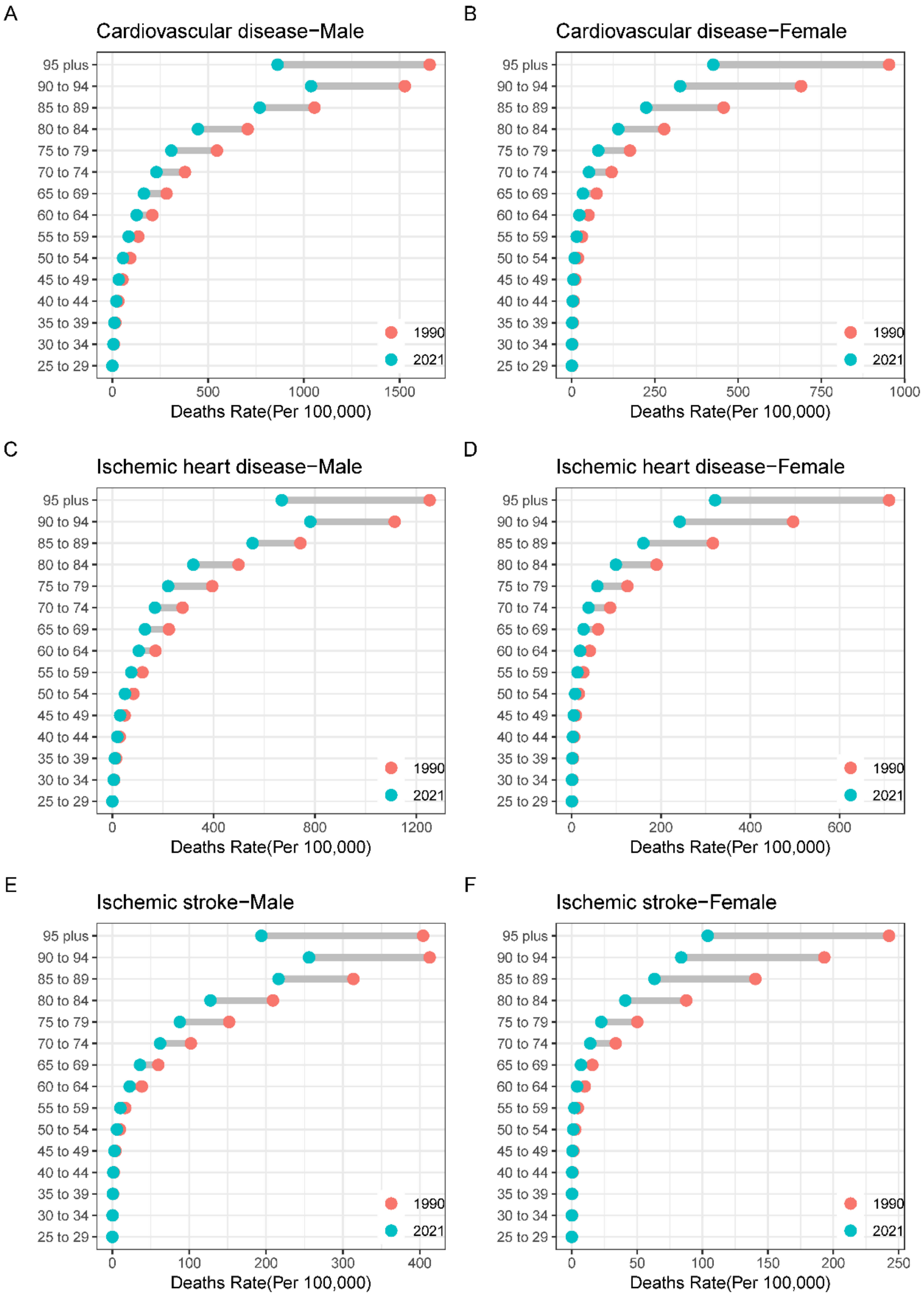

Figure S9. The temporal change in the sex-specific deaths rate for CVD attributable to tobacco across age groups in global from 1990 to 2021.

Abbreviation: CVD, cardiovascular disease.

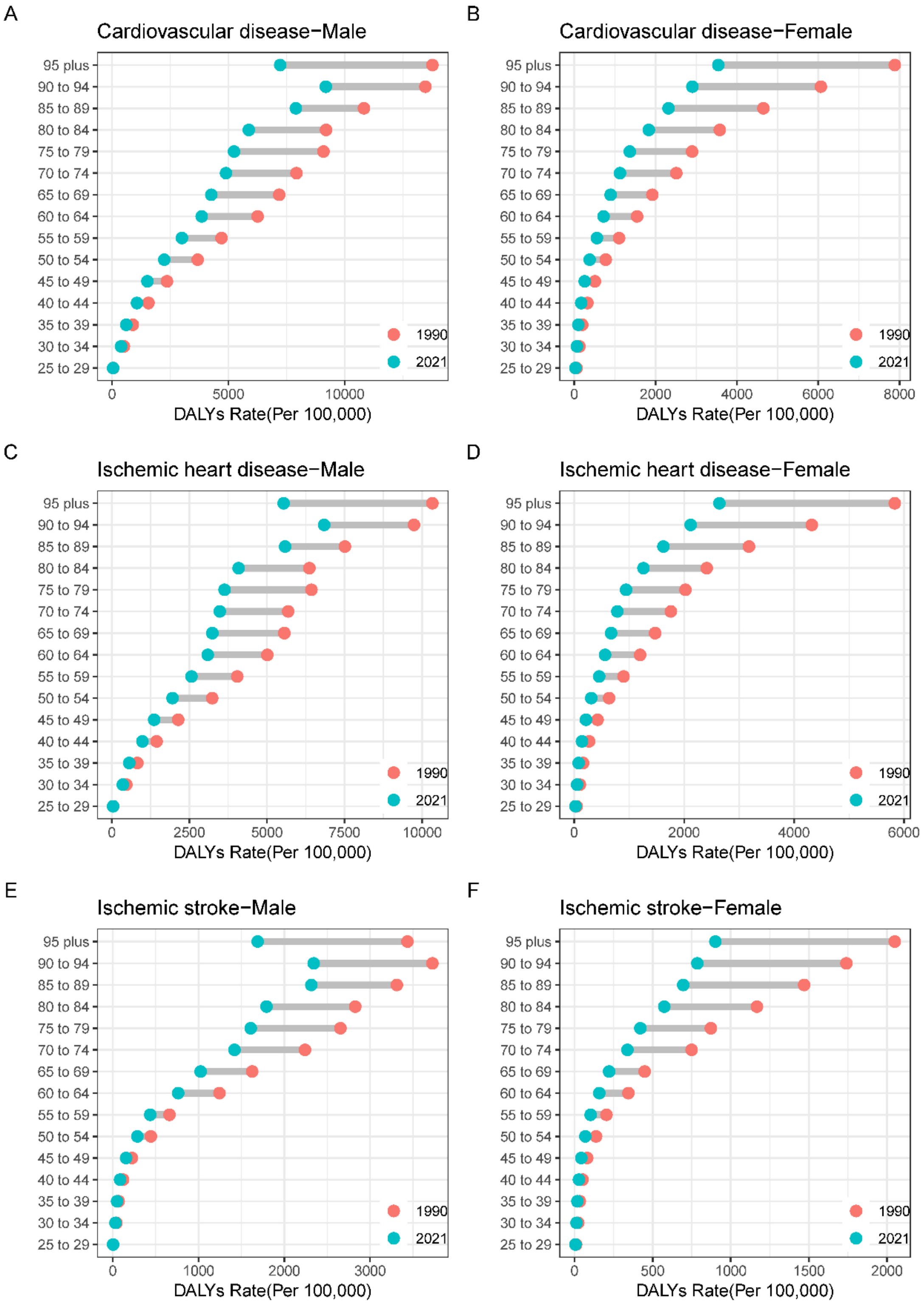

Figure S10. The temporal change in the sex-specific deaths rate for CVD attributable to tobacco across age groups in global from 1990 to 2021.

Abbreviation: CVD, cardiovascular disease, DALYs disability-adjusted life years.

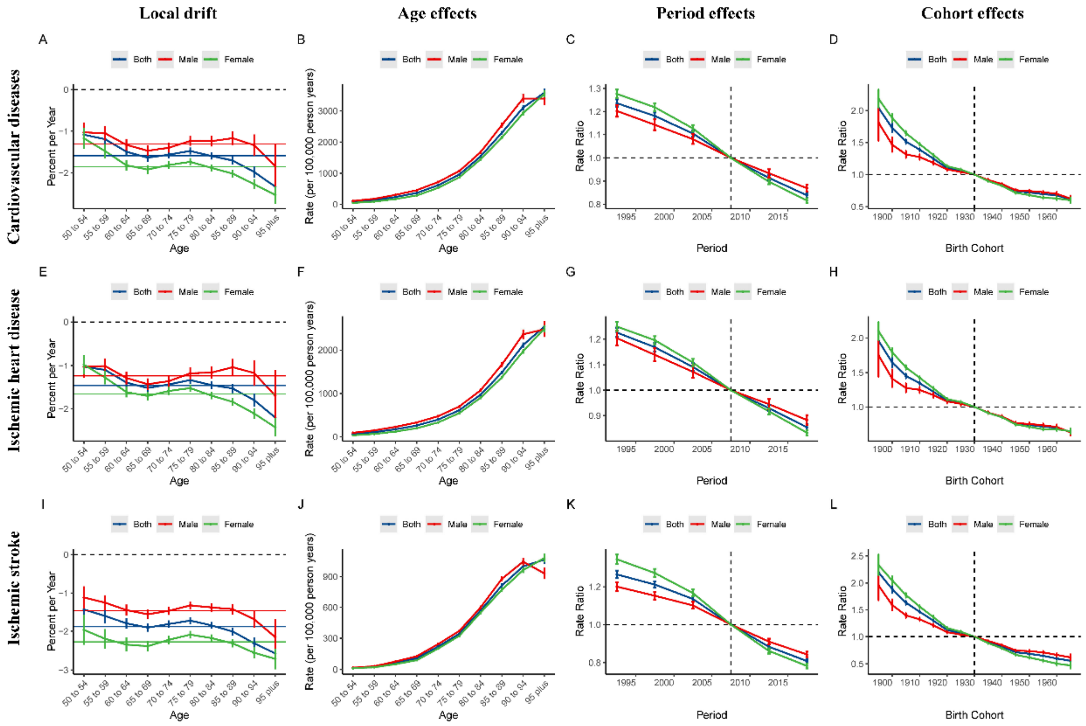

Figure S11. The local drifts, age effects, period effects, and cohort effects of CVD-related mortality attributable to tobacco in global from 1990 to 2021. (A) The local drift analysis of CVD, (B) The age effect of CVD, (C) The period effect of CVD, (D) The cohort effect of CVD, (E) The local drift analysis of ischemic heart disease, (F) The age effect of ischemic heart disease, (G) The period effect of ischemic heart disease, (H) The cohort effect of ischemic heart disease, (I) The local drift analysis of ischemic stroke, (J) The age effect of ischemic stroke, (K) The period effect of ischemic stroke, (L) The cohort effect of ischemic stroke.

Abbreviations: CVD, cardiovascular disease.

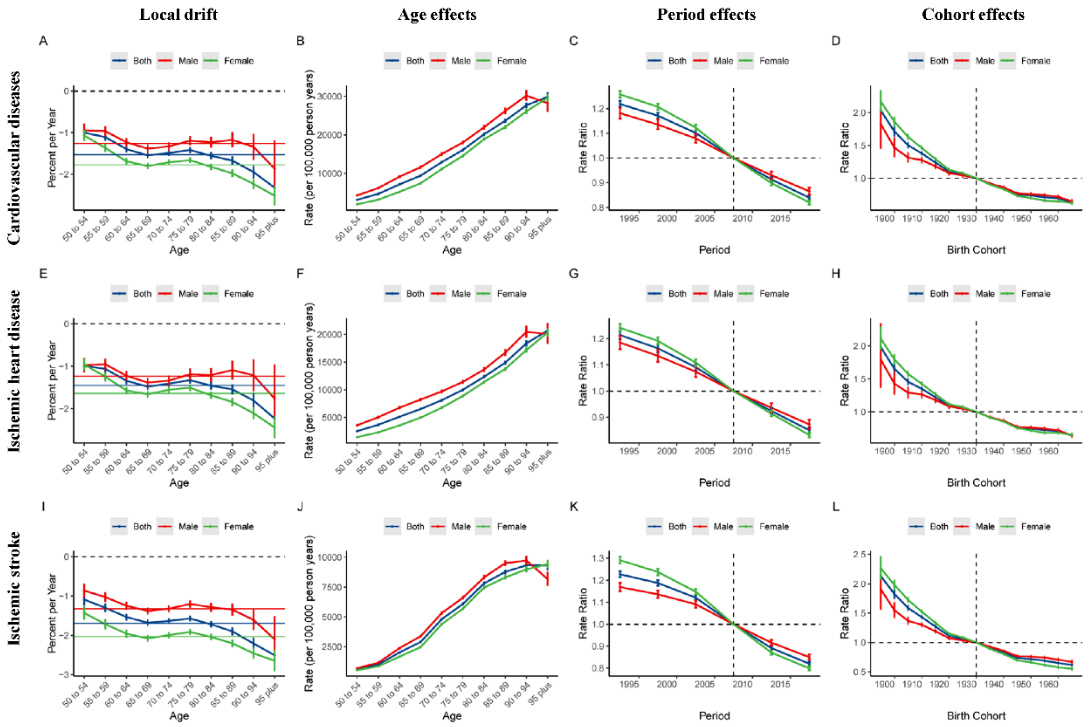

Figure S12. The local drifts, age effects, period effects, and cohort effects of CVD-related DALYs attributable to tobacco in global from 1990 to 2021. (A) The local drift analysis of CVD, (B) The age effect of CVD, (C) The period effect of CVD, (D) The cohort effect of CVD, (E) The local drift analysis of ischemic heart disease, (F) The age effect of ischemic heart disease, (G) The period effect of ischemic heart disease, (H) The cohort effect of ischemic heart disease, (I) The local drift analysis of ischemic stroke, (J) The age effect of ischemic stroke, (K) The period effect of ischemic stroke, (L) The cohort effect of ischemic stroke.

Abbreviations: CVD, cardiovascular disease. DALYs, disability-adjusted life years.

A

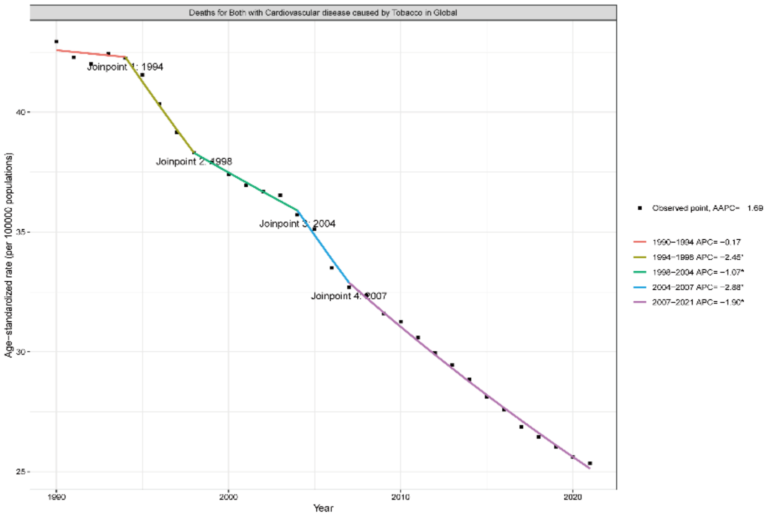

B

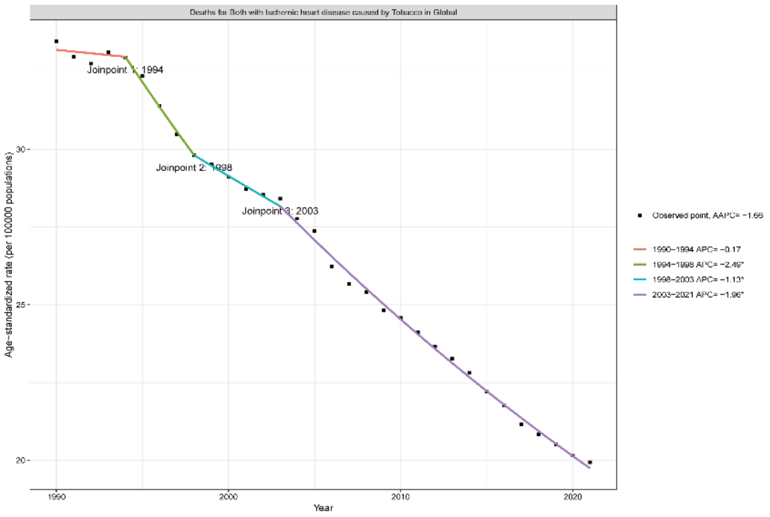

C

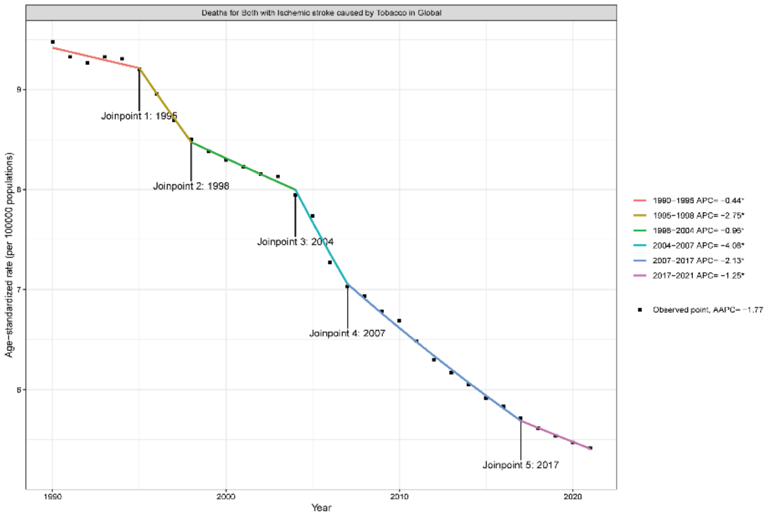

Figure S13. The joinpoint regression analysis of the age -standardized death rate for CVD attributable to tobacco in global from 1990 to 2021. (A) The joinpoint regression analysis of the age -standardized death rate for CVD, (B) The joinpoint regression analysis of the age -standardized death rate for ischemic heart disease, (C) The joinpoint regression analysis of the age -standardized death rate for ischemic stroke. Abbreviations: CVD, cardiovascular disease.

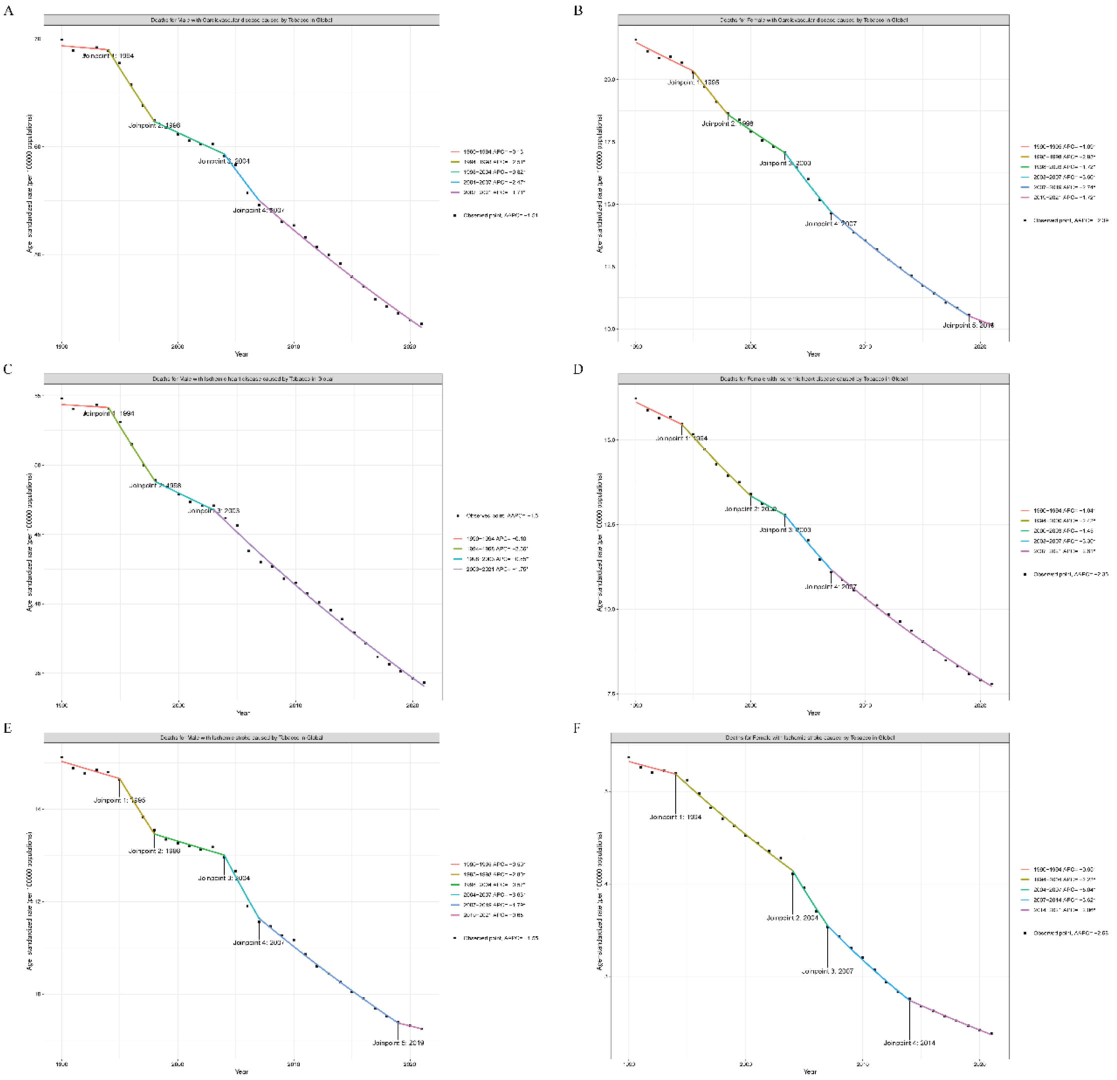

Figure S14. The joinpoint regression analysis of the sex-specific age-standardized death rate for CVD attributable to tobacco in global from 1990 to 2021. (A) The joinpoint regression analysis of the male age -standardized death rate for CVD, (B) The joinpoint regression analysis of the female age -standardized death rate for CVD, (C) The joinpoint regression analysis of the male age -standardized death rate for ischemic heart disease, (D) The joinpoint regression analysis of the female age -standardized death rate for ischemic heart disease, (E) The joinpoint regression analysis of the male age -standardized death rate for ischemic stroke (E) The joinpoint regression analysis of the female age -standardized death rate for ischemic stroke.

Abbreviation: CVD, cardiovascular disease. AAPC, average annual percent change. APC, annual percent change.

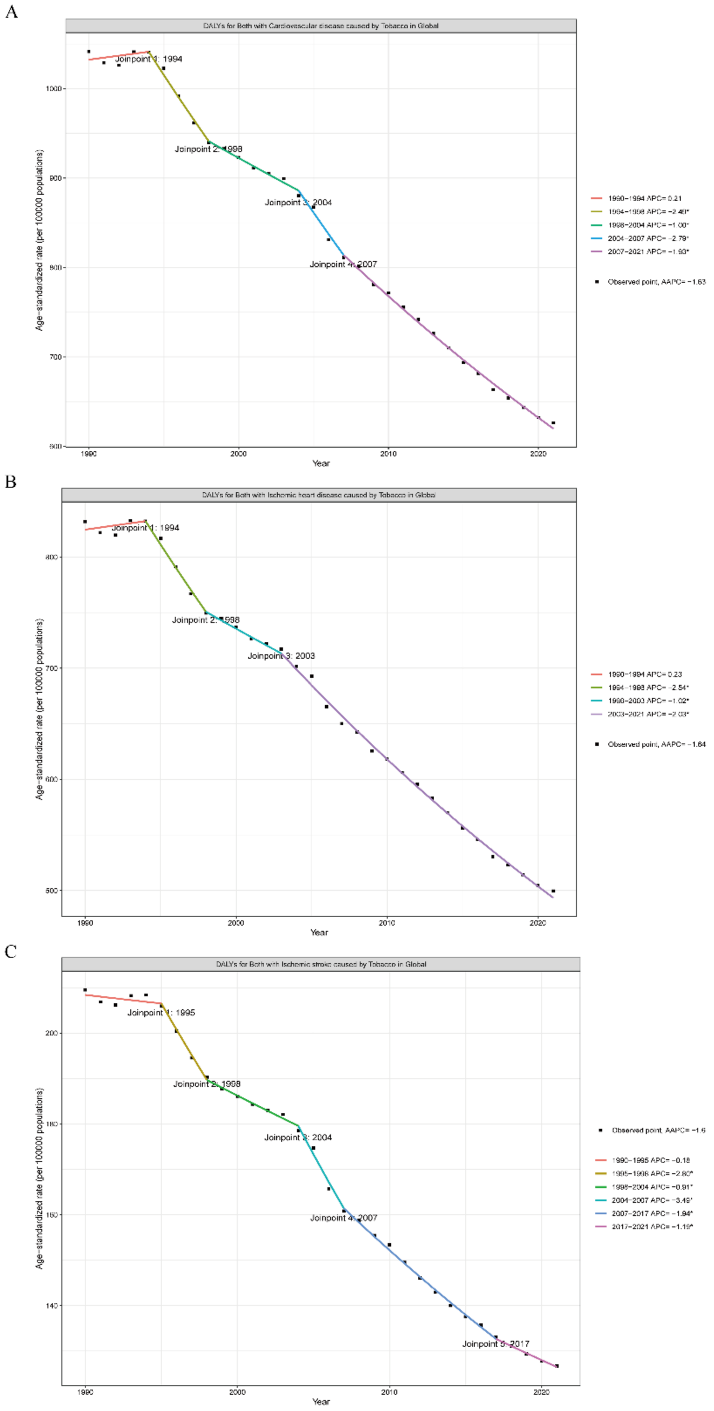

Figure S15. The joinpoint regression analysis of the age -standardized DALYs rate for CVD attributable to tobacco in global from 1990 to 2021. (A) The joinpoint regression analysis of the age -standardized DALYs rate for CVD, (B) The joinpoint regression analysis of the age -standardized DALYs rate for ischemic heart disease, (C) The joinpoint regression analysis of the age -standardized DALYs rate for ischemic stroke.

Abbreviations: CVD, cardiovascular disease. DALYs, disability-adjusted life years.

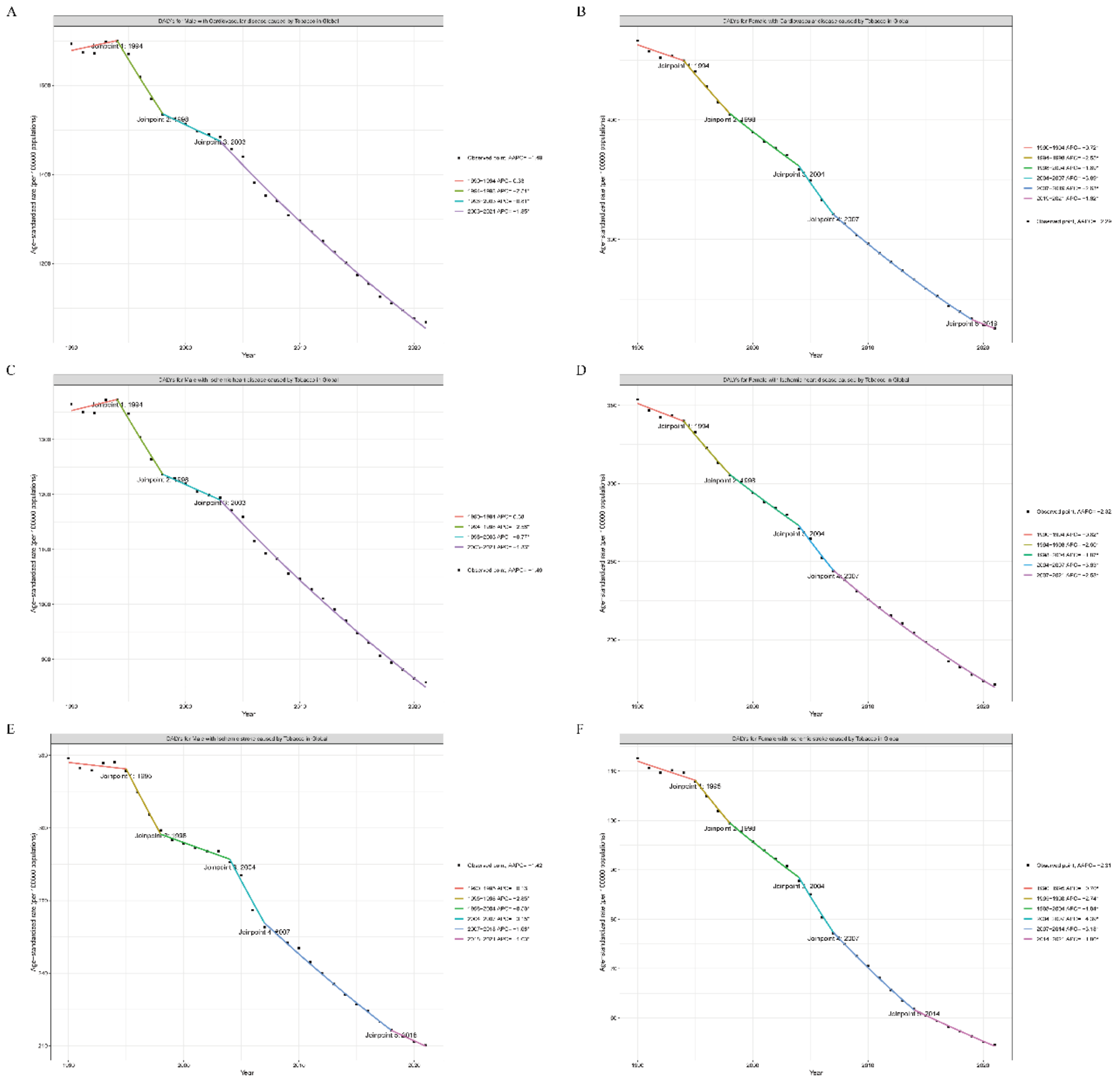

Figure S16. The joinpoint regression analysis of the sex-specific age-standardized DALYs rate for CVD attributable to tobacco in global from 1990 to 2021. (A) The joinpoint regression analysis of the male age -standardized DALYs rate for CVD, (B) The joinpoint regression analysis of the female age -standardized DALYs rate for CVD, (C) The joinpoint regression analysis of the male age -standardized DALYs rate for ischemic heart disease, (D) The joinpoint regression analysis of the female age -standardized DALYs rate for ischemic heart disease, (E) The joinpoint regression analysis of the male age -standardized DALYs rate for ischemic stroke (E) The joinpoint regression analysis of the female age -standardized DALYs rate for ischemic stroke.

Abbreviation: CVD, cardiovascular disease. AAPC, average annual percent change. APC, annual percent change. DALYs, disability-adjusted life years.

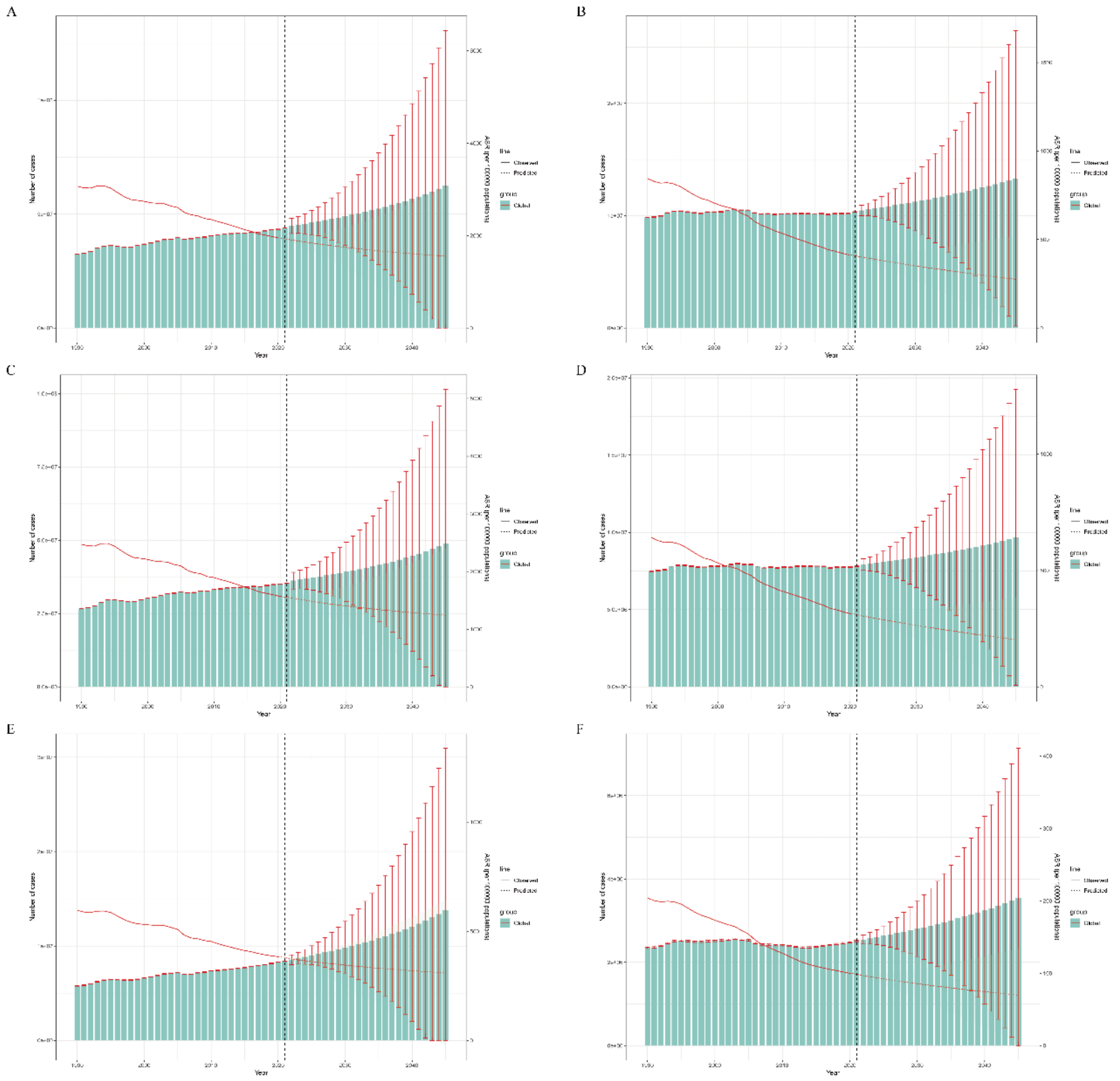

Figure S17. Temporal trend in the number of death cases and ASMR for CVD from 1990 to 2045 for both males and females. (A) Temporal trend in the number of death cases and ASMR for CVD for males, (B) Temporal trend in the number of death cases and ASMR for CVD for females, (C) Temporal trend in the number of death cases and ASMR for ischemic heart disease for males, (D) Temporal trend in the number of death cases and ASMR for ischemic heart disease for females, (E) Temporal trend in the number of death cases and ASMR for ischemic stroke for males, (F) Temporal trend in the number of death cases and ASMR for ischemic stroke for females.

Solid lines represent observed ASMR, and dashed lines represent ASMR predicted by the BAPC model. Abbreviation: ASMR, age-standardized mortality rate. CVD, cardiovascular disease. BAPC, Bayesian age-period-cohort.

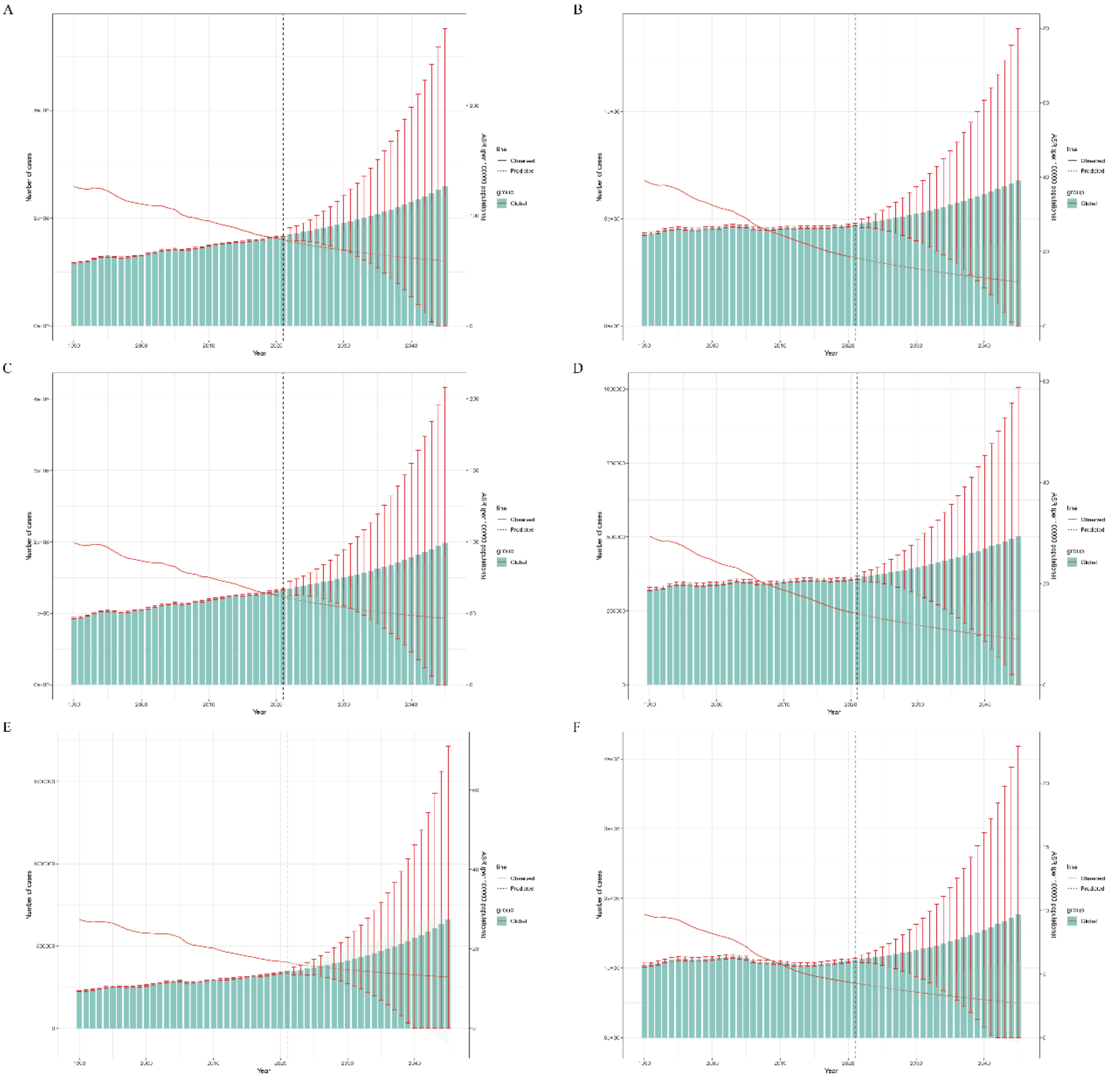

Figure S18. Temporal trend in the number of DALYs cases for CVD from 1990 to 2045 for both males and females. (A) Temporal trend in the number of DALYs and ASDR for CVD for males, (B) Temporal trend in the number of DALYs and ASDR for CVD for females, (C) Temporal trend in the number of DALYs and ASDR for ischemic heart disease for males, (D) Temporal trend in the number of DALYs and ASDR for ischemic heart disease for females, (E) Temporal trend in the number of DALYs and ASDR for ischemic stroke for males, (F) Temporal trend in the number of DALYs and ASDR for ischemic stroke for females.

Solid lines represent observed ASDR, and dashed lines represent ASDR predicted by the BAPC model.

Abbreviation: ASDR, age-standardized DALYs rate. CVD, cardiovascular disease. DALYs, disability-adjusted life years. BAPC, Bayesian age-period-cohort.

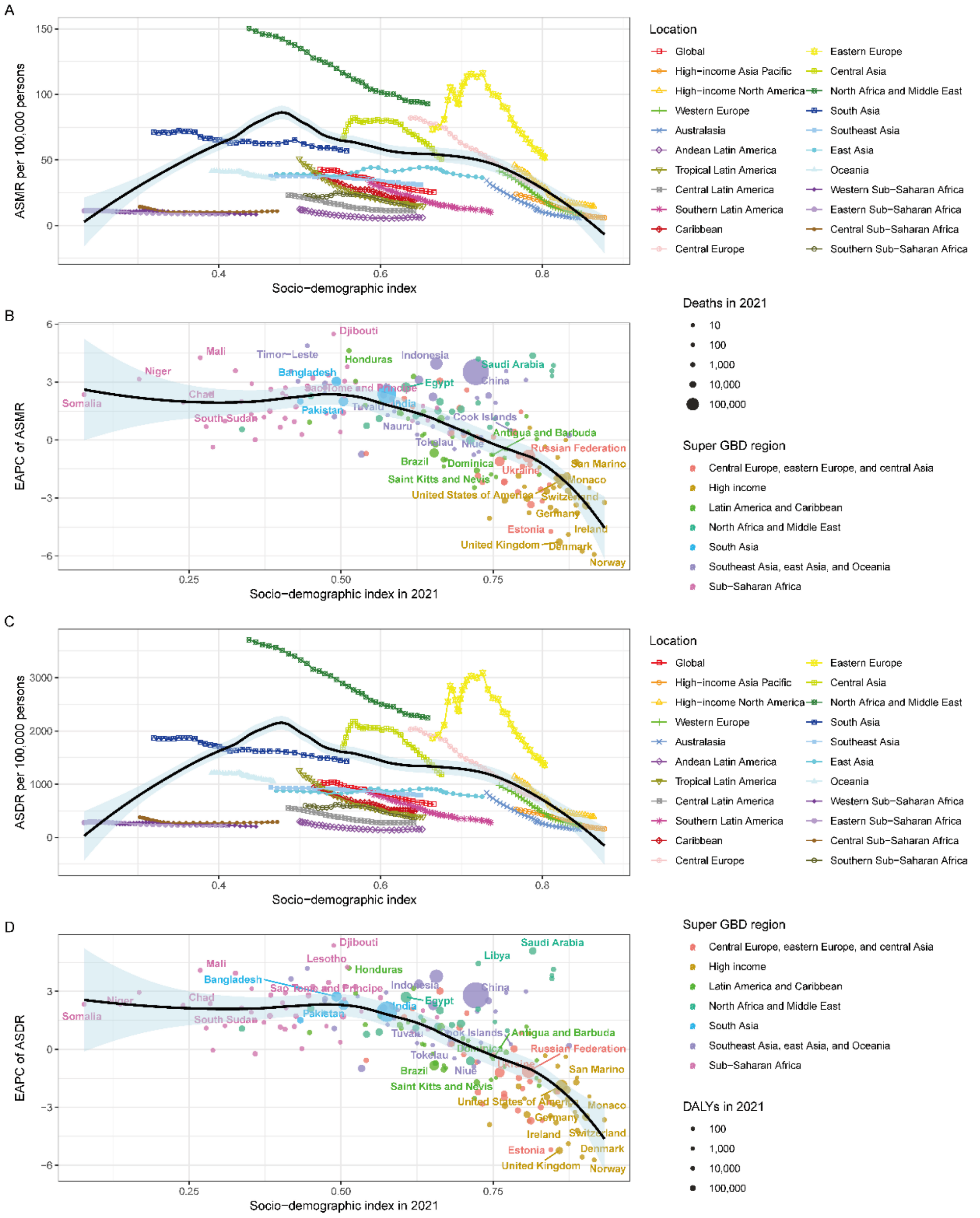

Figure S19. The change of ASMR and ASDR related to CVD attributable to tobacco with SDI changes. (A) ASMR and SDI by GBD location from 1990 to 2021, (B) EAPC of ASMR and SDI in 2021 by Super GBD region, (C) ASDR and SDI by GBD location from 1990 to 2021, (D) EAPC in ADMR and SDI in 2021 by Super GBD region

Abbreviations: CVD, cardiovascular disease. SDI, socio-demographic index. ASMR, age-standardized mortality rate. ASDR, age-standardized DALYs rate, DALYs, disability-adjusted life years, EAPC, estimated annual percentage change.

A

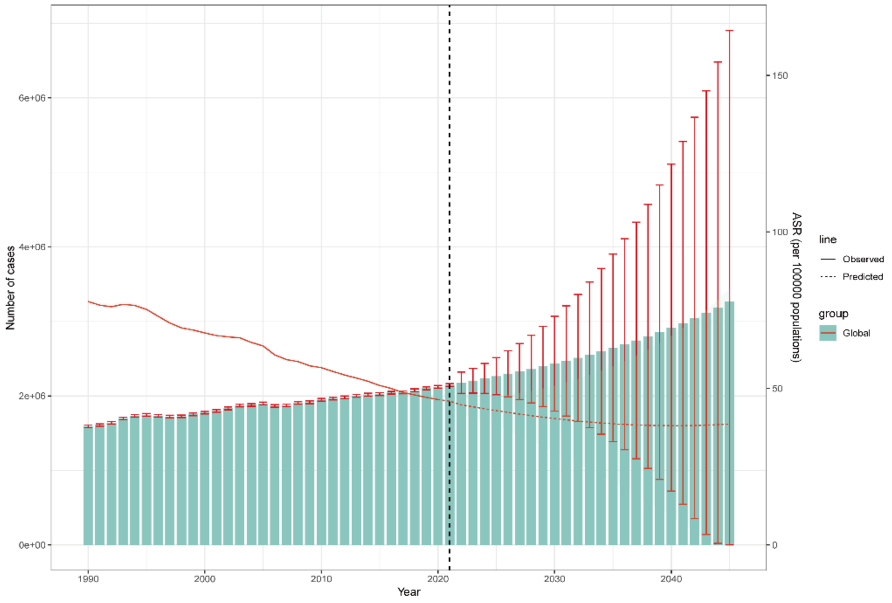
Number of cases
6e+06
4e+06
2e+06
0e+00
1990
2000
2010
2020
2030
2040
Year
ASR (per 100000 populations)
150
100
50
0
line
Observed
Predicted
group
Global

B

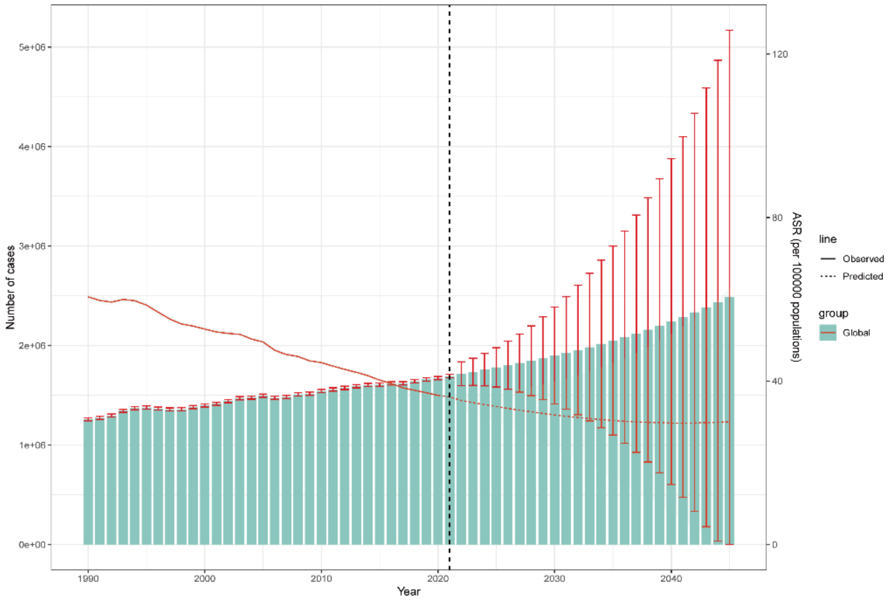
Number of cases
5e+06
4e+06
3e+06
2e+06
1e+06
0e+00
1990
2000
2010
2020
2030
2040
Year
ASR (per 100000 populations)
120
80
40
0
line
Observed
Predicted
group
Global

C

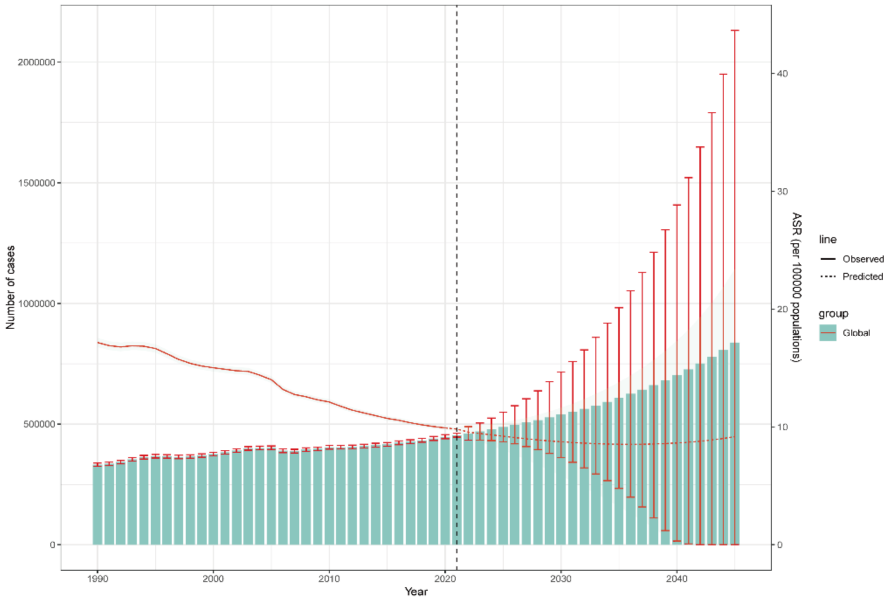
Number of cases
2000000
1500000
1000000
500000
0
1990
2000
2010
2020
2030
2040
Year
ASR (per 100000 populations)
40
30
20
10
0
line
Observed
Predicted
group
Global

Figure S20. Temporal trend in the number of mortality cases and ASMR for CVD from 1990 to 2045. (A) Temporal trend in the number of mortality and ASMR for CVD from 1990 to 2045, (B) Temporal trend in the number of mortality and ASMR for ischemic heart disease from 1990 to 2045, (C) Temporal trend in the number of mortality and ASMR for ischemic stroke from 1990 to 2045.

Solid lines represent observed ASMR, and dashed lines represent ASMR predicted by the BAPC model

Abbreviations: CVD, cardiovascular disease. ASMR, age-standardized mortality rate. BAPC, Bayesian age-period-cohort.

A

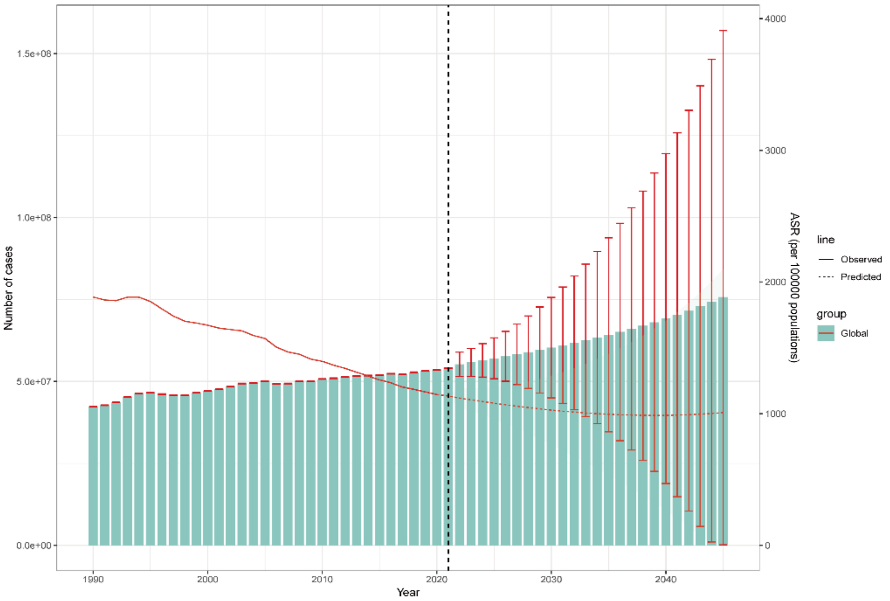

Number of cases
0.0e+00
5.0e+07
1.0e+08
1.5e+08
1990
2000
2010
2020
2030
2040
Year
ASR (per 100000 populations)
0
1000
2000
3000
4000
line
Observed
Predicted
group
Global

B

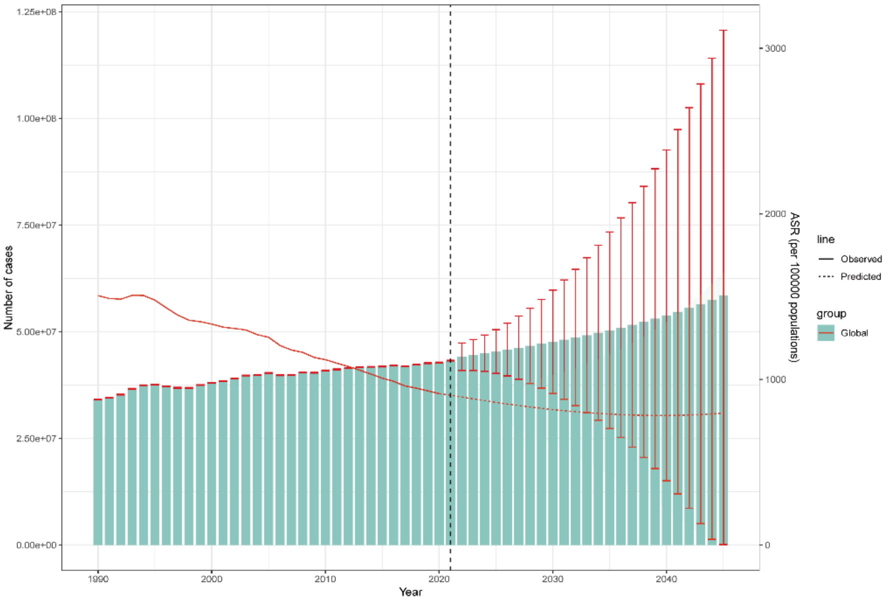

Number of cases
0.00e+00
2.50e+07
5.00e+07
7.50e+07
1.00e+08
1.25e+08
1990
2000
2010
2020
2030
2040
Year
ASR (per 100000 populations)
0
1000
2000
3000
line
Observed
Predicted
group
Global

C

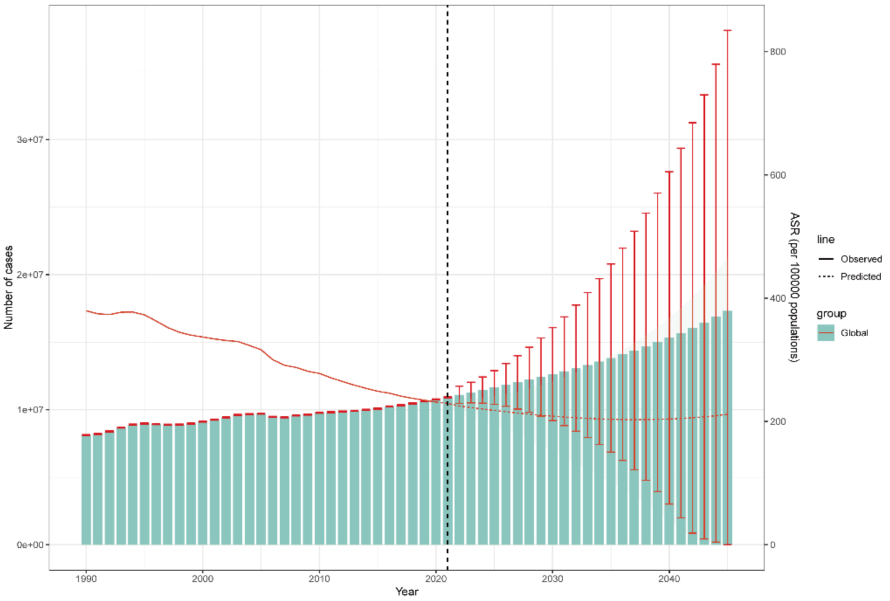

Number of cases
0e+00
1e+07
2e+07
3e+07
1990
2000
2010
2020
2030
2040
Year
ASR (per 100000 populations)
0
200
400
600
800
line
Observed
Predicted
group
Global

Figure S21. Temporal trend in the number of DALYs and ASDR for CVD from 1990 to 2045. (A) Temporal trend in the number of DALYs and ASDR for CVD from 1990 to 2045, (B) Temporal trend in the number of DALYs and ASDR for ischemic heart disease from 1990 to 2045, (C) Temporal trend in the number of DALYs and ASDR for ischemic stroke from 1990 to 2045.

Solid lines represent observed ASMR, and dashed lines represent ASMR predicted by the BAPC model

Abbreviations: CVD, cardiovascular disease. ASDR, age-standardized DALYs rate, DALYs, disability-adjusted life years, BAPC, Bayesian age-period-cohort.

© 2025 Zhu X. et al.
